# Kerley A-lines represent thickened septal plates between lung segments in patients with lymphangitic carcinomatosis: confirmation using 3D-CT lung segmentation analysis

**DOI:** 10.1007/s11604-021-01215-4

**Published:** 2021-11-09

**Authors:** Nanae Tsuchiya, Maho Tsubakimoto, Akihiro Nishie, Sadayuki Murayama

**Affiliations:** 1 Department of Radiology, Graduate School of Medical Science, University of the Ryukyus, 207 Uehara, Nishihara-cho, Nakagami-Gun, Okinawa, 903-0215 Japan; 2 Department of Radiology, Urasoe General Hospital, Okinawa, Japan

**Keywords:** Kerley's A lines, Interlobular septa, Lymphatics, Computed tomography, Lung segmentation

## Abstract

**Purpose:**

Kerley A-lines are generally apparent in patients with pulmonary edema or lymphangitic carcinomatosis. There are two main thoughts regarding the etiology of Kerley A-lines, but no general agreement. Specifically, the lines are caused by thickened interlobular septa or dilated anastomotic lymphatics. Our purpose was to determine the anatomic structure represented as Kerley A-lines using 3D-CT lung segmentation analysis.

**Materials and methods:**

We reviewed 139 charts of patients with lymphangitic carcinomatosis of the lung who had CT and X-ray exams with a maximum interval of 7 days. The presence of Kerley A-lines on X-ray was assessed by a radiologist. The A-lines on X-ray were defined as follows: dense; fine (< 1 mm thick); ≥ 2 cm in length, radiating from the hilum; no bifurcation; and not adjacent to the pleura. For cases with Kerley A-lines on X-ray, three radiologists agreed that the lines on CT corresponded with Kerley A-lines. The incidence of A-lines and the characteristics of the lines were investigated. The septal lines between lung segments were identified using a 3D-CT lung segmentation analysis workstation. The percentage of agreement between the A-lines on CT and lung segmental lines was assessed.

**Results:**

On chest X-ray, 37 Kerley A-lines (right, 16; left, 21) were identified in the 22 cases (16%). Of these, 4 lungs with 12 lines were excluded from analysis due to technical reasons. Nineteen of the 25 lines (76%) corresponded to the septal lines on CT. Of these, 11 lines matched with automatically segmented lines (intersegmental septa, 4; intersubsegmental septa, 7) by the workstation. Two lines (8%) represented fissures. Four lines corresponded to the bronchial wall/artery (3 lines, 12%) or vein (1 line, 4%).

**Conclusion:**

Kerley A-lines primarily represented thickened and continued interlobular septal lines that corresponded to the septa between lung segments and subsegments.

**Supplementary Information:**

The online version contains supplementary material available at 10.1007/s11604-021-01215-4.

## Introduction

In 1951, Kerley categorized linear shadows on chest X-ray as Kerley A-, B-, and C-lines [1, 2]. Kerley lines are present in patients with pulmonary edema, lymphangitic carcinomatosis, acute eosinophilic pneumonia, pneumoconiosis, and sarcoidosis, which can be diagnosed by chest X-ray or chest computed tomography (CT) [3]. For patients with interstitial pulmonary edema who are asymptomatic or only exhibit non-specific clinical findings, diagnostic imaging is an important tool. Delayed diagnosis of pulmonary interstitial edema can lead to alveolar pulmonary edema, which can be fatal and severe. Among the Kerley lines, the B-line is frequent and well known; however, the A-line is less frequent than the B-line and is often overlooked. One reason that the A-line is overlooked is that readers are not familiar with the anatomic structure that forms the A-line.

The Kerley A-line is a longer linear shadow in the upper lobes that is oriented from the hilum toward the pleura [1]. The Kerley B-line is a linear horizontal shadow in the costophrenic angle. There is a general consensus that the Kerley B-line represents interlobular septal thickening resulting from interstitial edema. In contrast, there are two main thoughts regarding the etiology of the Kerley A-line, but no general agreement. Kerley [2] and Felson [3] are of the opinion that the A-line represents dilated anastomotic lymphatics, while Heitzman [4] and Grainger [5] believe that the A-line represents thickened connective tissue septa.

In an effort to settle this difference of opinion regarding the Kerley A-line, we used a sophisticated 3- dimensional CT imaging technique. At the height of the research fervor involving the Kerley A-line, high-resolution CT was not available, thus pulmonary lymphangiography and pathologic specimens were used to address this question. Currently, we have high-resolution CT technology that enables us to obtain MPR images and 3D anatomic images, which together will contribute to revealing the identity of the Kerley A-line which has been a matter of debate for 70 years. We speculate that the A-line represents thickened septal plates between lung segments and intersegmental septa based on our daily clinical experience in interpreting chest CT scans of patients with pulmonary edema and lymphangitic carcinomatosis. Indeed, we support the “thickened lung connective tissue septum” proposed by Heitzman and Grainger. The purpose of this study was to determine whether the Kerley A-line consists of lung intersegmental septa using 3D-CT lung segmentation analysis.

## Materials and methods

### Subjects

**Fig. 1:**
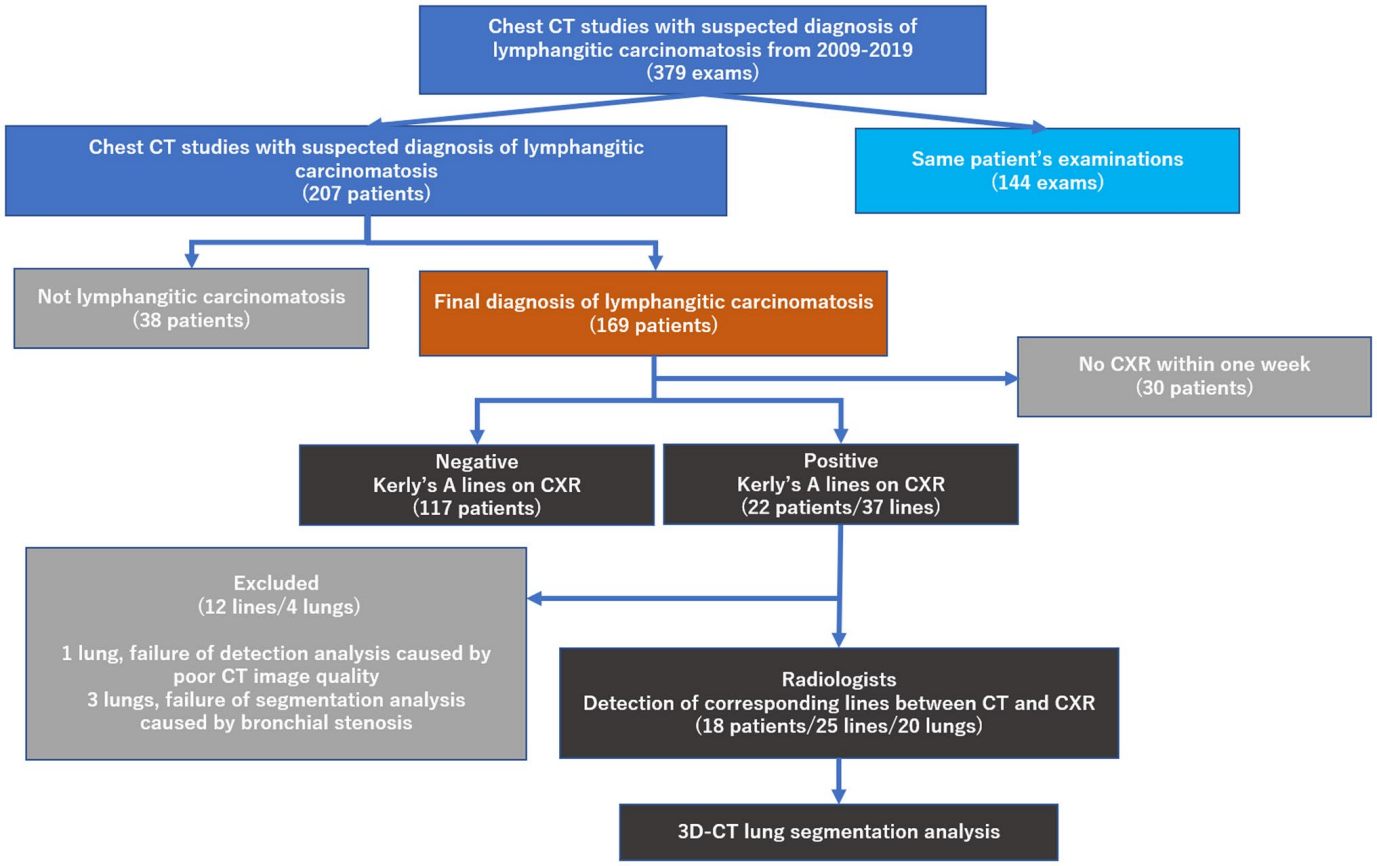
Study design and enrollment flow chart

This retrospective, cross-sectional study was approved by our institution’s Ethics Committee for Clinical Research with a waiver of informed consent. We studied consecutive patients who underwent chest CT with a suspected diagnosis of lymphangitic carcinomatosis from January 2009 to January 2019. An initial listing of the patient exams for this study was obtained through a search of the radiology picture archiving and communication system (PACS) and radiology information system (RIS). The final diagnosis of lymphangitic carcinomatosis was based on chest CT findings and clinical course. All chest CT exams were reported by board-certified radiologists and re-reviewed by a thoracic radiologist with 11 years of experience (N.T.) to confirm the diagnosis of lymphangitic carcinomatosis. All patients with a final diagnosis of lymphangitic carcinomatosis and who had chest CT and X-ray studies within 7 days were included in this analysis (Fig. 1). In cases based on the same examination, we selected the most aggressive phase images. The chest CT and chest X-ray were performed as part of the clinical evaluation with or without contrast media.

### X-ray and CT-Scan

Chest X-rays were obtained with a focus-to-film distance of 2 m with the following parameters: 120 kVp; 400 mA; automatic exposure control (AEC); 2.8mAs; 1.2 mm focal spot; 10:1 grid; and 52 lines/cm (DRX-3724H, Canon Medical Systems Corporation, Tochigi, Japan). Mobile X-rays were obtained with the following parameters: 90 kVp; 25 mA; 49 ms; 1.2mAs; and 6:1 virtual grid (Calneoaqro; Fujifilm Medical Corporation, Tokyo, Japan).

CT examinations were performed using two different scanners: a 64-row LightSpeed VCT scanner (GE Medical Systems, Milwaukee, WI, USA) and a 320-row Aquilion One scanner (Canon Medical Systems Corporation, Tochigi, Japan). CT scanning was performed during a breath-hold in full inspiration with the patient in the supine position. Scanning parameters were as follows: voltage, 120 kVp; current, AEC; collimation, 0.5 (One) or 0.625 mm (VCT); rotation time, 0.5 s; matrix, 512 × 512; and slice thickness, 1 (One) or 1.25 mm (VCT).

### Images

One thoracic radiologist (S.M. with 40 years of experience) blinded to the CT images reviewed the chest X-ray images and the presence or absence of the Kerley A-lines was determined. The A-lines on chest X-ray were defined based on the classic Kerley definition as follows: dense; fine (< 1 mm thick); ≥ 2 cm in length radiating from the hilum; no bifurcation; and not adjacent to the pleura (1, 2). The length of the A-line was measured. Another thoracic radiologist (N.T. with 11 years of experience) blinded to the chest X-ray images reviewed the CT images and the presence or absence of the thickened septal lines were determined.

Next, three thoracic radiologists (M.T., N.T., and S.M. with > 10 years of experience) compared the chest X-ray and CT images of the cases with Kerley A-lines independently to detect the corresponding anatomic structure of the Kerley A-lines. Discrepancies were resolved by consensus. CT images with a slice thickness < 1.5 mm were reviewed at a workstation (SYNAPSE; Fujifilm Medical Corporation, Tokyo, Japan). Any user-defined reconstructions were available for review, including axial images, multiplanar reformatted images (MPRs), maximum intensity projection (MIP), and average intensity projection (AIP).

**Fig. 2:**
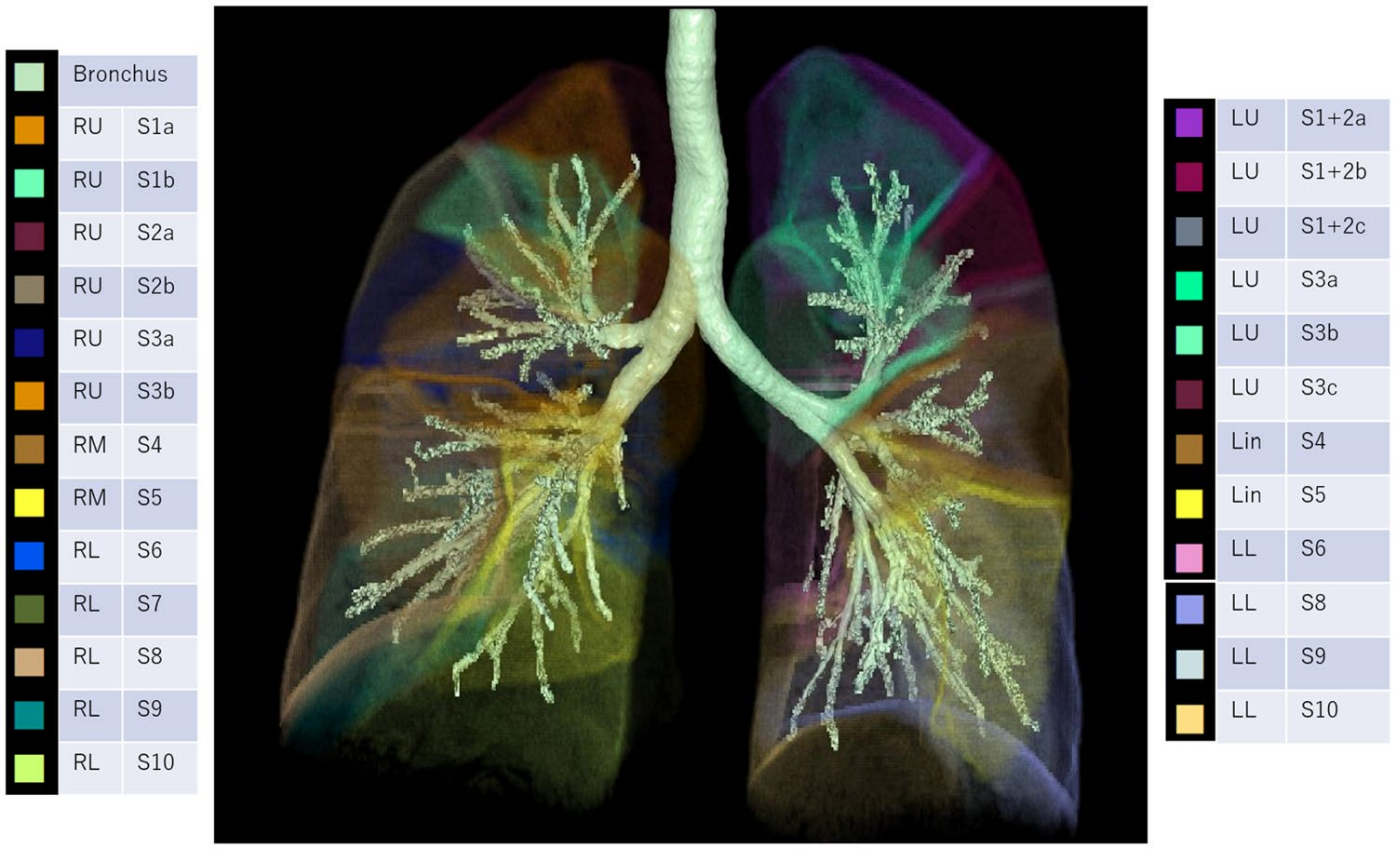
3D lung segmentation of a normal lung. The plate structure surrounding the lung segments of the upper lobes can be recognized as a linear shadow similar to the Kerley A-lines

3D-CT lung segmentation analysis was performed using a commercially available workstation (SYNAPSE VINCENT, version 4.1; Fujifilm Medical Corporation). Lung segmentation was based on bronchial segmentation. For bronchial segmentation, the fully-automated workstation extracted bronchial trees and a thoracic radiologist (N.T. with 11 years of experience) named each bronchus at the level of the fourth bronchial generation based on the definition described by Yamashita (Table 1) [6]. Lung segmentation was automatically extracted as each subsegmental lung area by the workstation based on the selected bronchial trees (Fig. 2).

**Table 1:** Nomenclature for lung structure

Right lung (primary branch)					Left lung (primary branch)				
Lobe	Segment		Subsegment		Lobe	Segment		Subsegment	
2nd branch	3rd branch		4th branch		2nd branch	3rd branch		4th branch	
Upper	Apical	S1	Apical	S1a	Upper	Apical-posterior	S1 + 2	Apical	S1 + 2a
			Anterior	S1b				Posterior	S1 + 2b
	Posterior	S2	Posterior	S2a				Horizontal	S1 + 2c
			Horizontal	S2b		Anterior	S3	Lateral	S3a
	Anterior	S3	Lateral	S3a				Medial	S3b
			Medial	S3b				Superior	S3c
Middle	Lateral	S4	–	–	Lingula	Superior	S4	–	–
	Medial	S5	–	–		Inferior	S5	–	–
Lower		S6 ~ S10	–	–	Lower		S6 ~ S10	–	–

## Results

Between January 2009 and January 2019, there were 379 chest CT studies (207 patients) with a suspected diagnosis of lymphangitic carcinomatosis. Of these studies, 169 patients were ultimately diagnosed with lymphangitic carcinomatosis. There were 139 patients who had a chest CT and chest X-ray within a 7-day interval.

On chest X-ray, 37 Kerley A-lines (right, 16; left, 21) were identified in the 22 patients (16%). On chest CT, there were 117 patients (84%) with thickened septal lines. Twenty of 22 patients (91%) had both Kerley A-lines on chest X-ray and thickened septal lines on chest CT (Table 2).

**Table 2:** Presence of Kerley A-lines on CXR and thickened septal lines on CT

	Thickened septal lines on CT		Total
	Positive	Negative	
Kerley A-lines on CXR			
Positive	20 (91%)	2 (9%)	22(16%)
Negative	65 (56%)	52 (44%)	117 (84%)
Total	85 (61%)	54 (39%)	139

*CXR *chest X-ray, *CT *computed tomography

**Fig. 3:**
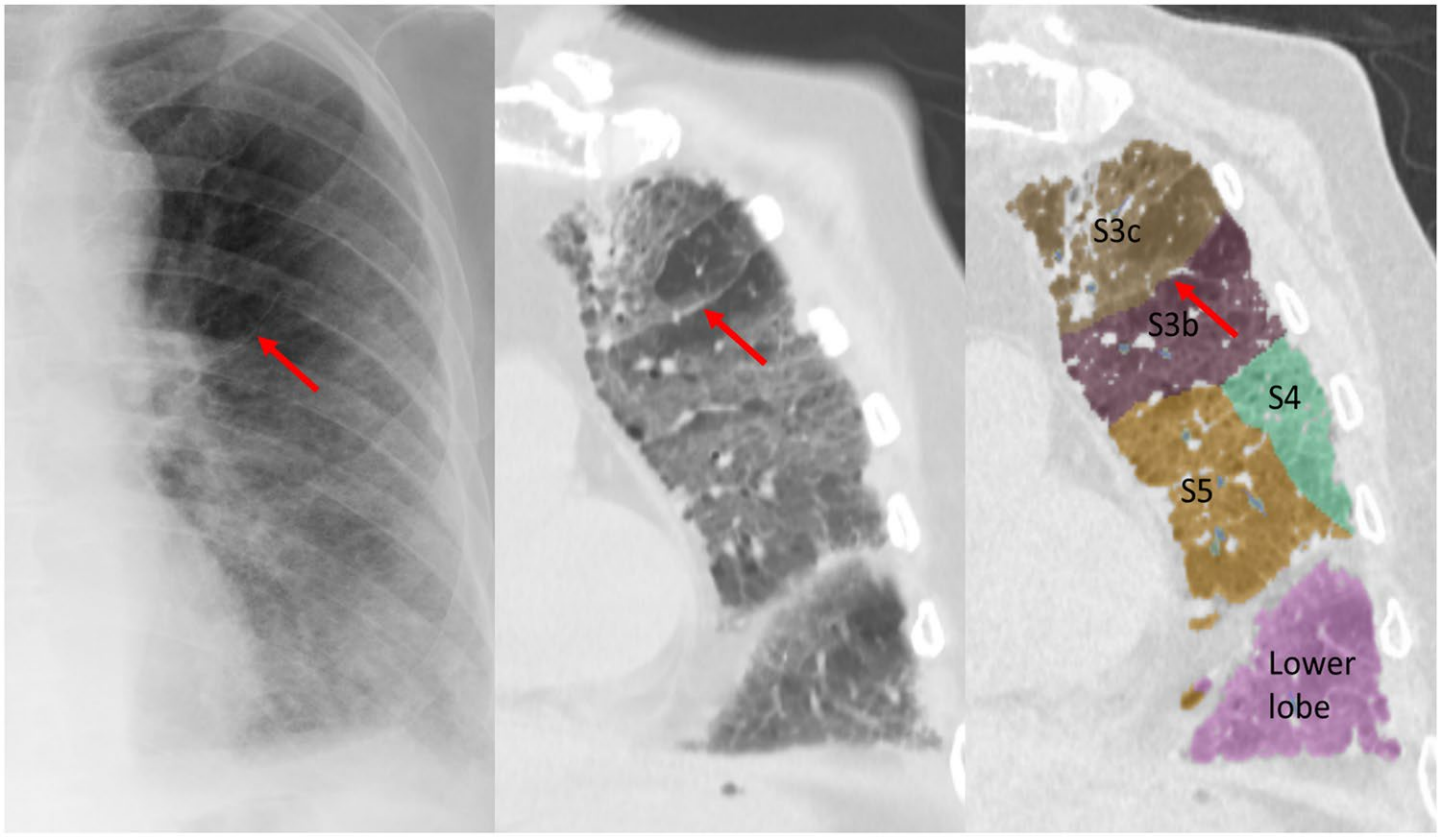
Case 6. Line 9. 88-year-old man with prostate cancer. Chest X-ray shows the Kerley A-line in the left lung. The line corresponds to the thickened septal line on CT and matched with the segmented lines between S3b and S3c by 3D segmentation analysis

**Fig. 4:**
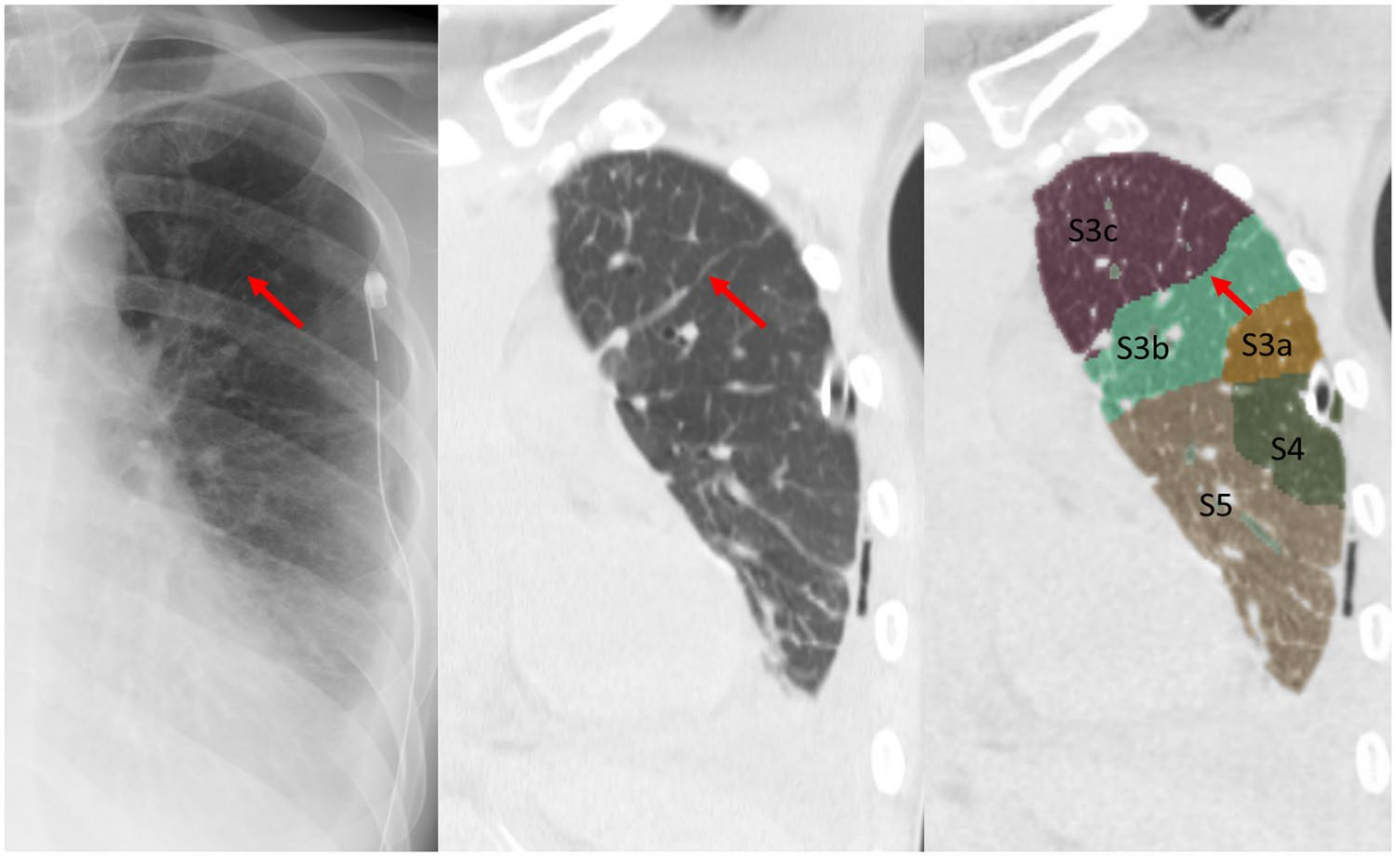
Case 5. Line 8. 35-year-old man with tongue cancer. Chest X-ray shows the Kerley A-line in the left lung. The line corresponds to the thickened septal line on CT and matched with the segmented lines between S3b and S3c by 3D segmentation analysis

**Fig. 5:**
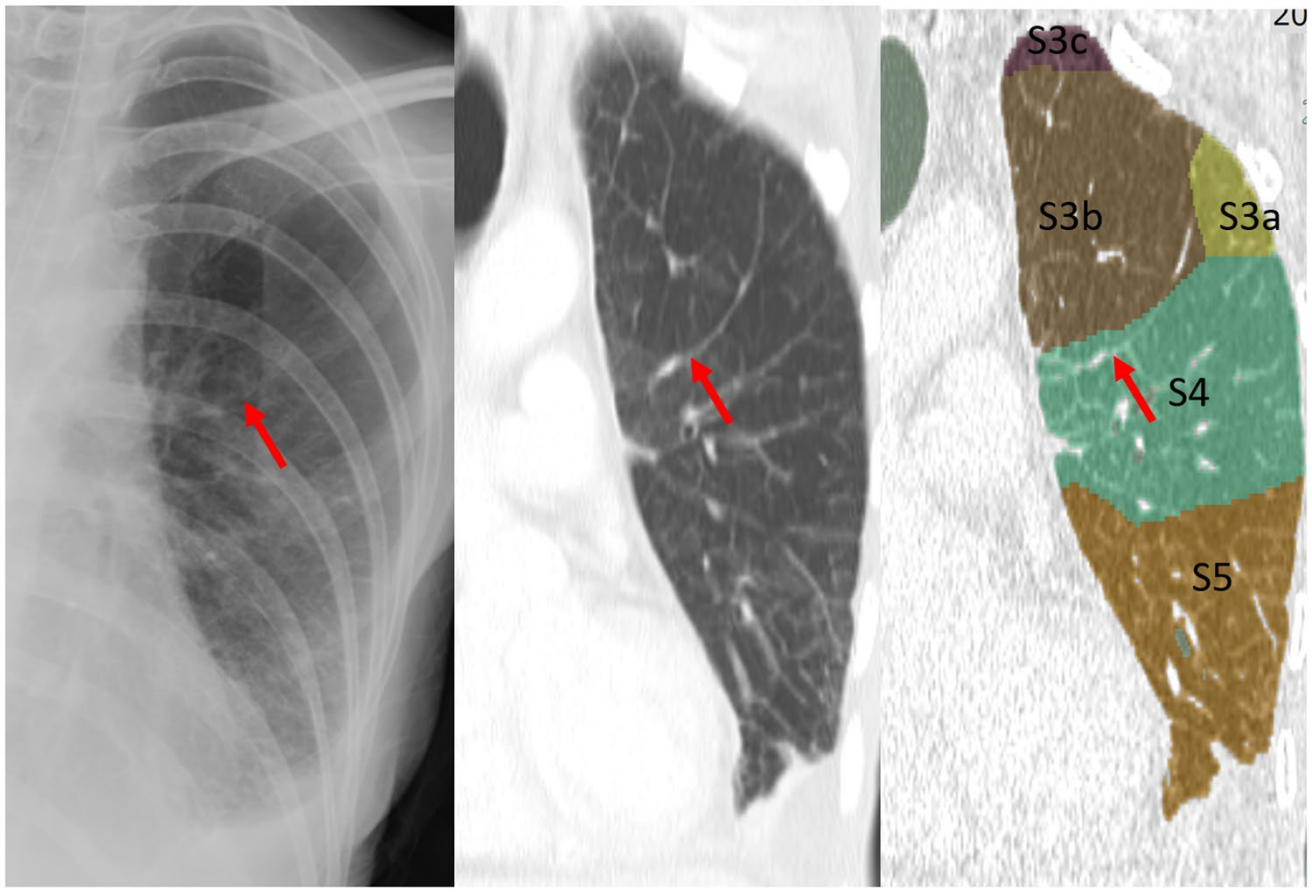
Case 3. Line 5. 51-year-old woman with gastric cancer. Chest X-ray shows the Kerley A-line in the left lung. The line corresponds to the thickened septal line on CT and matched with the segmented lines between S3b and S4 by 3D segmentation analysis

**Fig. 6:**
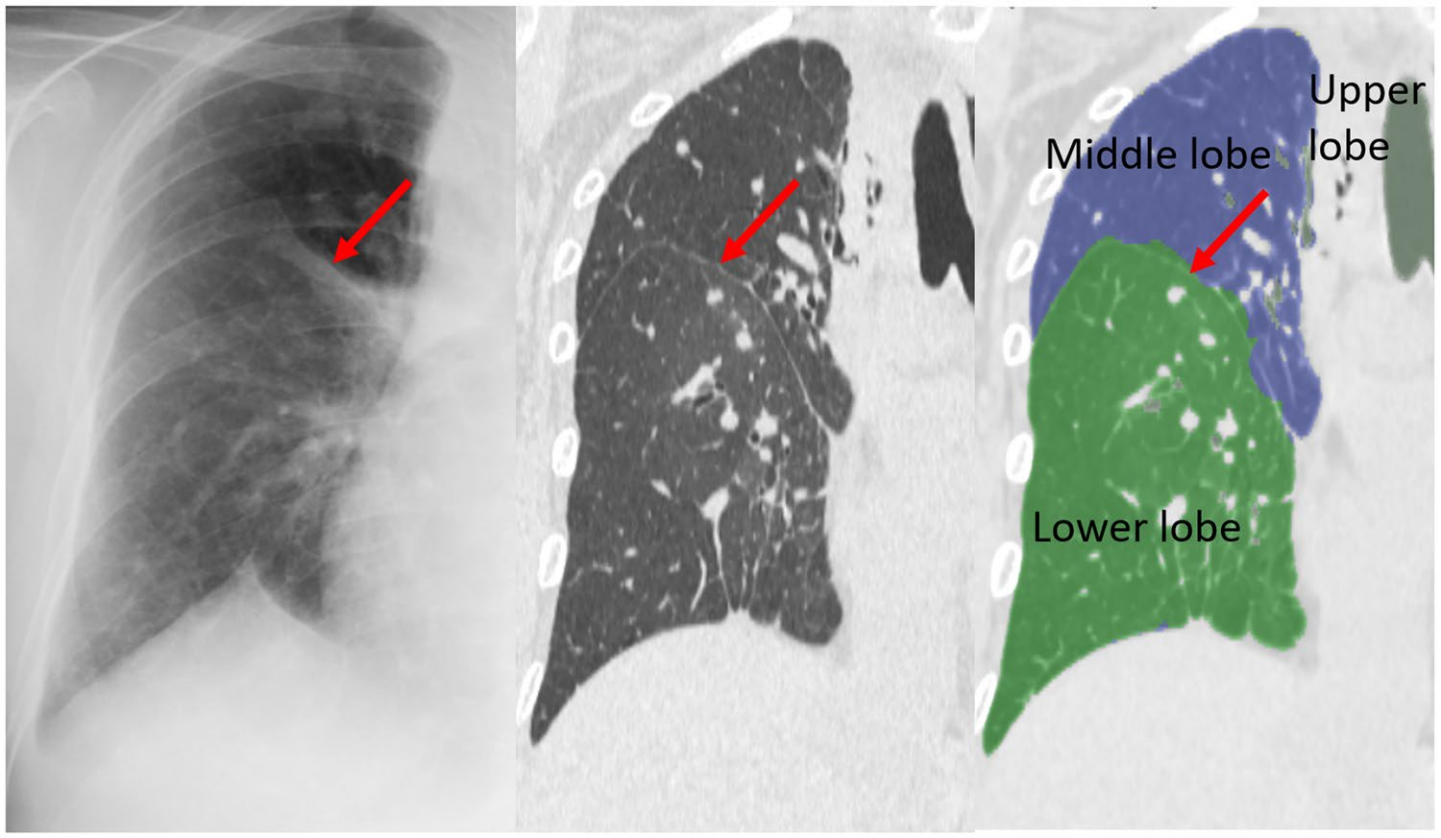
Case 8. Line 11. 51-year-old woman with gastric cancer. Chest X-ray shows the Kerley A-line in the right lung. The line corresponds to the deformed right major fissure on CT. The right upper lobe is complete atelectasis

**Fig. 7:**
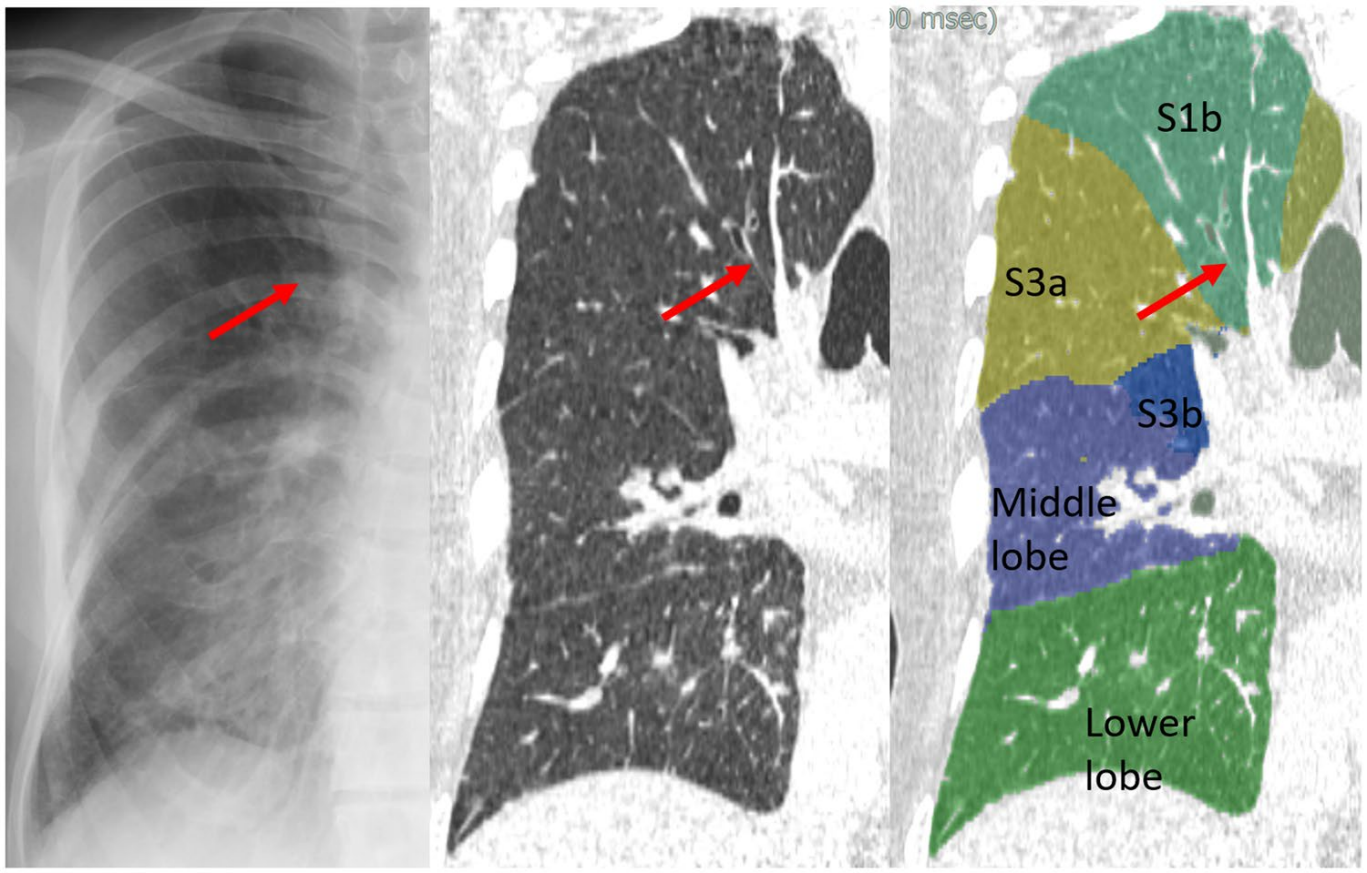
Case 10. Line 16. 66-year-old woman with breast cancer. Chest X-ray shows the Kerley A-line in the right lung. The line corresponds to the bronchial wall (B1b) and artery (A1b) on CT

There were 4 lungs with 12 lines that were excluded from further detection and segmentation analysis (3 lungs, failure of segmentation analysis due to bronchial stenosis; 1 lung, failure of detection analysis due to poor CT image quality). Twenty-five Kerley A-lines (right, 8; left, 17) were analyzed by the radiologists and the lung segmentation software to identify the corresponding anatomic structures on chest CT. The characteristics of Kerley A-lines are summarized in Tables 3 and 4. Nineteen of the 25 Kerley A-lines (76%) corresponded to the septal lines on CT. Fifteen of the 19 Kerley A lines on CT (79%) did not entendto the hilum. Thirteen of 19 Kerley A lines on CT (68%) were adjacent to the pleura. Of the 25 Kerley A-lines, 11 matched the automatically segmented lines (intersegmental septa, 4; intersubsegmental septa, 7) generated by the workstation. Most of the A-lines on the left side corresponded to the septal line between S3/S4 or S3b/S3c; representative cases are shown in Figs. 3, 4, 5. Another 2 A-lines (8%) represented fissures (Fig. 6). The other 4 A-lines corresponded to the bronchial wall/artery (3 lines, 12%; Fig. 7) or veins (1 line, 4%). The images of 25 Kerley A-lines include chest X-ray and chest CT, and segmentation analysis is shown in the supplemental figures (online resource: Figures S1–S25).

**Table 3:** Anatomic structure of Kerley A-lines on CT

	*N* = 25	%
Thickened septal line	19	76
Segment	5	20
Subsegment	9	36
Undersubsegment	5	20
Non-septal line	6	24
Fissure	2	8
Bronchial wall or artery	3	12
Vein	1	4

*CT* computed tomography

**Table 4:** Characteristics of Kerley A-lines

Case No	Age	Gender	Origin	Line No	Site	Length (mm)	Septal line on CT	Corresponding line on CT	Detail	Figure No	Match*
1	61	F	Malignant melanoma	1	R	4.1	Positive	Under subsegment	S3b	Fig S1	No
2	43	F	Colon cancer	2	L	6.8	Positive	Bronchial wall	B1 + 2b	Fig S2	No
3	51	F	Gastric cancer	3	L	2.5	Positive	Under subsegment	S3c	Fig S3	No
3	–	–	–	4	L	4.7	Positive	Subsegment	S3a/b/c	Fig S4	Yes
3	–	–	–	5	L	5	Positive	Segment	S3/S4	Fig S5	Yes
3	–	–	–	6	L	2	Positive	Subsegment	S3b/c	Fig S6	Yes
4	77	F	Lung cancer	7	L	6.5	Positive	Bronchial wall	B1 + 2c	Fig S7	No
5	35	M	Tongue cancer	8	L	5.4	Positive	Subsegment	S3b/c	Fig S8	Yes
6	88	M	Prostate cancer	9	L	4.1	Positive	Subsegment	S3b/c	Fig S9	Yes
7	70	M	Pancreatic cancer	10	L	4	Positive	Under subsegment	S3b	Fig S10	No
8	59	F	Lung cancer	11	R	3.2	Positive	Fissure	major fissure	Fig S11	Yes
9	58	F	Lung cancer	12	R	2.5	Positive	Segment	S1/S3	Fig S12	Yes
9	–	–	–	13	L	2.9	Positive	Subsegment	S1 + 2b/c	Fig S13	Yes
9	–	–	–	14	L	4.2	Positive	Subsegment	S3b/c	Fig S14	Yes
9	–	–	–	15	L	3.9	Positive	Under subsegment	S3b	Fig S15	No
10	66	F	Breast cancer	16	R	3.4	Negative	Bronchial wall	B1b	Fig S16	No
11	78	M	Lung cancer	17	R	5.2	Negative	Fissure	minor fissure	Fig S17	Yes
12	71	M	Rectal cancer	18	L	4.8	Positive	Segment	S3/S4	Fig S18	No
13	42	M	Renal cell cancer	19	L	4.5	Positive	Segment	S3/S4	Fig S19	Yes
14	59	F	Bladder cancer	20	L	3.4	Positive	Under subsegment	S3b	Fig S20	No
14	–	–	–	21	R	5.5	Positive	Subsegment	S1a/b	Fig S21	Yes
15	56	F	Renal cell cancer	22	R	4.2	Positive	Subsegment	S1	Fig S22	No
16	65	F	Colon cancer	23	R	4.4	Positive	Vein	Vein (S2a/S2b)	Fig S23	No
17	54	F	Ovarian cancer	24	L	2	Positive	Segment	S3/S4	Fig S24	Yes
18	48	F	Lung cancer	25	L	2.1	Positive	Subsegment	S3	Fig S25	No

*F* Female, *M* Male, *R* Right, *L* Left

*Match between the line identified by radiologists and segmented lines by the 3D-lung segmentation analysis software

## Discussion

In this study, we showed that most Kerley A-lines consisted of lung intersegmental/subsegmental septa in lymphangitic carcinomatosis patients using 3D-CT lung segmentation analysis. The presence of intersubsegmental septa has not been confirmed in the literature, but the results of this study support the presence of intersubsegmental septa.

In 1951 Kerley definedlonger linear shadows in the upper lobes, oriented from the hilum toward the pleural as Kerley A-lines in the 2nd edition of the book entitled *A Text-book of X-ray Diagnosis* [1]. Kerley’s final opinion regarding the Kerley A-lines was described in the 4th edition of the same book in 1972 [2]. The final description of A-line by Kerley was “The deep lymphatics are not so often seen. They are similar in appearance to the intercommunicating ones but run for as long a 7.5–10 cm. without a bifurcation, and of course stop short of the edge of the lung. This long uninterrupted course shows that they are not running in the interlobular septa nor following a peribronchial or periarterial course” [2]. Based on this description, the concept of lung intersegmental septa was uncommon in the era. Several studies described the connective tissue septa in the lung with subdivided lobes, but did not clearly associate it with the lung segments [7, 8]. Naganishi described intersegmental septa in 1972 [9]. In 1978 Yamashita reported detailed anatomic variations in the pulmonary segments and the bronchovascular trees [6]. In this paper, Yamashita determined the location of intersubsegmental veins, which suggested the presence of intersubsegmental septa. In 2020 Oikado speculated that lung intersubsegmental septa exist [10]. Our study showed 76% of the A-lines coincided with thickened septal lines, which were plate structures rather than line structures. In addition, the plate structures corresponded to intersegmental or intersubsegmental septa in the upper lung. These lines are a series of interlobular septa that contain a branch of the pulmonary vein surrounded by a plexus of lymphatics and capillaries forming a cordon of lymphatics [5]. Usually, these lines cannot be seen. When lymphatic flow is impaired, such as in lymphangitic carcinomatosis, the interlobular lymphatics are dilated with stasis, leading to thickened interlobular septa [5]. When the pressure of the pulmonary veins and capillaries increases, such as in pulmonary edema, increased transudation of fluid from the pulmonary capillaries into the pulmonary stroma causes thickened interlobular septa [5]. This is the mechanism underlying A-line formation.

The A-line on chest X-ray radiated from the hilum; however, many A-lines on CT did not continue to the hilum. The A-lines on chest X-ray were not adjacent to the pleura; however, many A-lines on CT scans were adjacent to the pleura. The reason for these findings was that the septal line between the upper lobe segment, which corresponded to the Kerley A-line, ran straight and extended obliquely and medially from the anterior chest wall on the anteromedial lung portion. Berkman et al. [11] compared the X-ray and CT findings of the accessory fissure in the left upper lobe and classified the accessory fissure into four types; type 3, which separates S3 and S4, was similar to the A-line we identified in this study.

The incidence of Kerley A-lines on chest X-ray in patients with lymphangitic carcinomatosis was 16%. The incidence of thickened septal lines on CT in patients with lymphangitic carcinomatosis was 84%. Even if the septal line was visible on CT, it was not always possible to visualize the A line on chest X-ray. Whether the A line was or was not noted was due to the variation between septal lines (variation in lung segmentation). The lung segment septum was not constant across all segments and may or may not be present during the development of the lung. The presence of accessor and incomplete fissures supports this notion. In contrast, even though there was no pulmonary stasis on CT, the A-line on X-ray, a sign of pulmonary congestion, was highlighted in this study. The incidence of these pseudo-A-lines which were formed by the bronchial wall, arteries, fissures, and veins was 9%.

Although this study supports the interlobular theory, we do not dismiss the dilated lymphatics theory because lymphatic vessels are one of the structures that comprises the interlobular septum. The historic investigators of the Kerley A-lines also accepted the alternative hypothesis, in part, and advocated their own theories, as we have done. As Grainger concluded [5], it is more rational to think that the structure that makes up the clear line and dense A-line is not a single tubular structure, but can be detected when the plate structure forms a tangent with X-rays. Therefore, the lung segmental septum includes deep lymphatics, as shown on lymphatic injection of lungs by Trapnell [12], but the lymphatics do not always fit directly into the A-line.

This study had several limitations. First, there were differences in examination dates, position (standing or supine and hands up or down) and the level of inspiration between chest X-ray and CT. Second, lung segment nomenclature is not perfect because there are normal variations in the bronchial bifurcation. In this study, we used lung segmentation based on the bronchial bifurcation because most of the available CT scans were non-contrast CT scans. The results may differ with lung segmentation based on vascular bifurcation. Third, we did not include short lines oriented at the hilum (< 2 cm), which is more frequently experienced clinically as A-lines. These lines are likely to be the short interlobular septum, rather than the intersegmental septa [13]. Whether this short line should be an A- or B-line is controversial. Although the short line was excluded from this study, the short line is considered to be close to the B-line if classified based on anatomic structure. Finally, we did not determine the incidence and evaluate the image findings of Kerley A lines in patients with other diseases, such as pulmonary edema and, healthy subjects.

In conclusion, Kerley A-lines represent thickened and continued interlobular septal lines that correspond to the septal plate between lung segments or subsegments. The bronchial wall, arteries, fissures, and veins may be mistaken for a pseudo-A-line with a 9% incidence when defining a true A-line as a thickened lung segment septum. In this study, to celebrate the 70th anniversary of the Kerley A-line report, we assessed the validity of the Kerley A-line in making a final decision.

## Supplementary Information

Below is the link to the electronic supplementary material.

Supplementary file1 (PDF 3747 KB)
