# Supplementary material for: Kerley A-lines represent thickened septal plates between lung segments in patients with lymphangitic carcinomatosis: confirmation using 3D-CT lung segmentation analysis
Source: Jpn J Radiol. 2021 Nov 9;40(4):367–75. doi: 10.1007/s11604-021-01215-4 (PMC8977263; doi:10.1007/s11604-021-01215-4)

Fig S1a

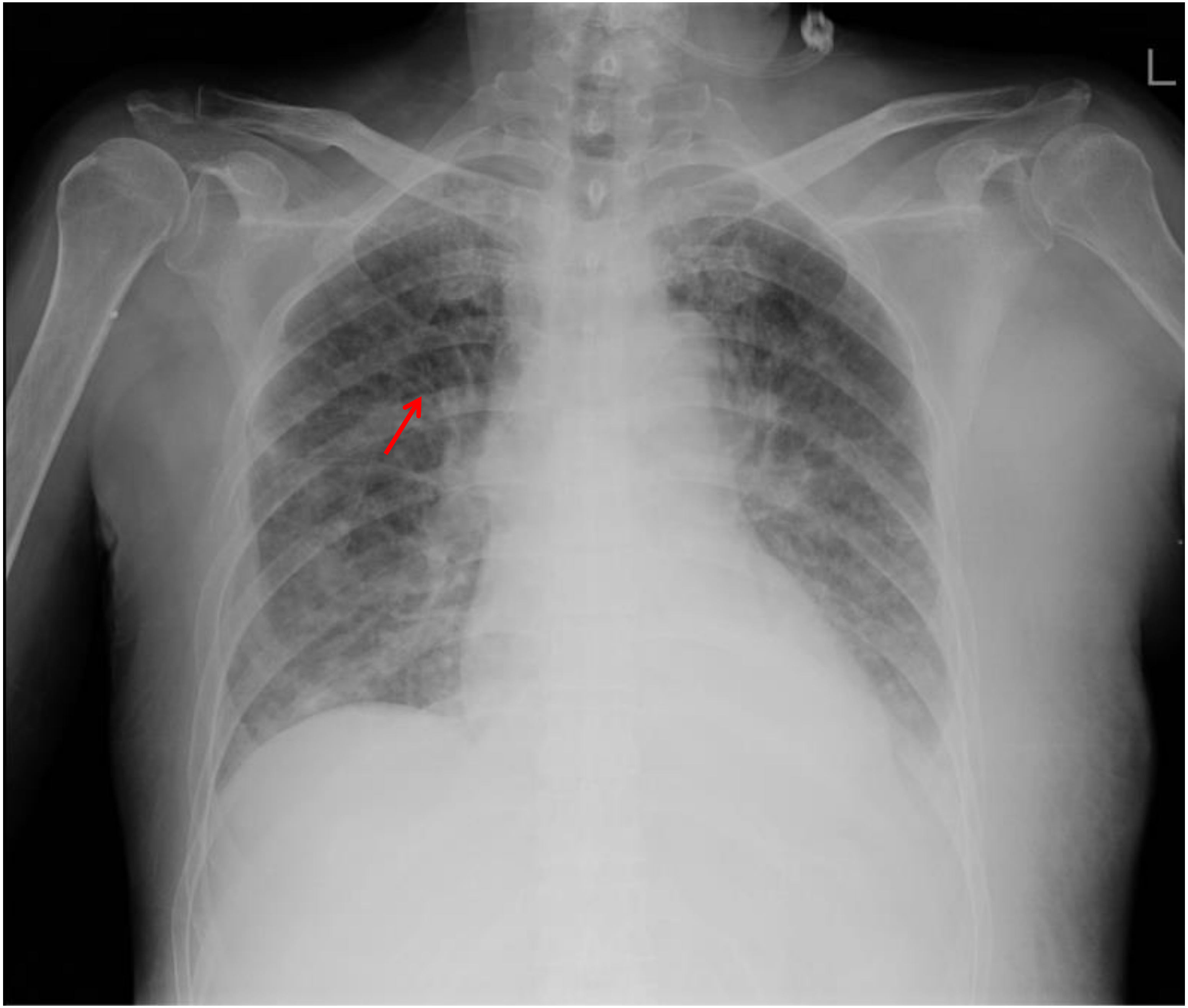

Fig S1b

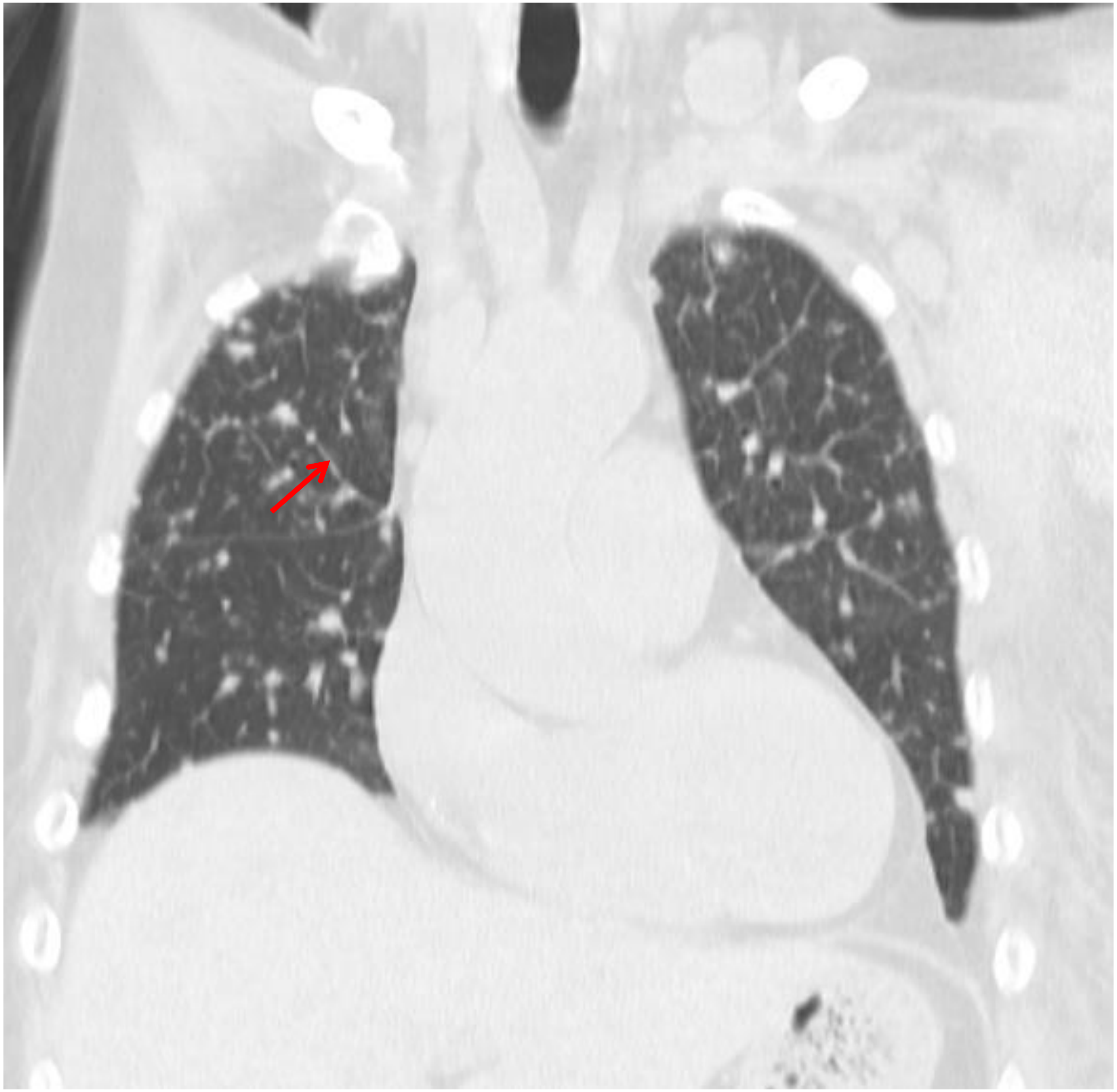

Fig S1c

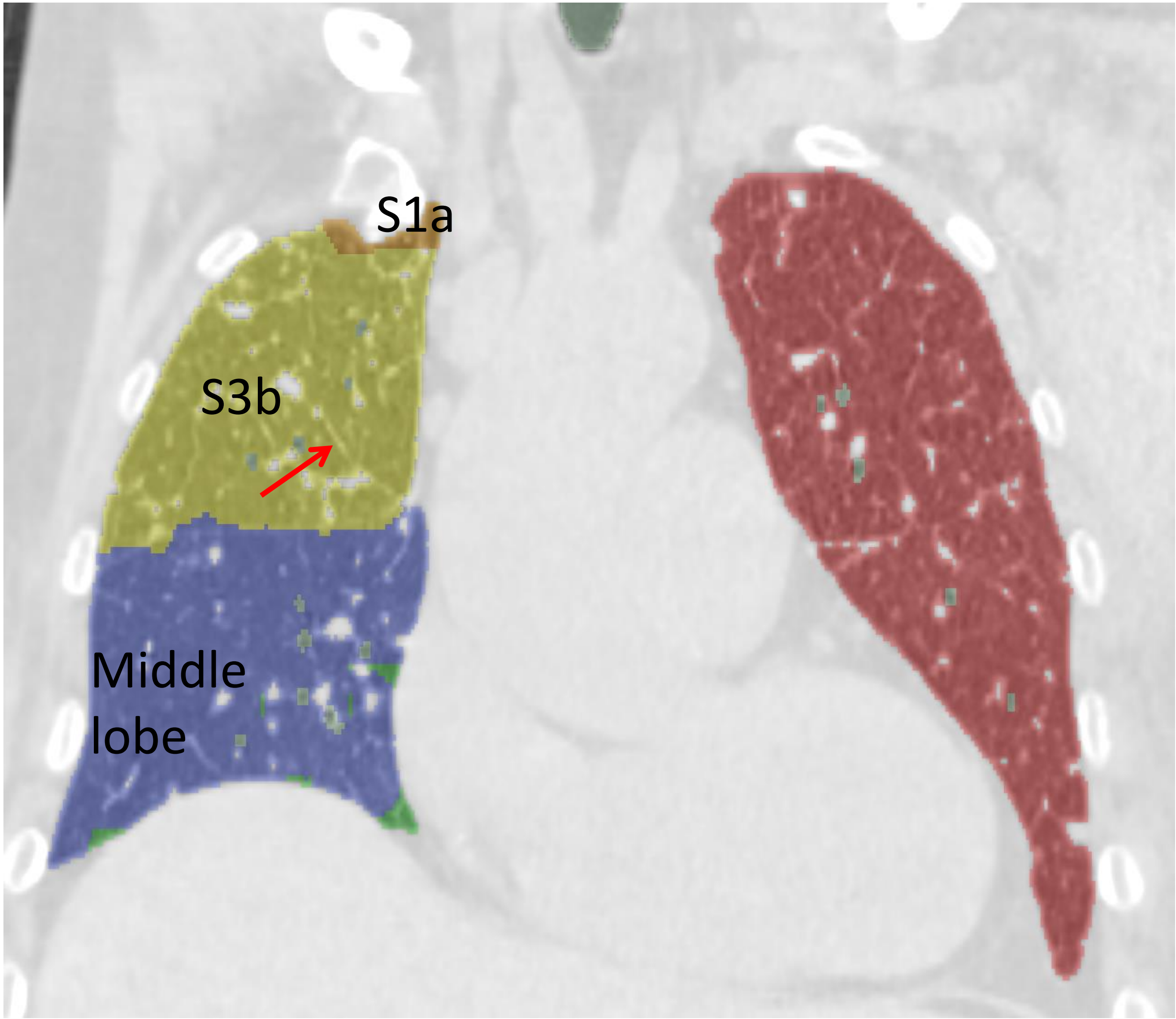

Fig S2a

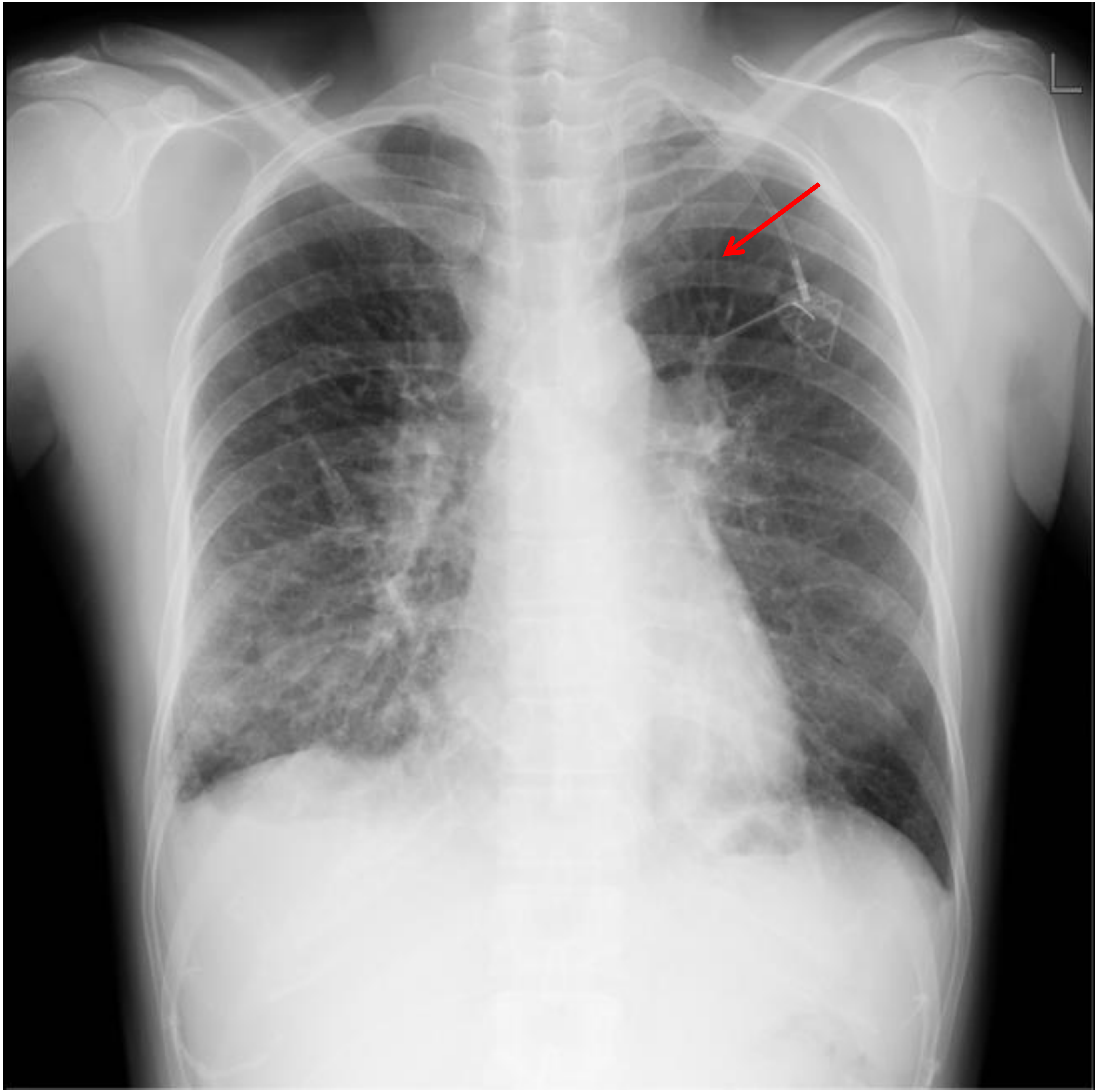

Fig S2b

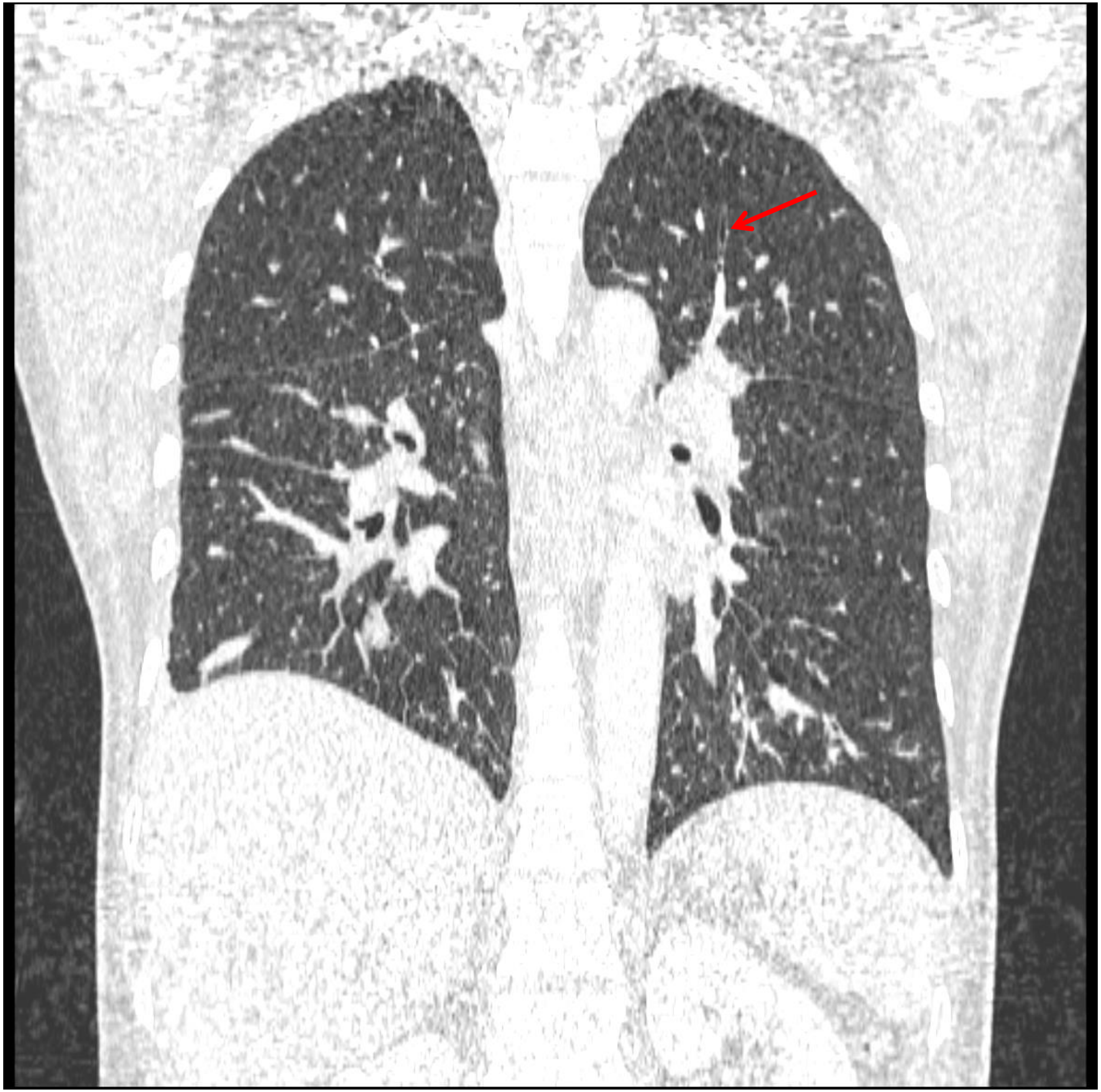

Fig S2c

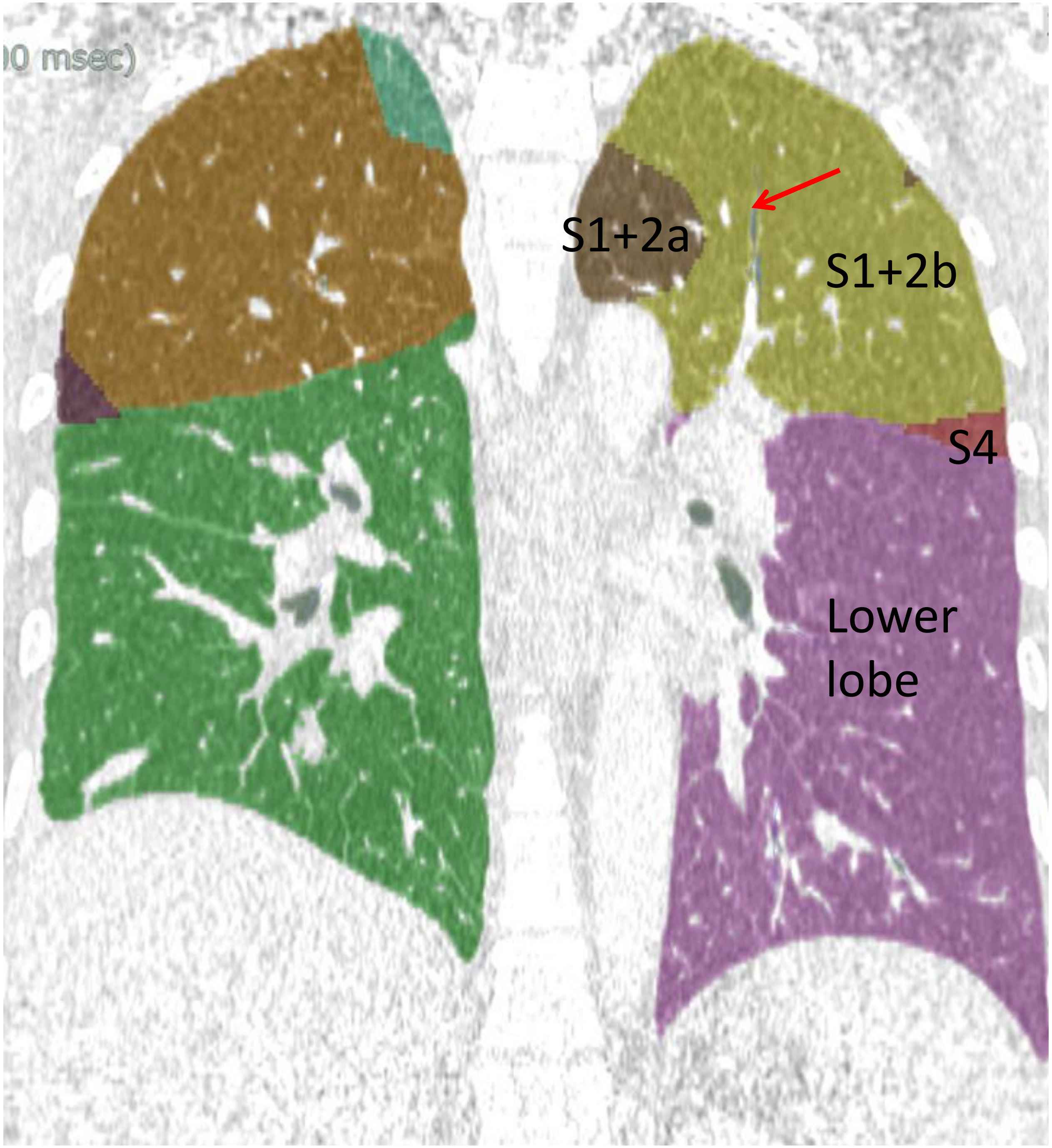

Fig S3a

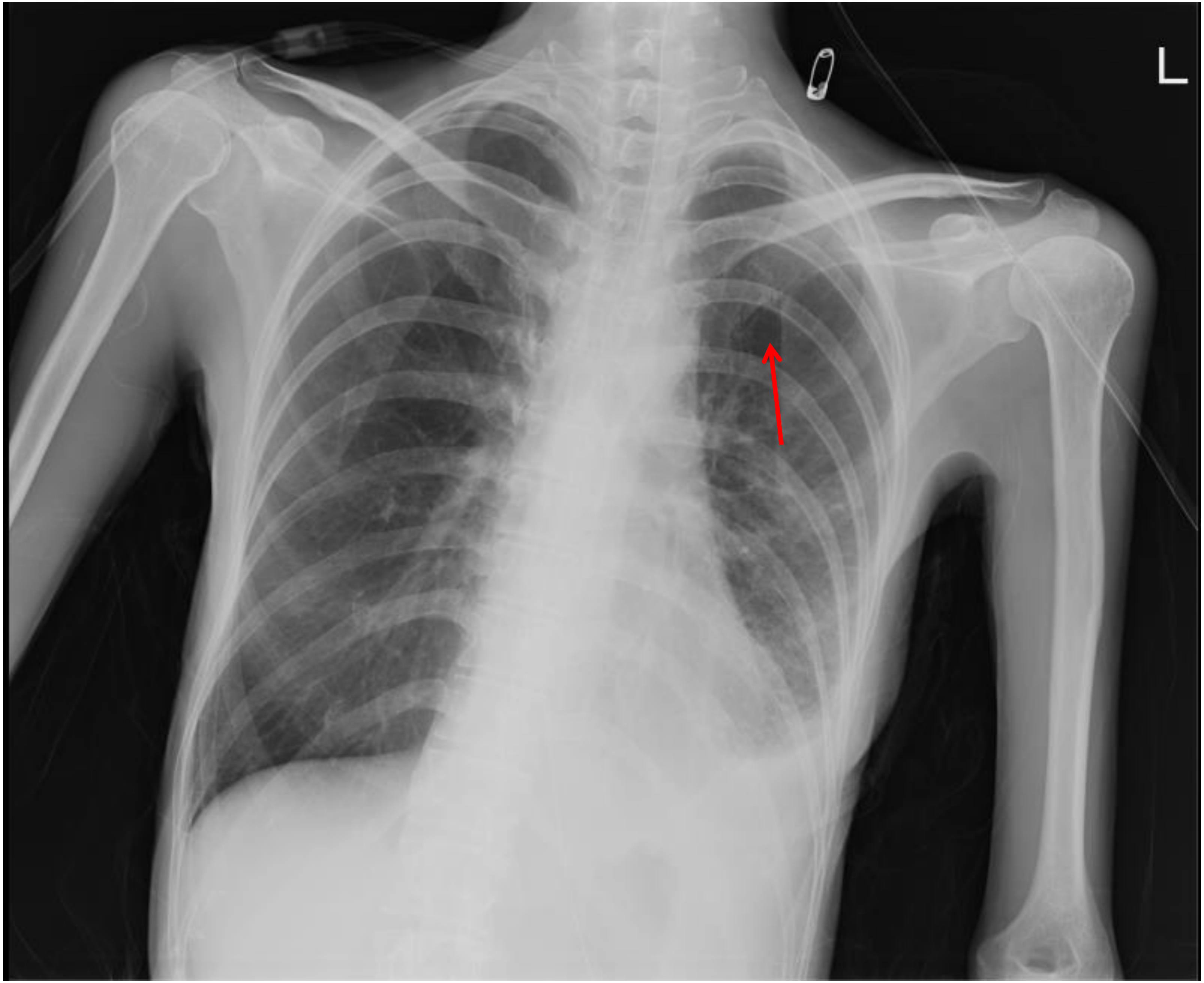

Fig S3b

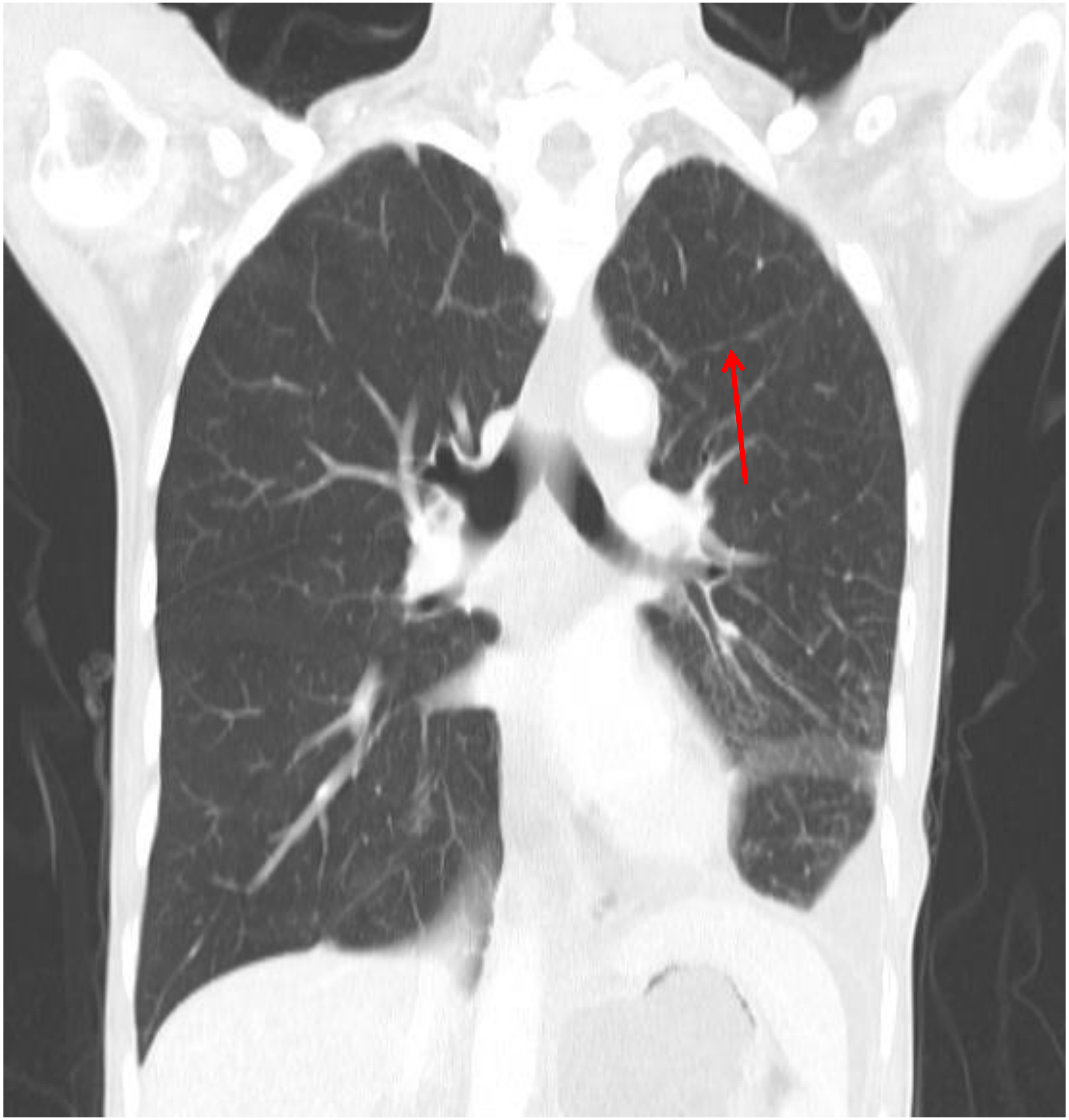

Fig S3c

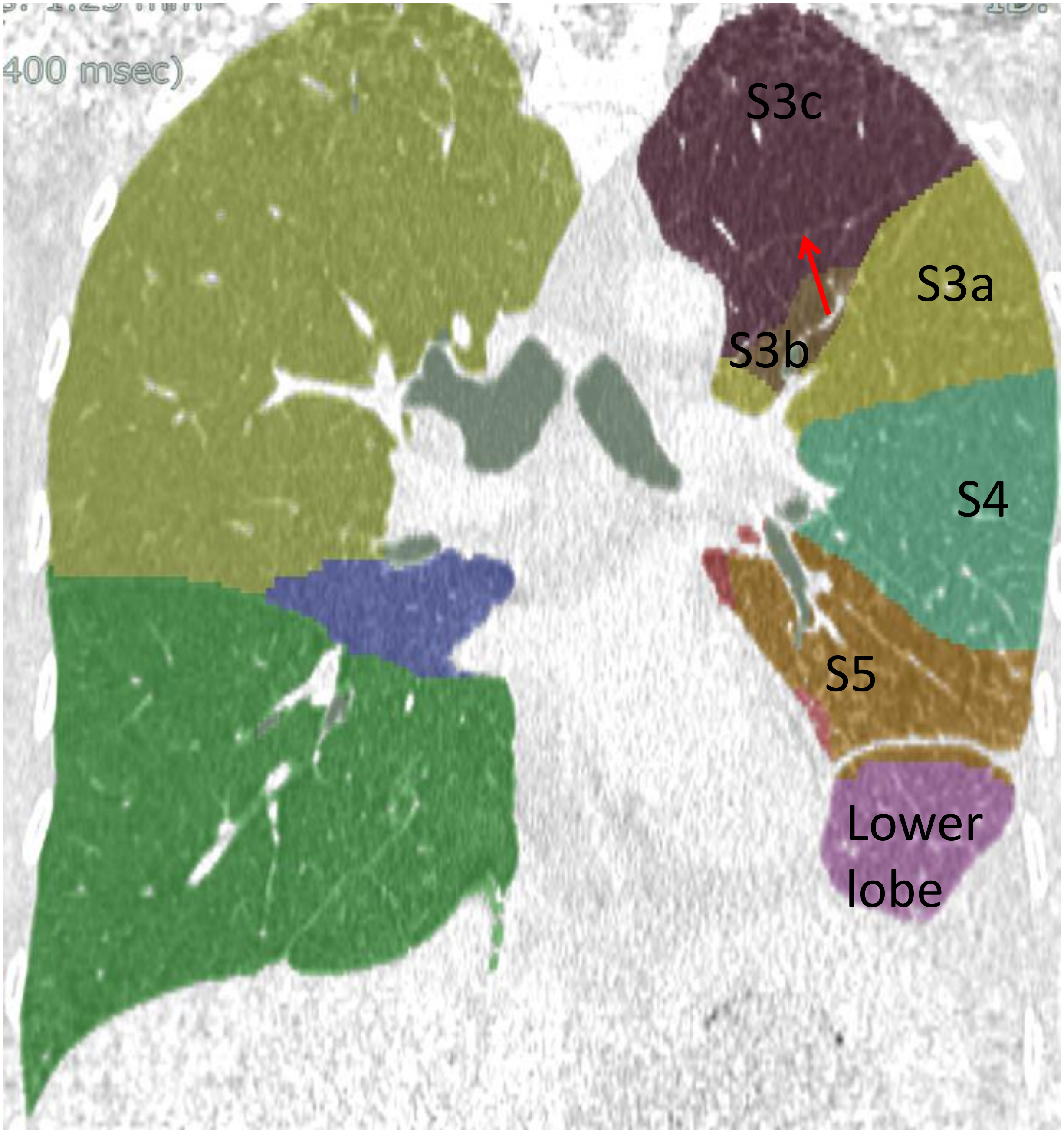

Fig S4a

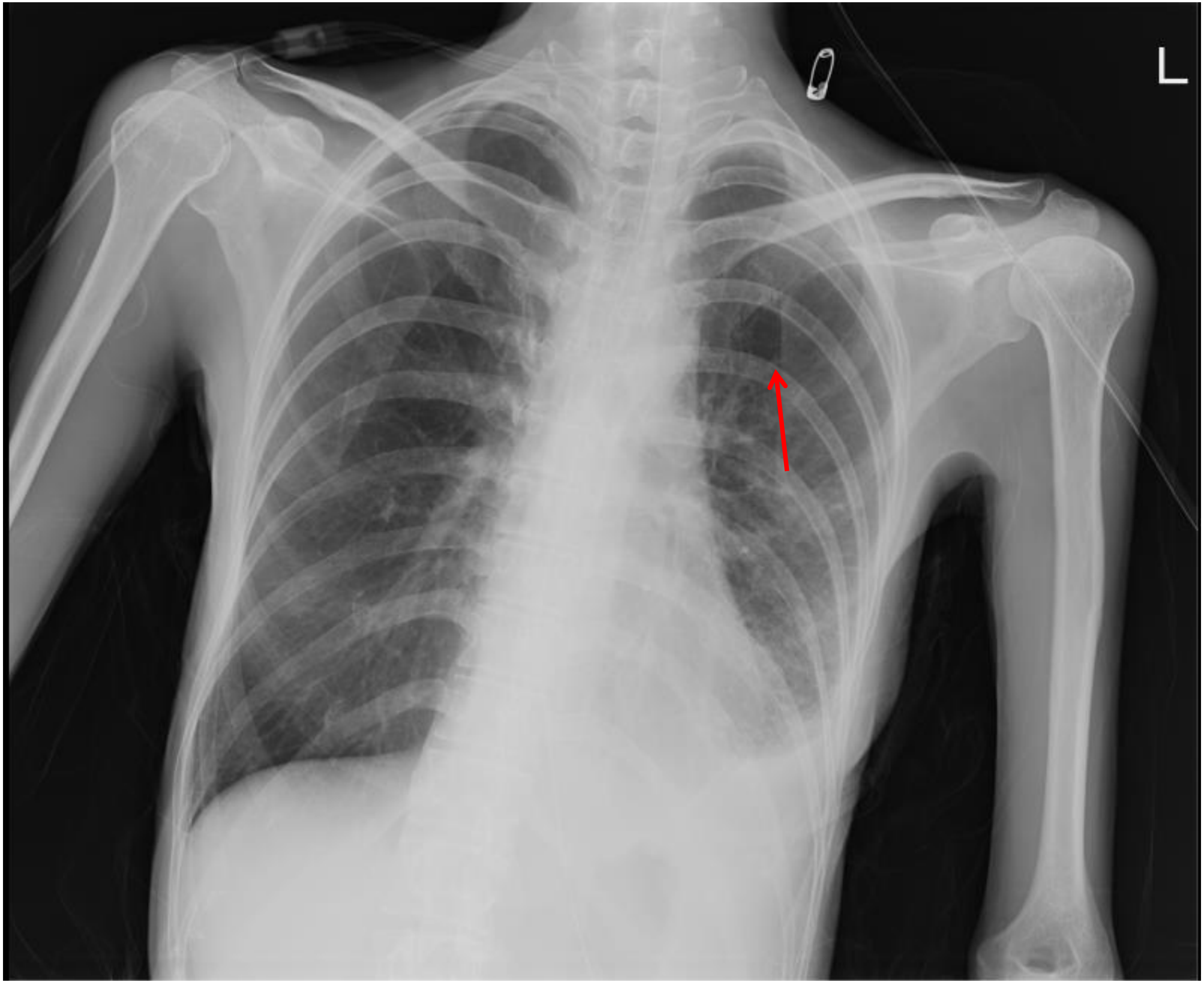

Fig S4b

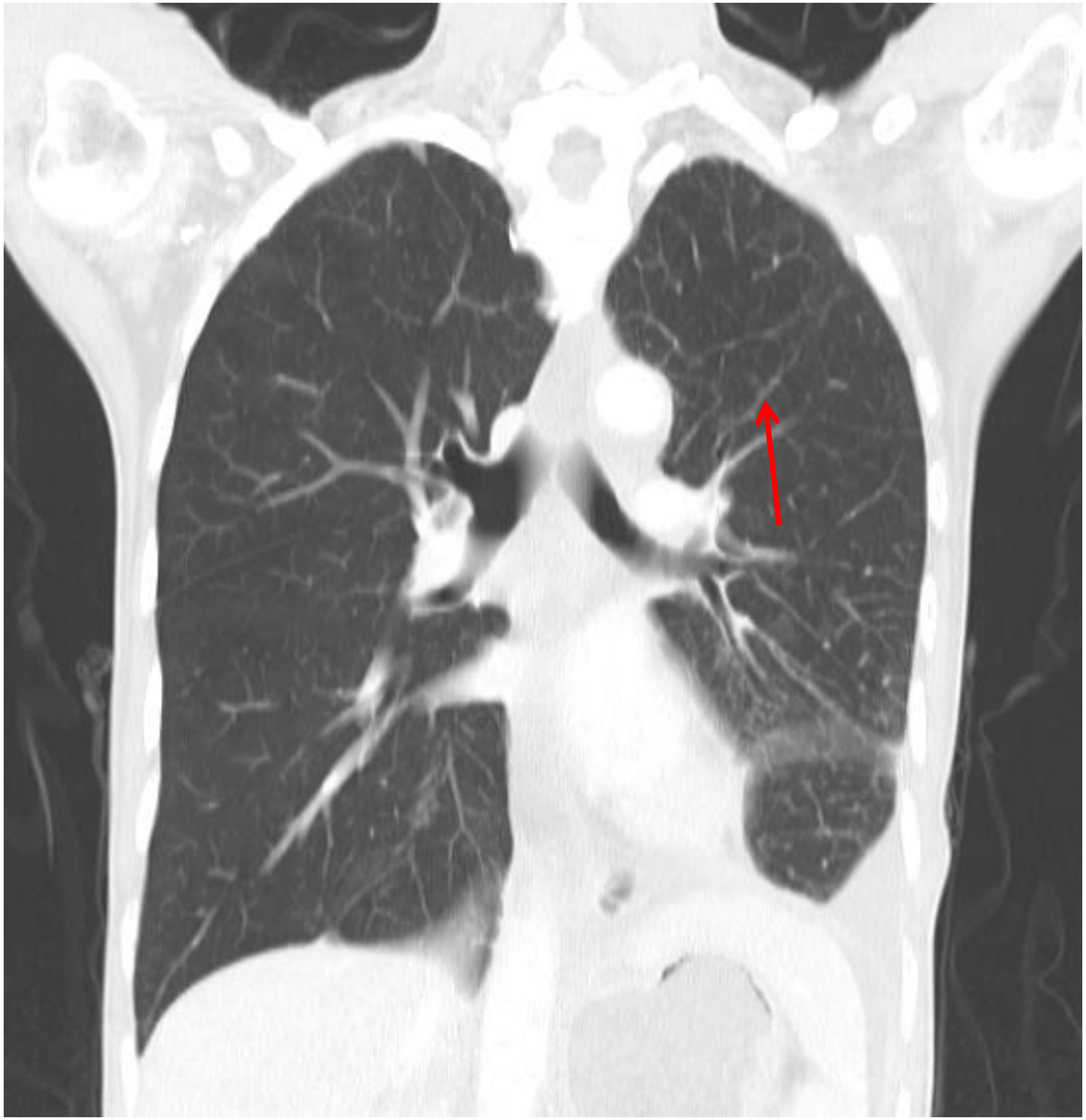

Fig S4c

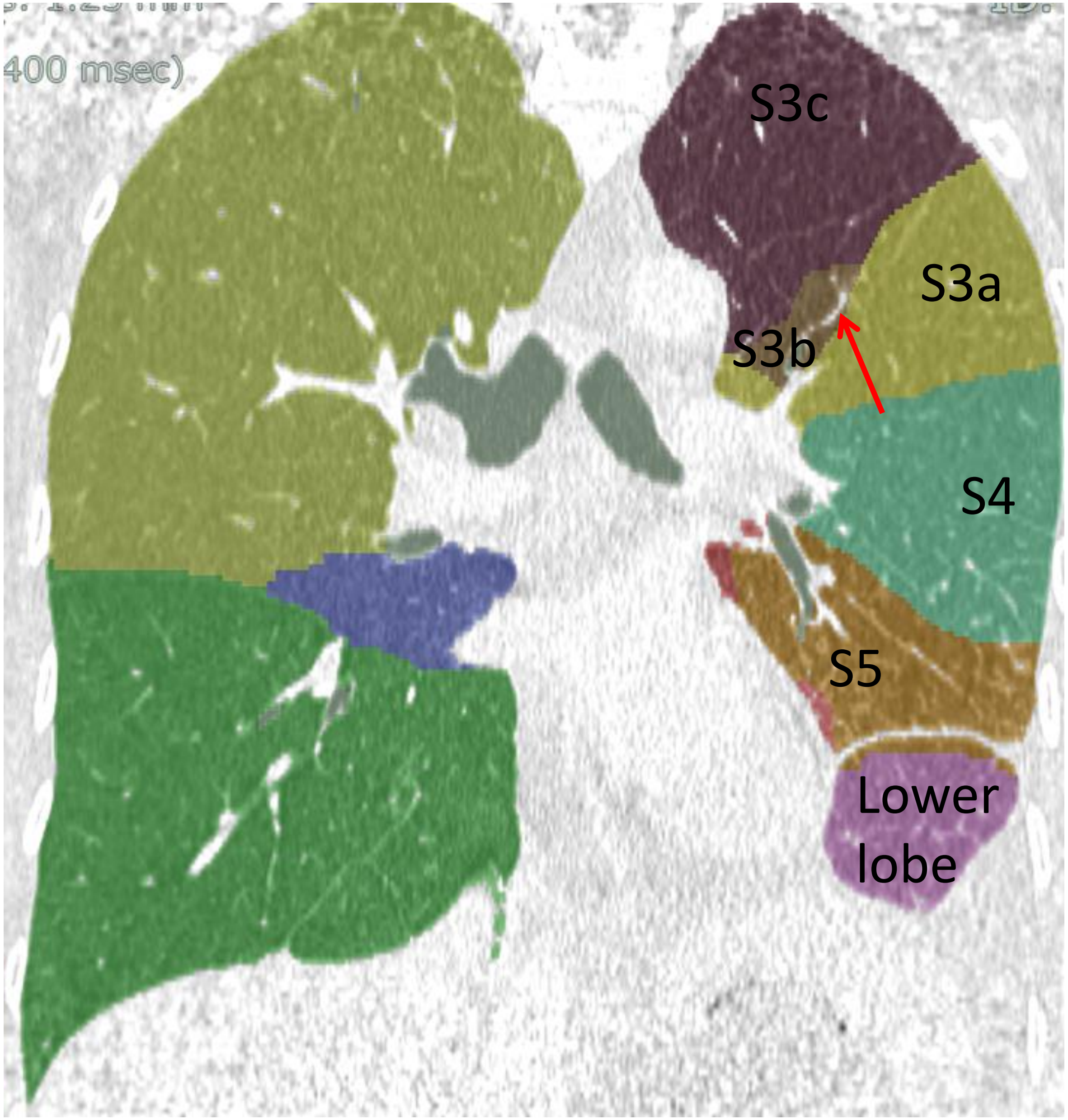

Fig S5a

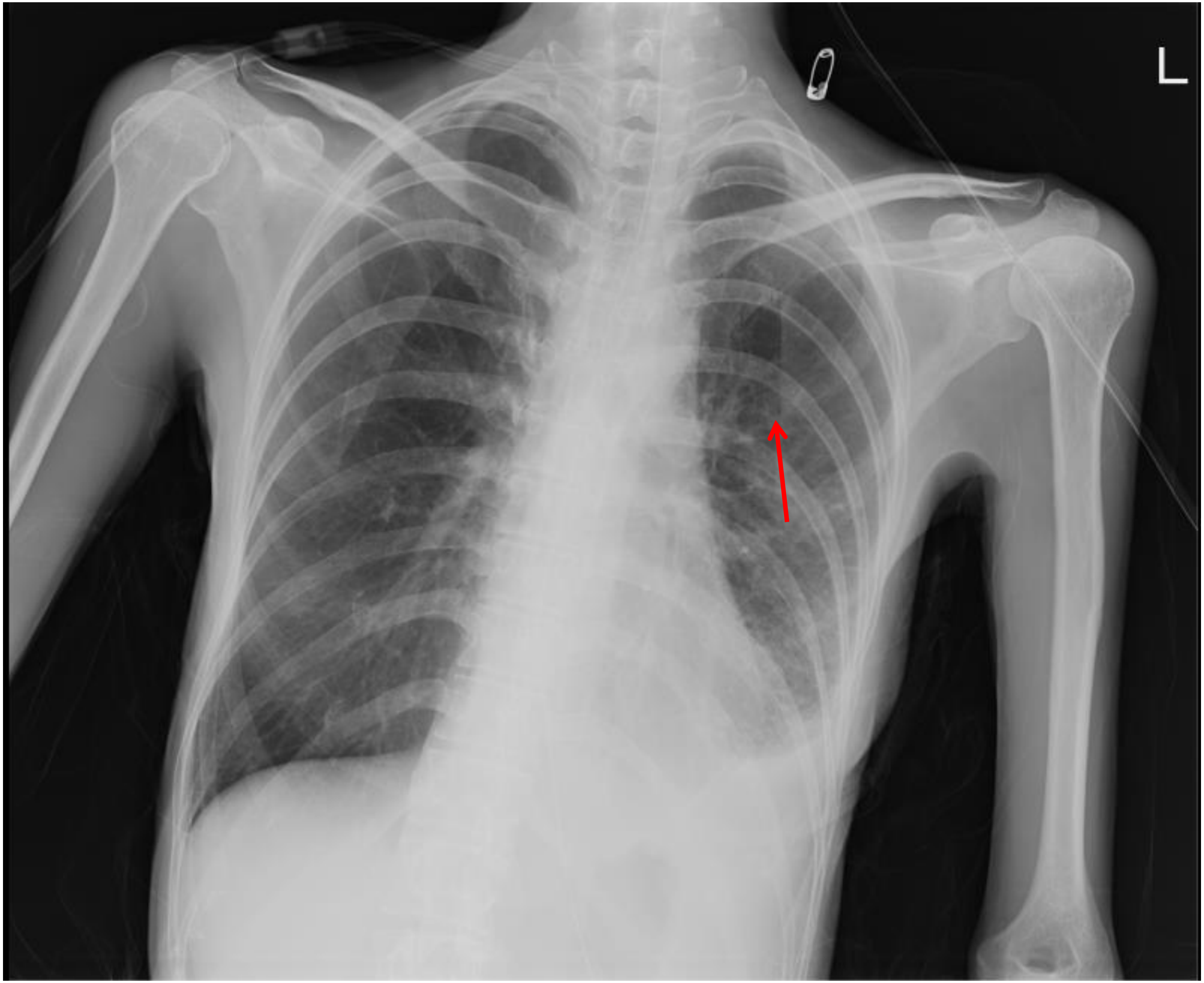

Fig S5b

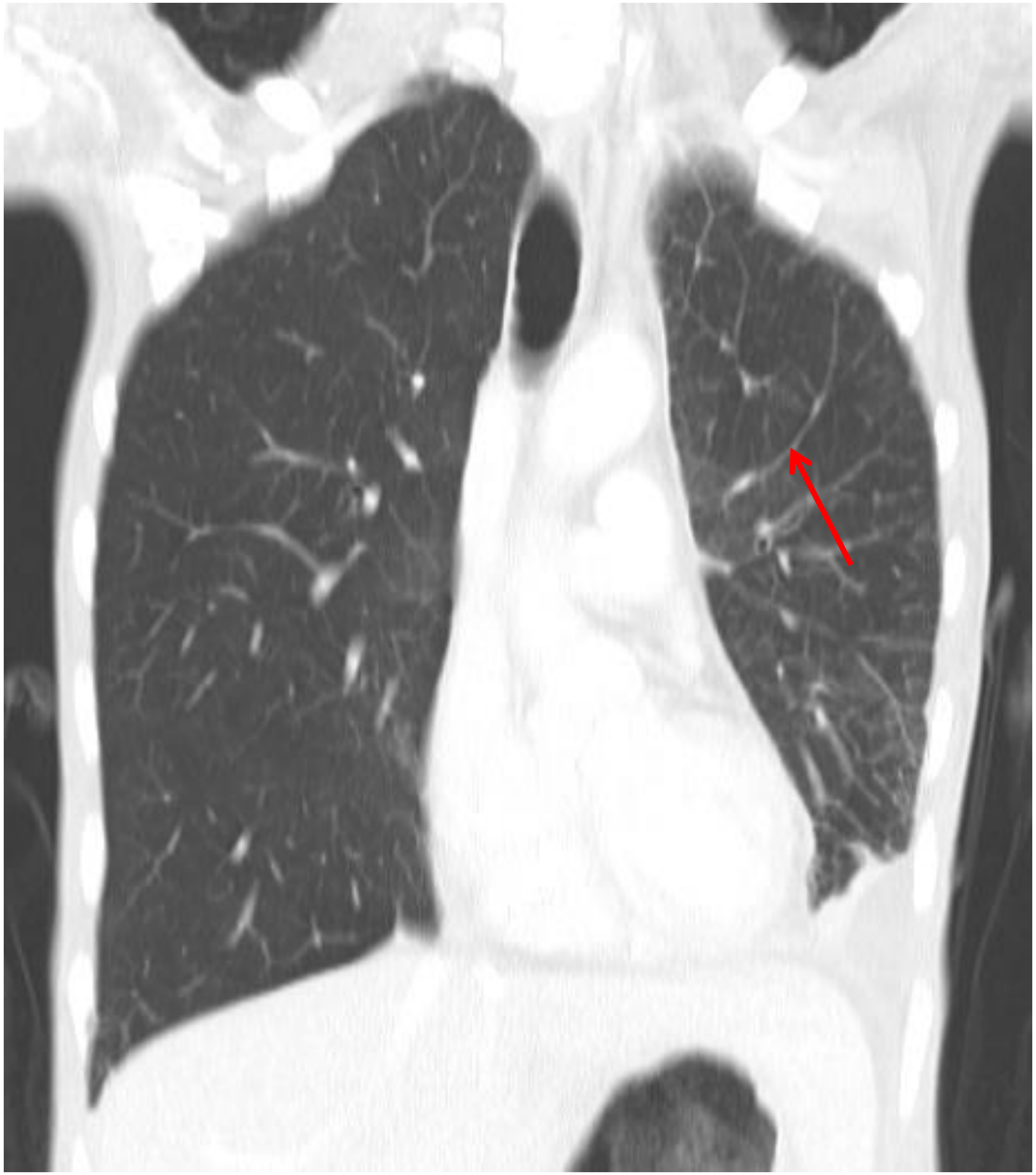

Fig S5c

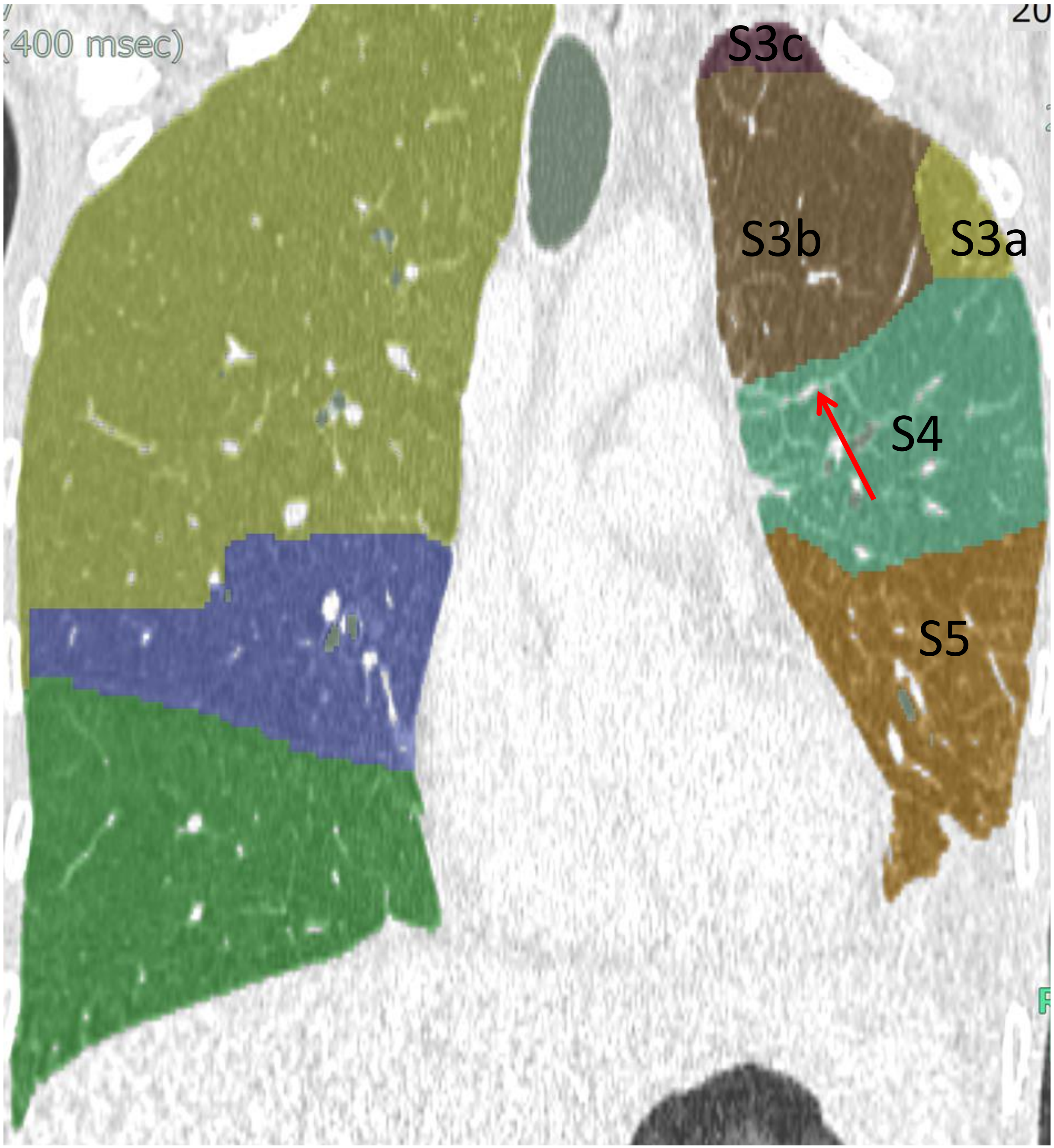

Fig S6a

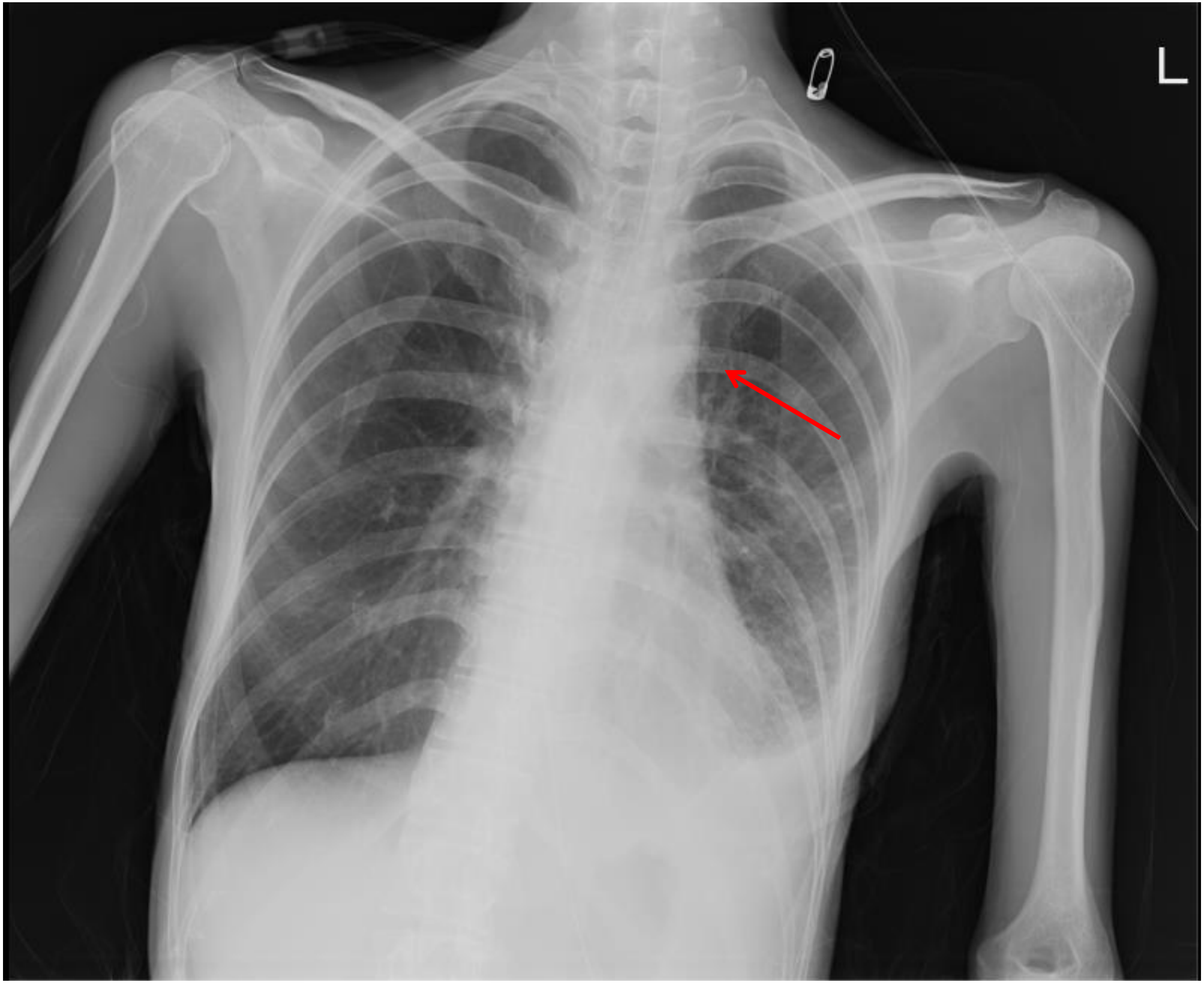

Fig S6b

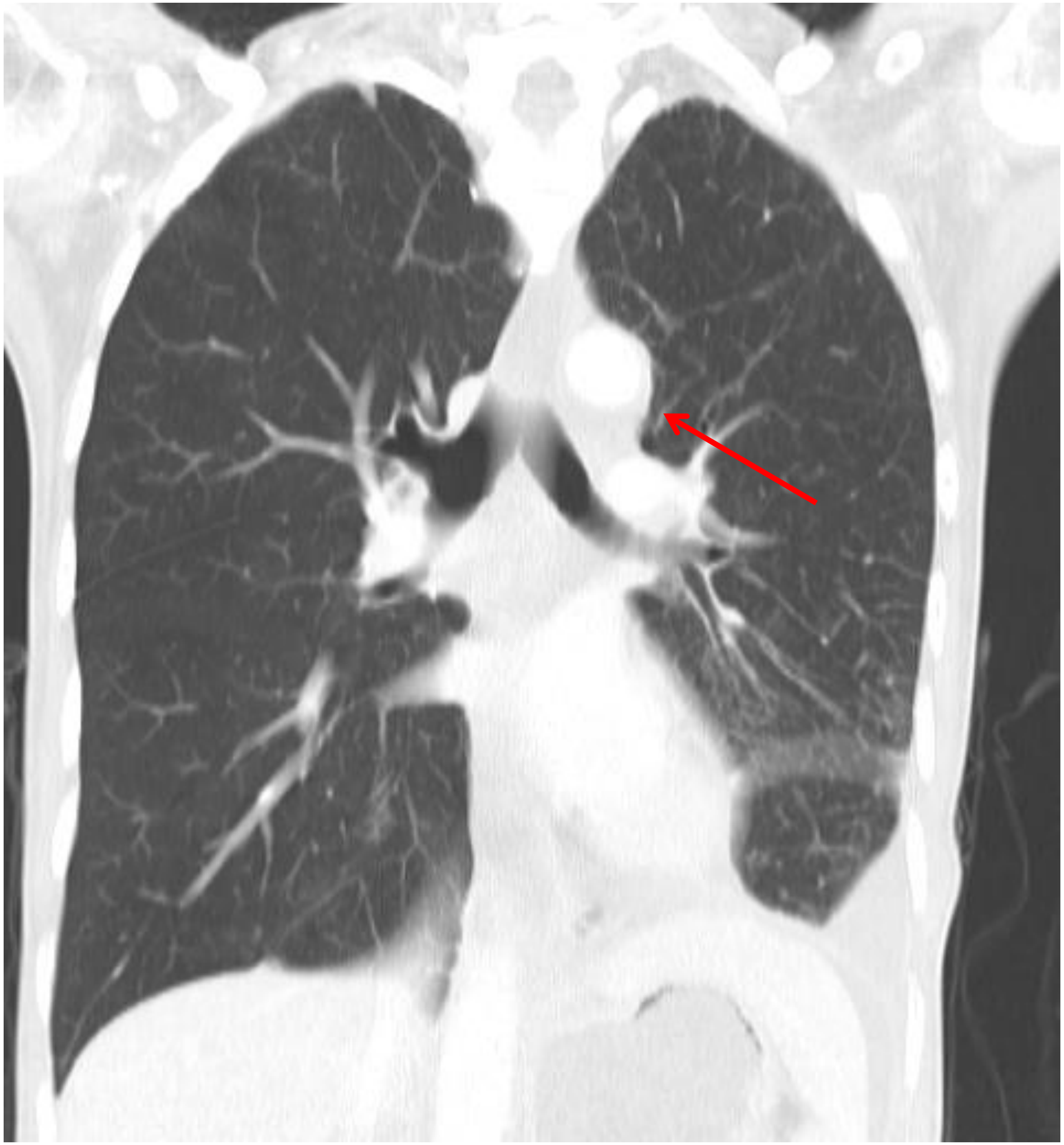

Fig S6c

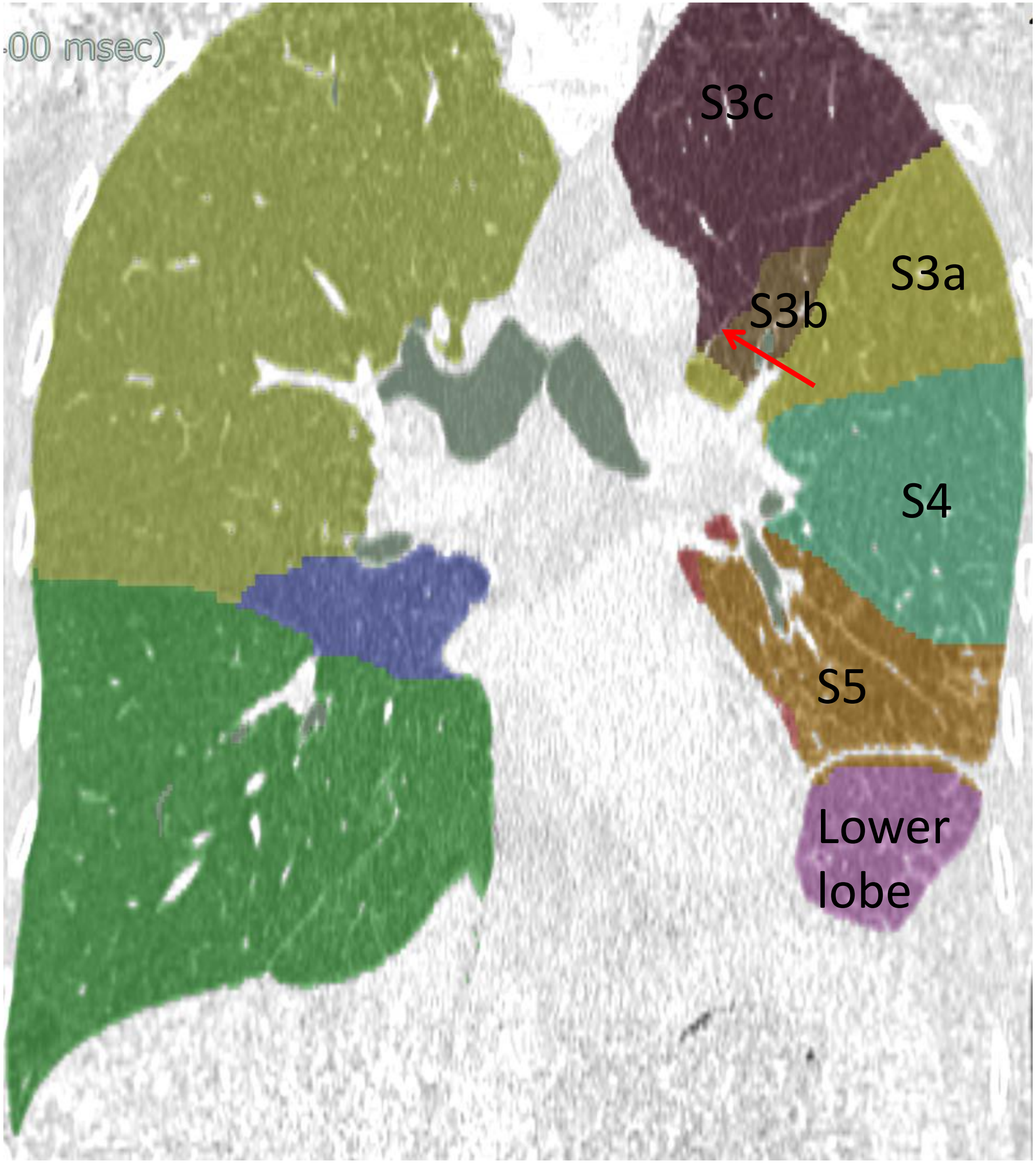

Fig S7a

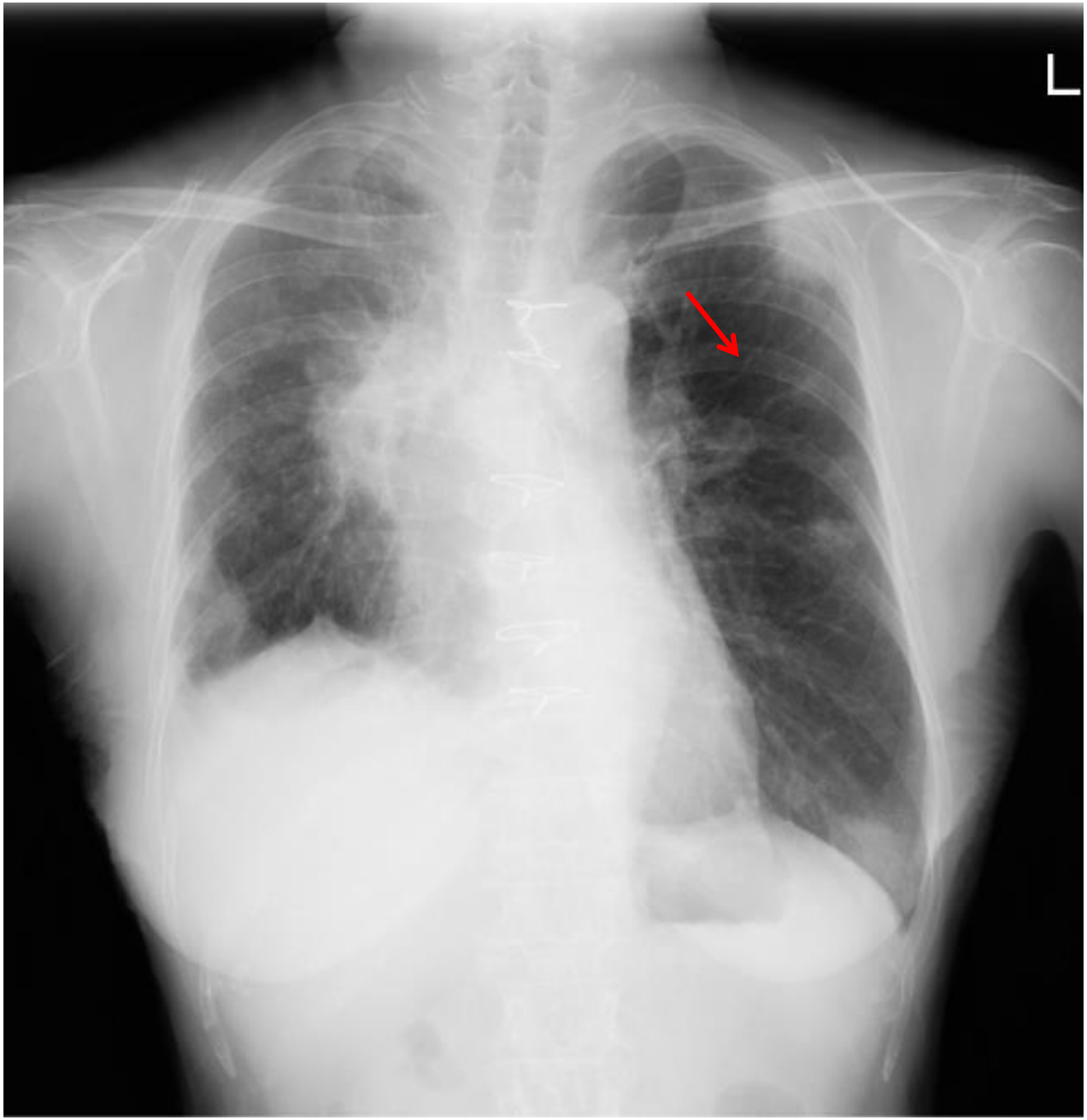

Fig S7b

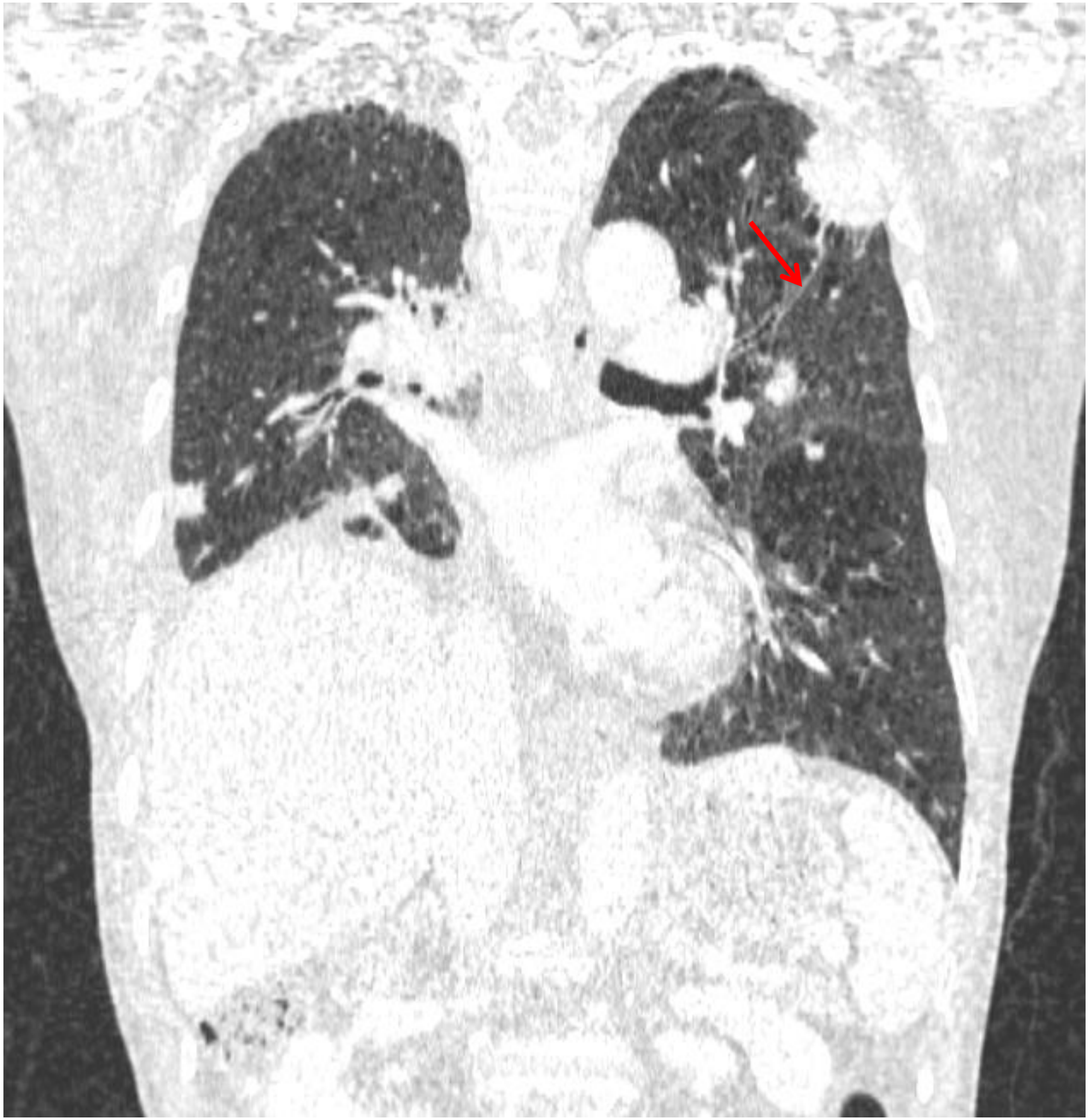

Fig S7c

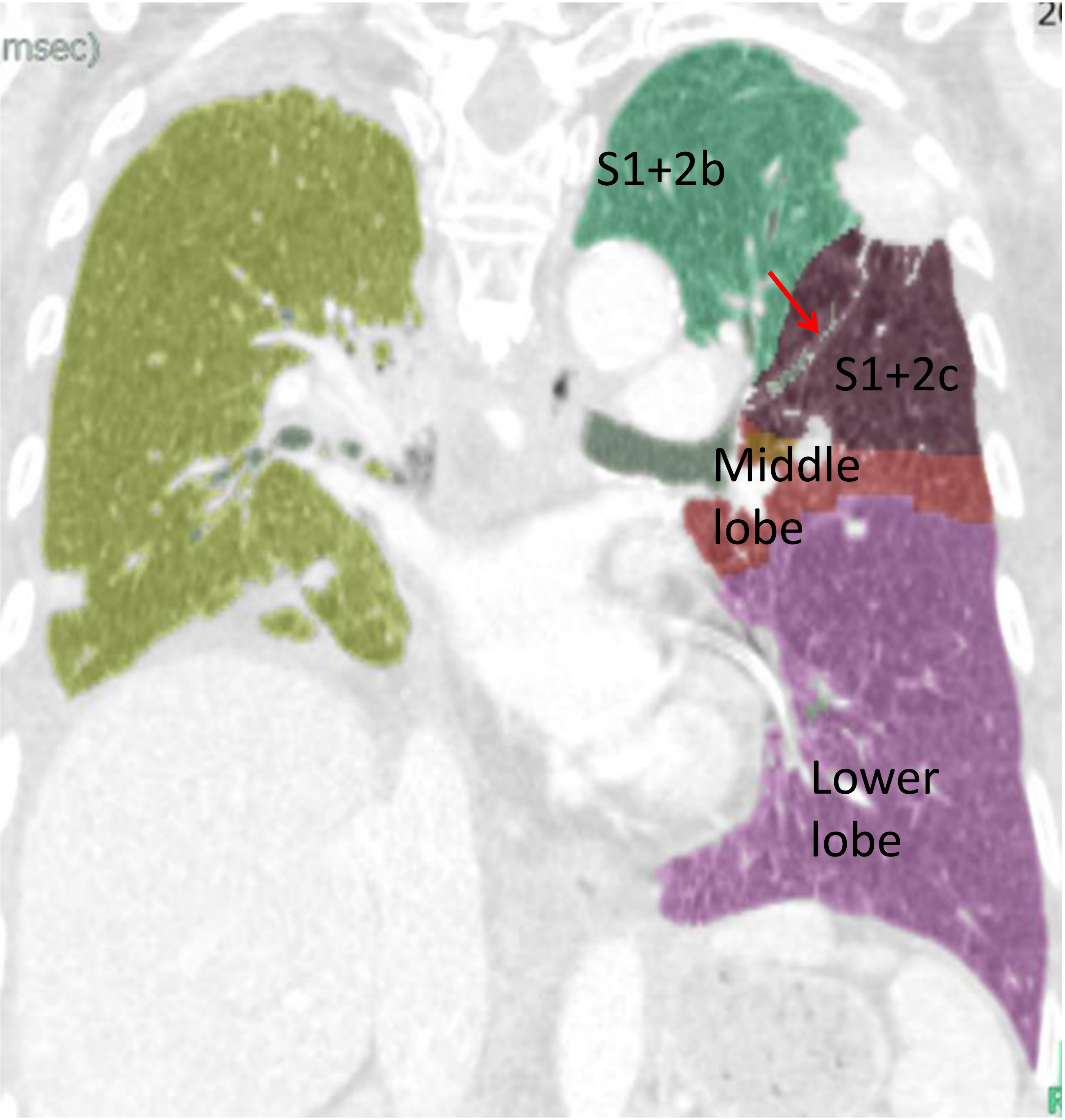

Fig S8a

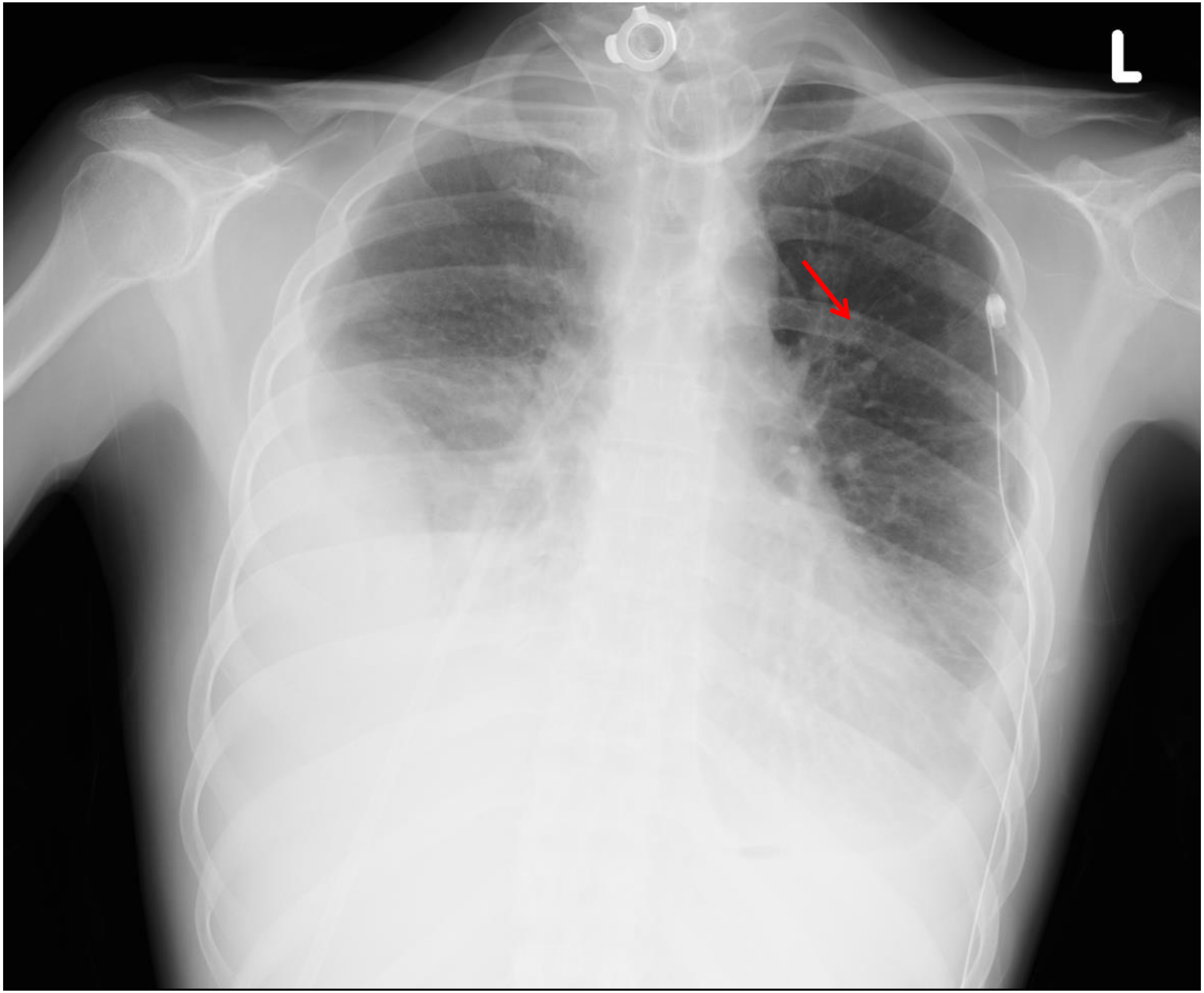

Fig S8b

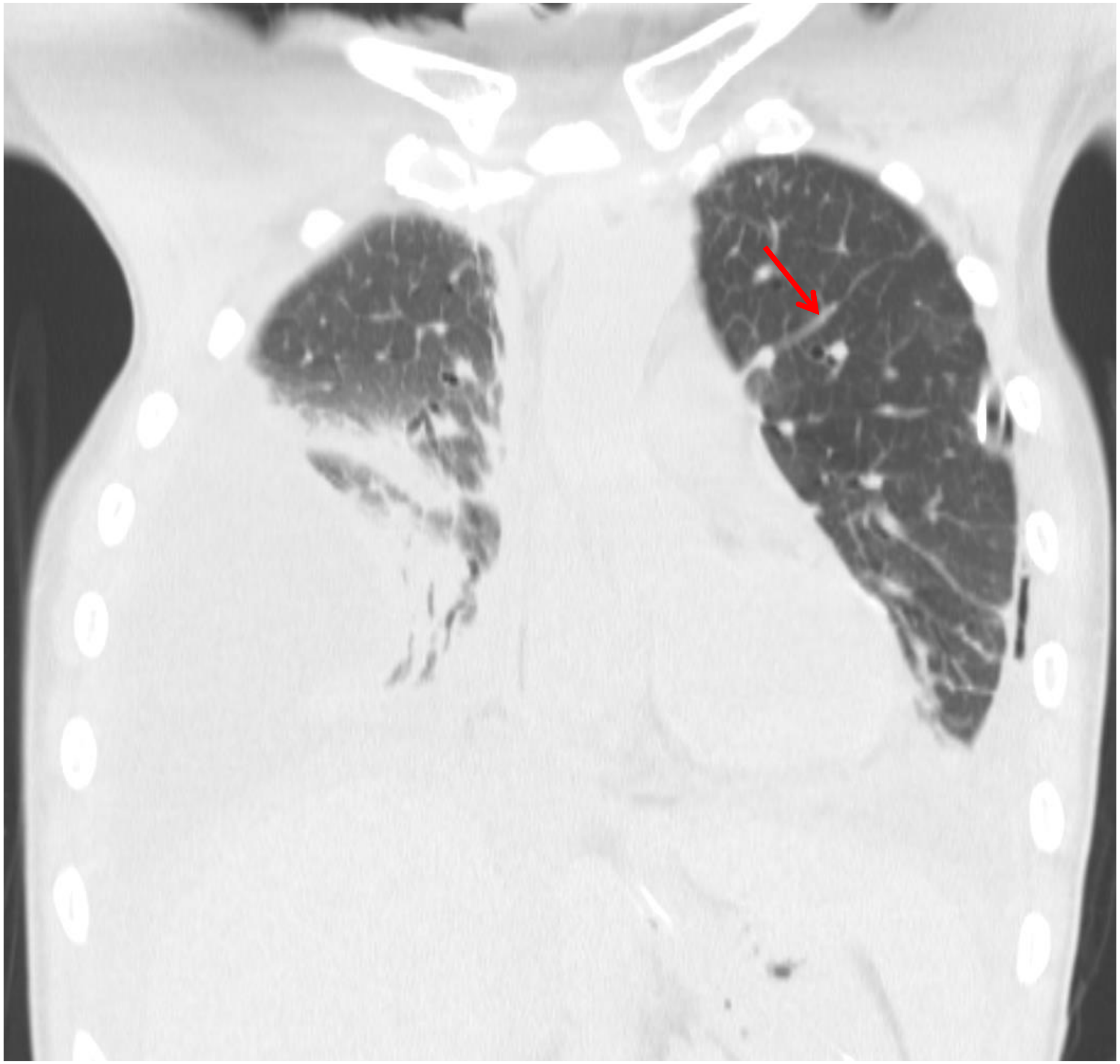

Fig S8c

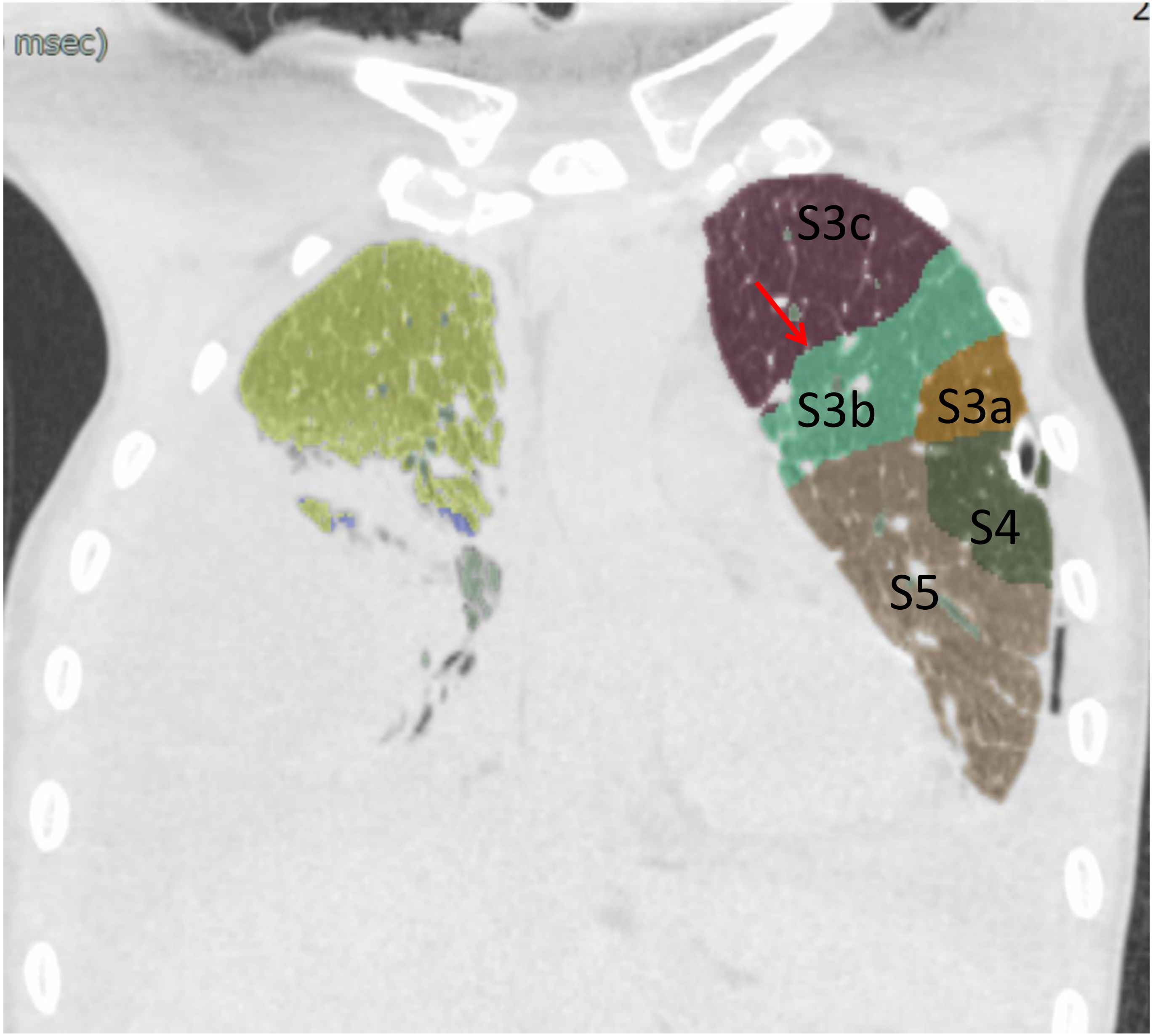

Fig S9a

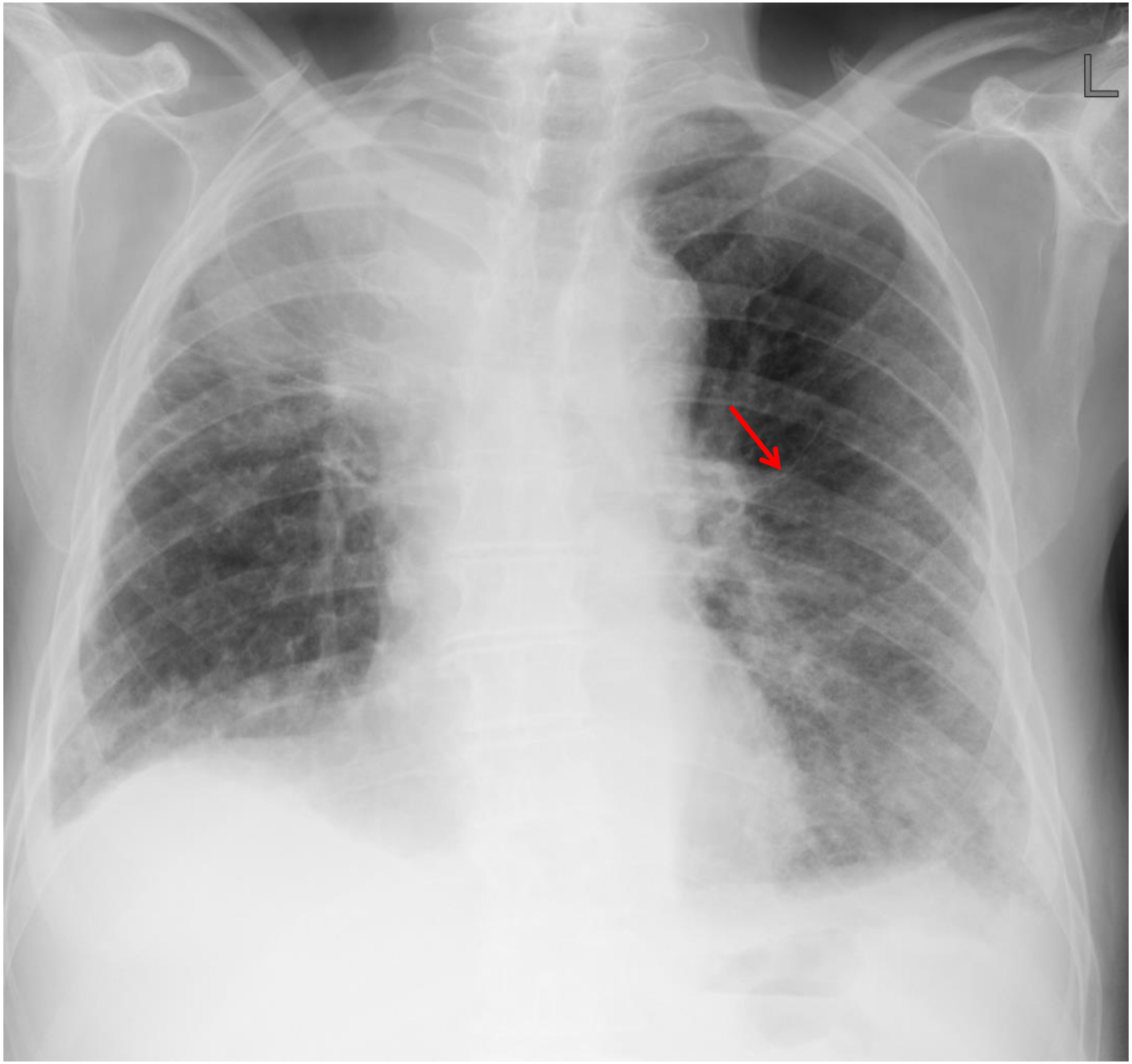

Fig S9b

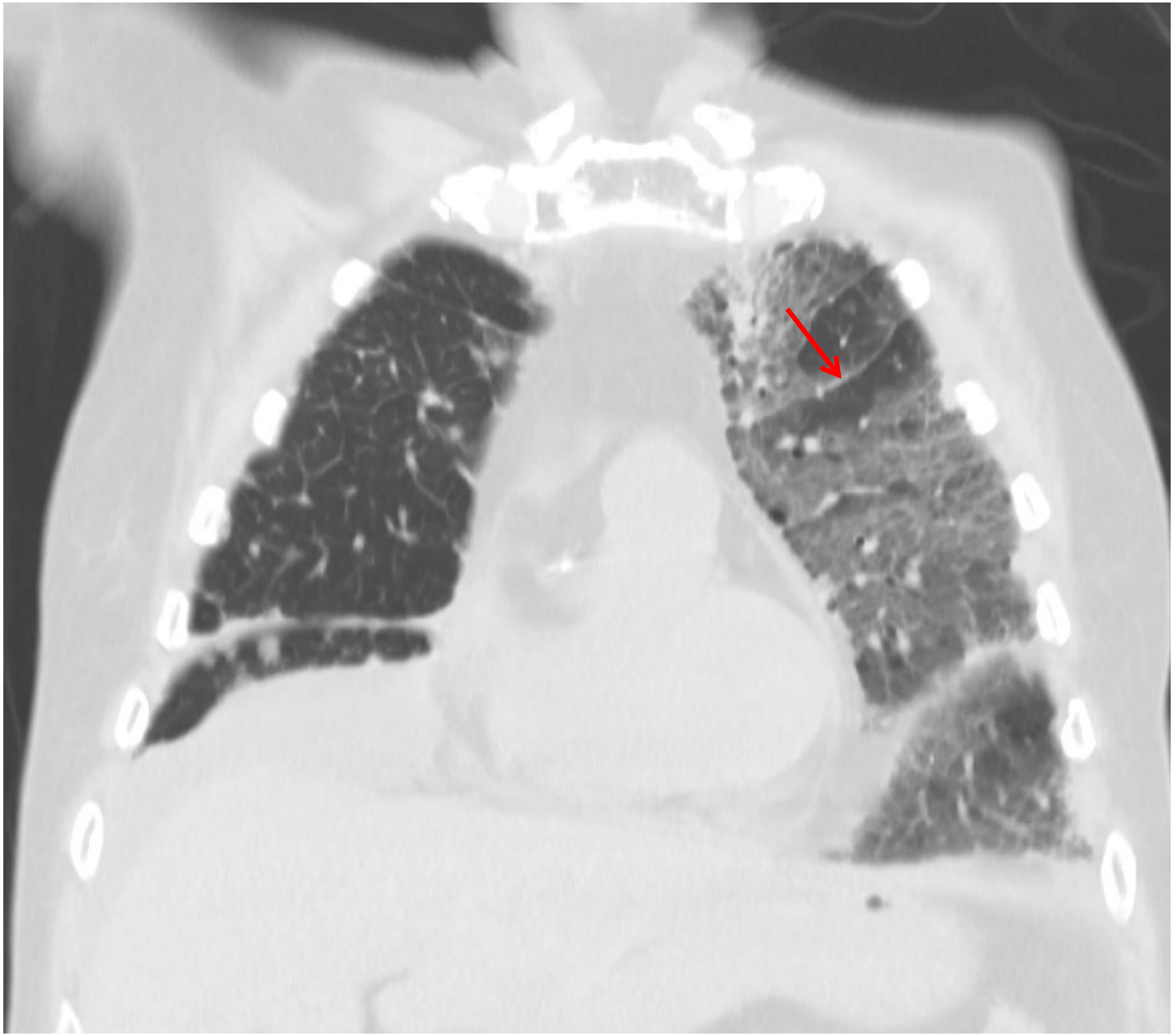

Fig S9c

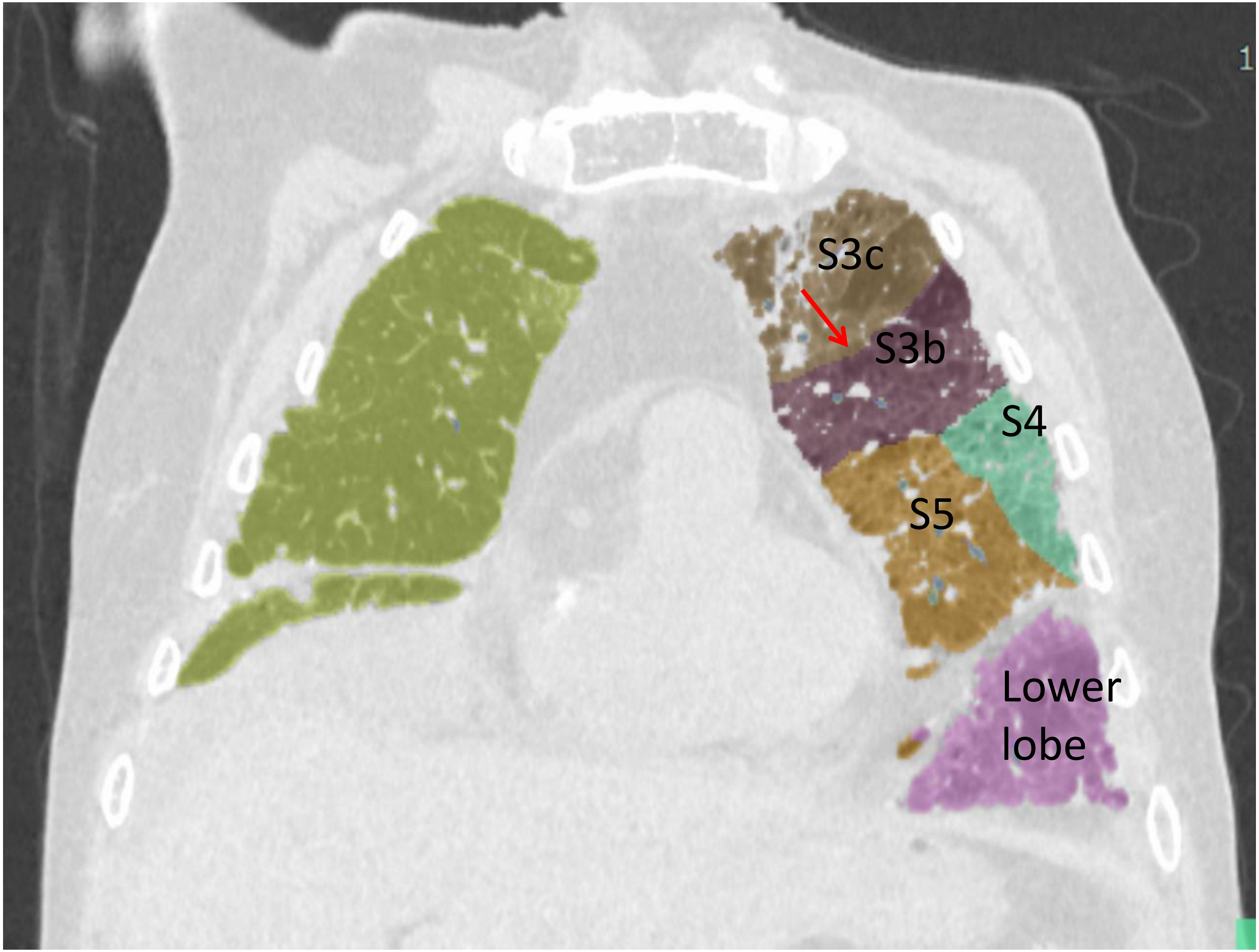

Fig S10a

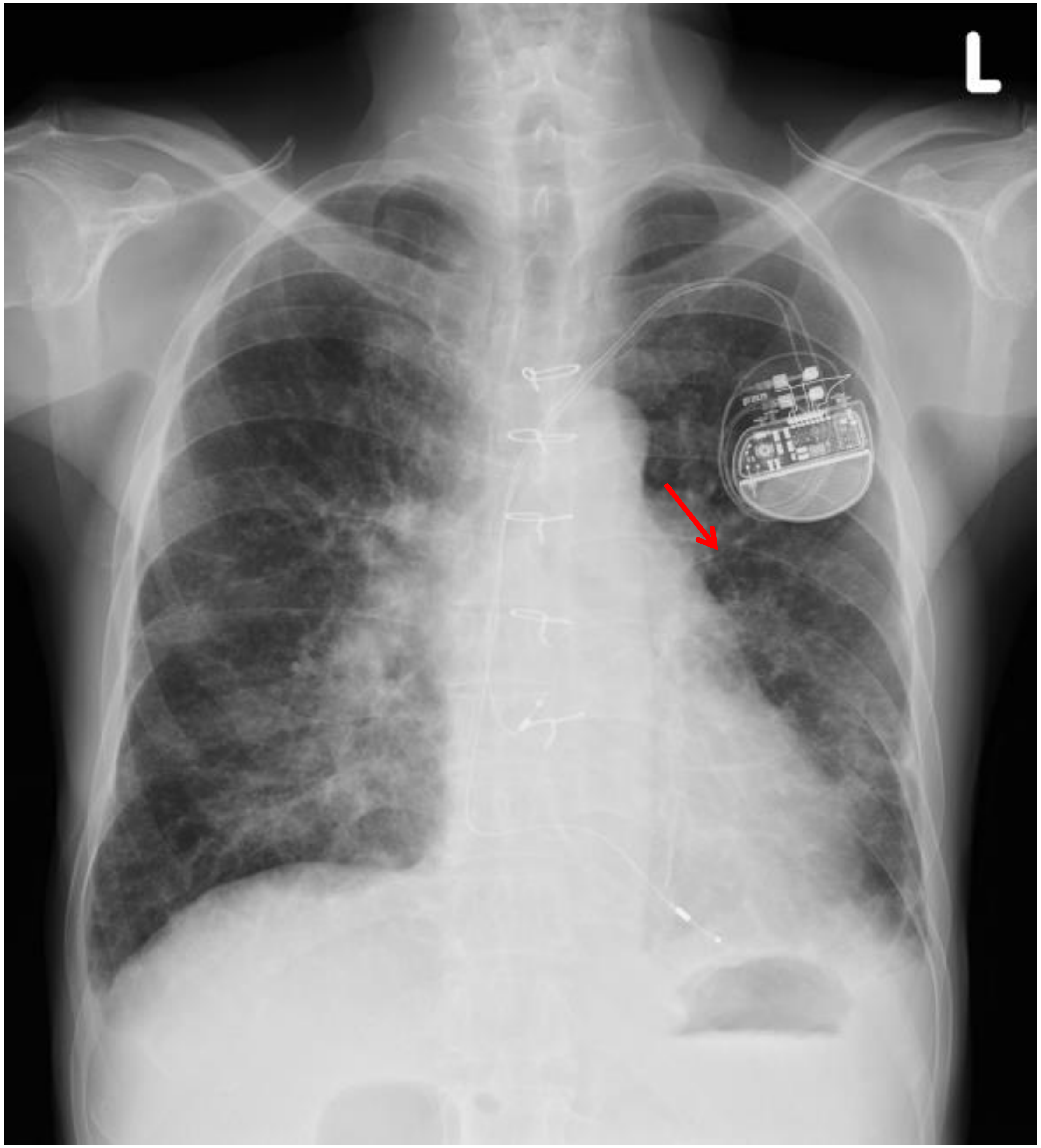

Fig S10b

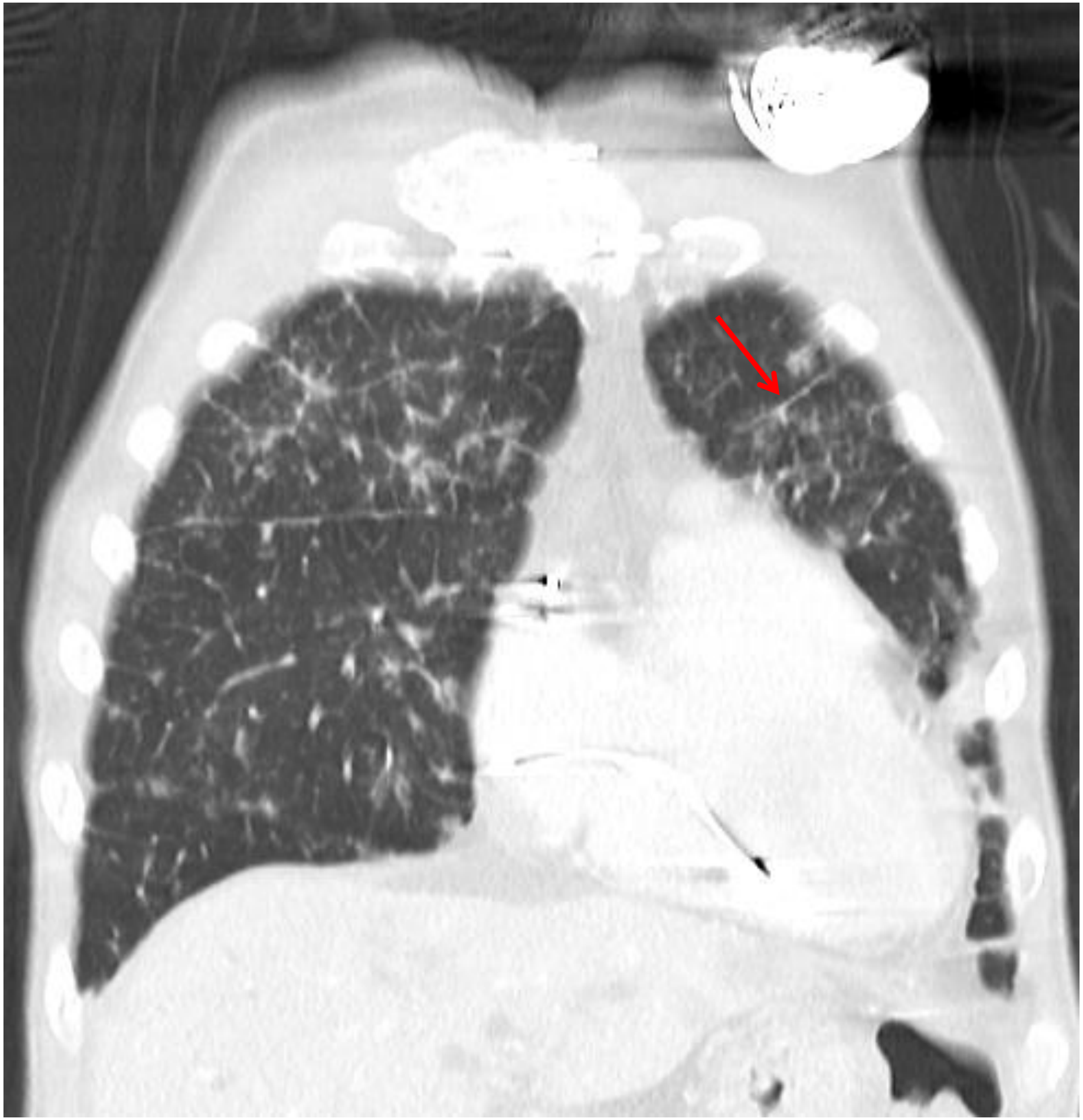

Fig S10c

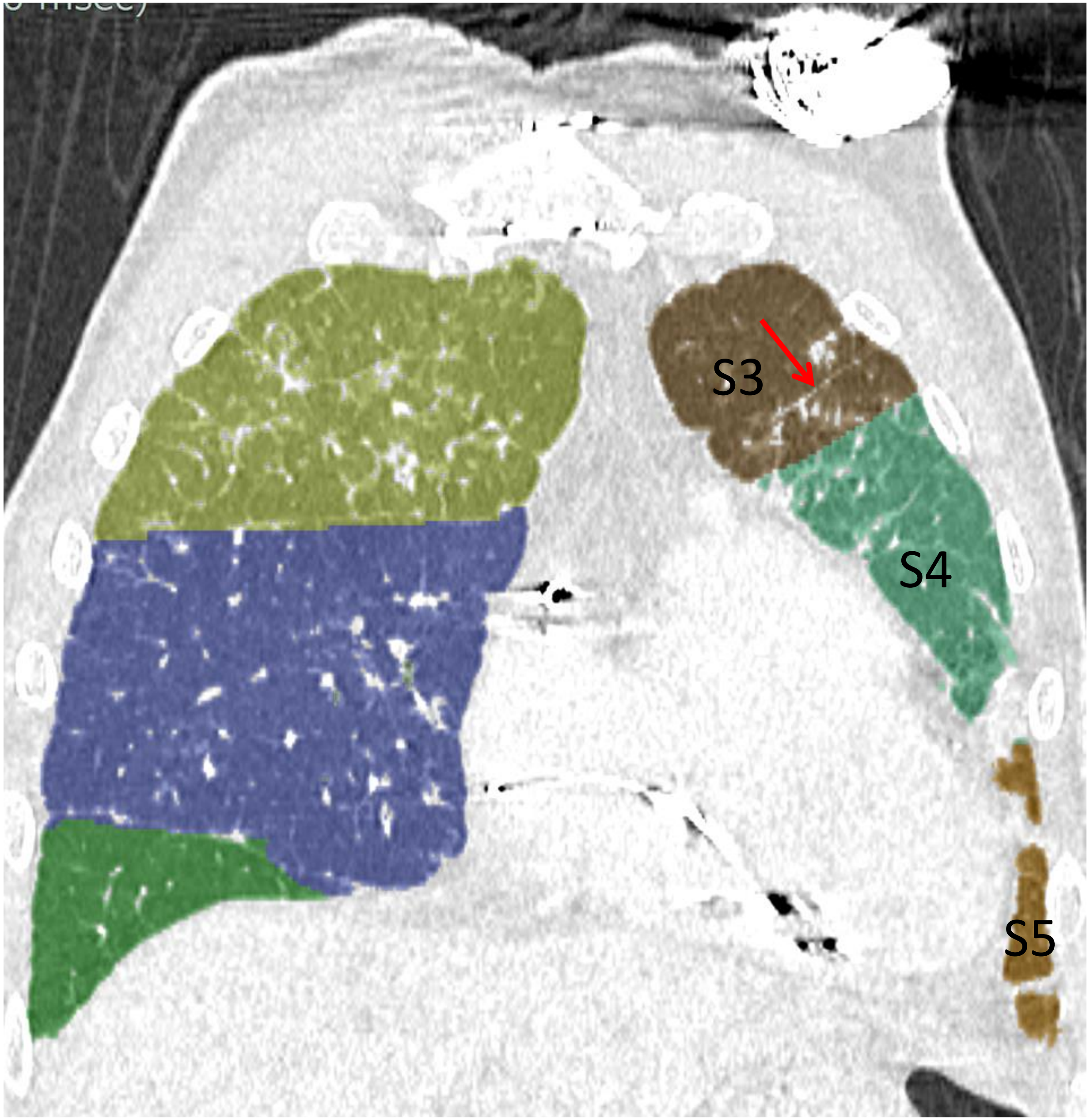

Fig S11a

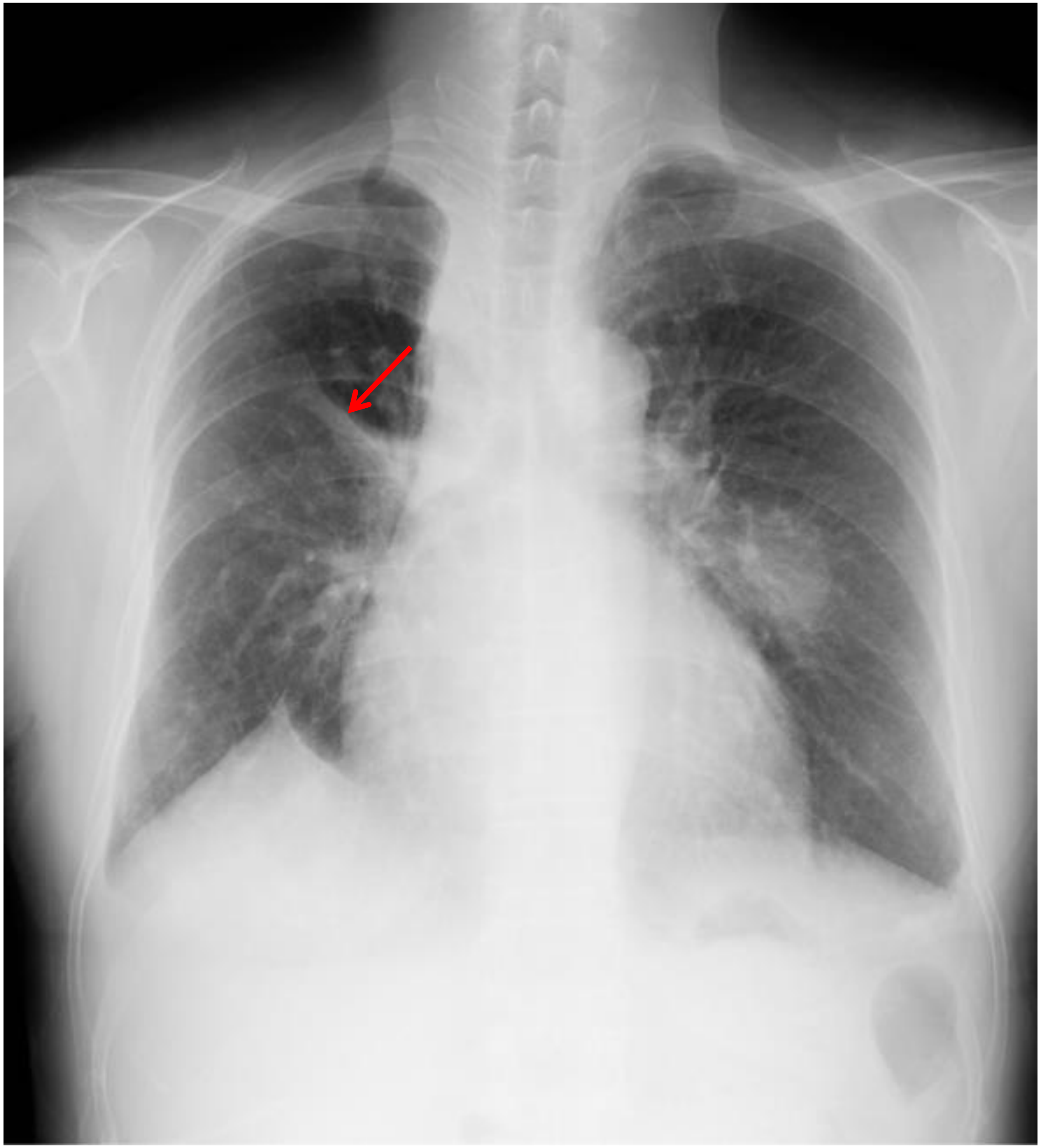

Fig S11b

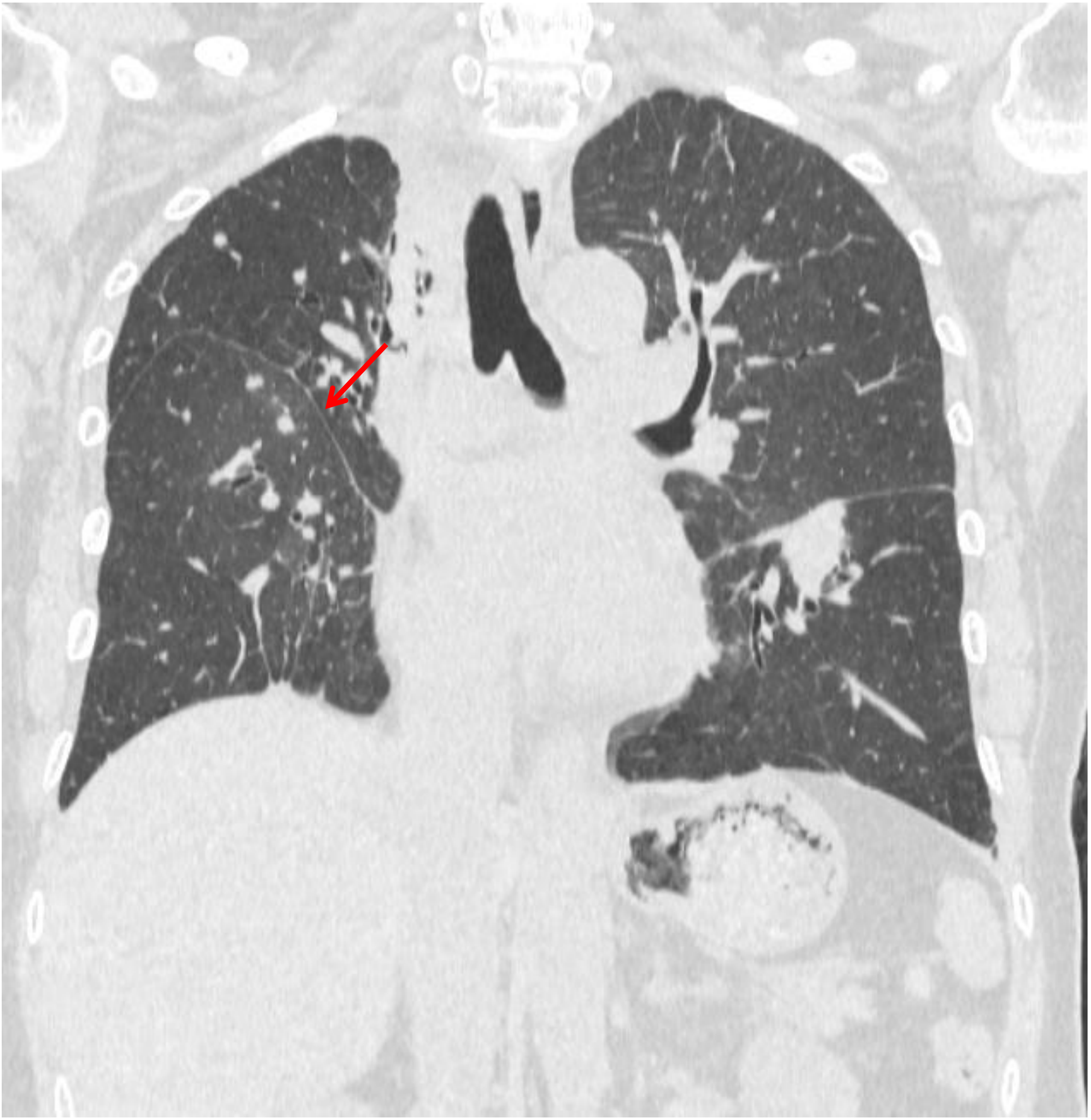

Fig S11c

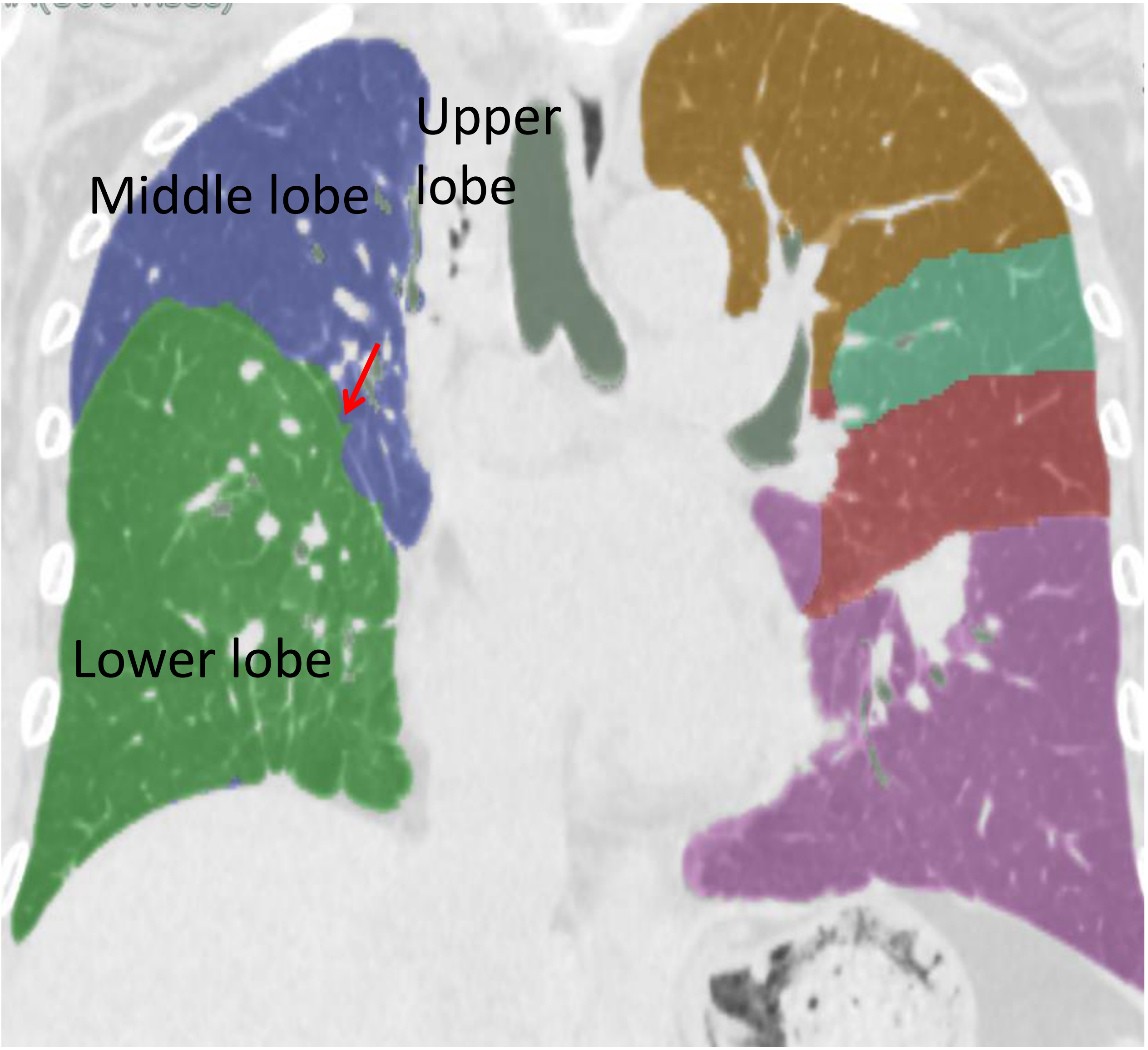

Fig S12a

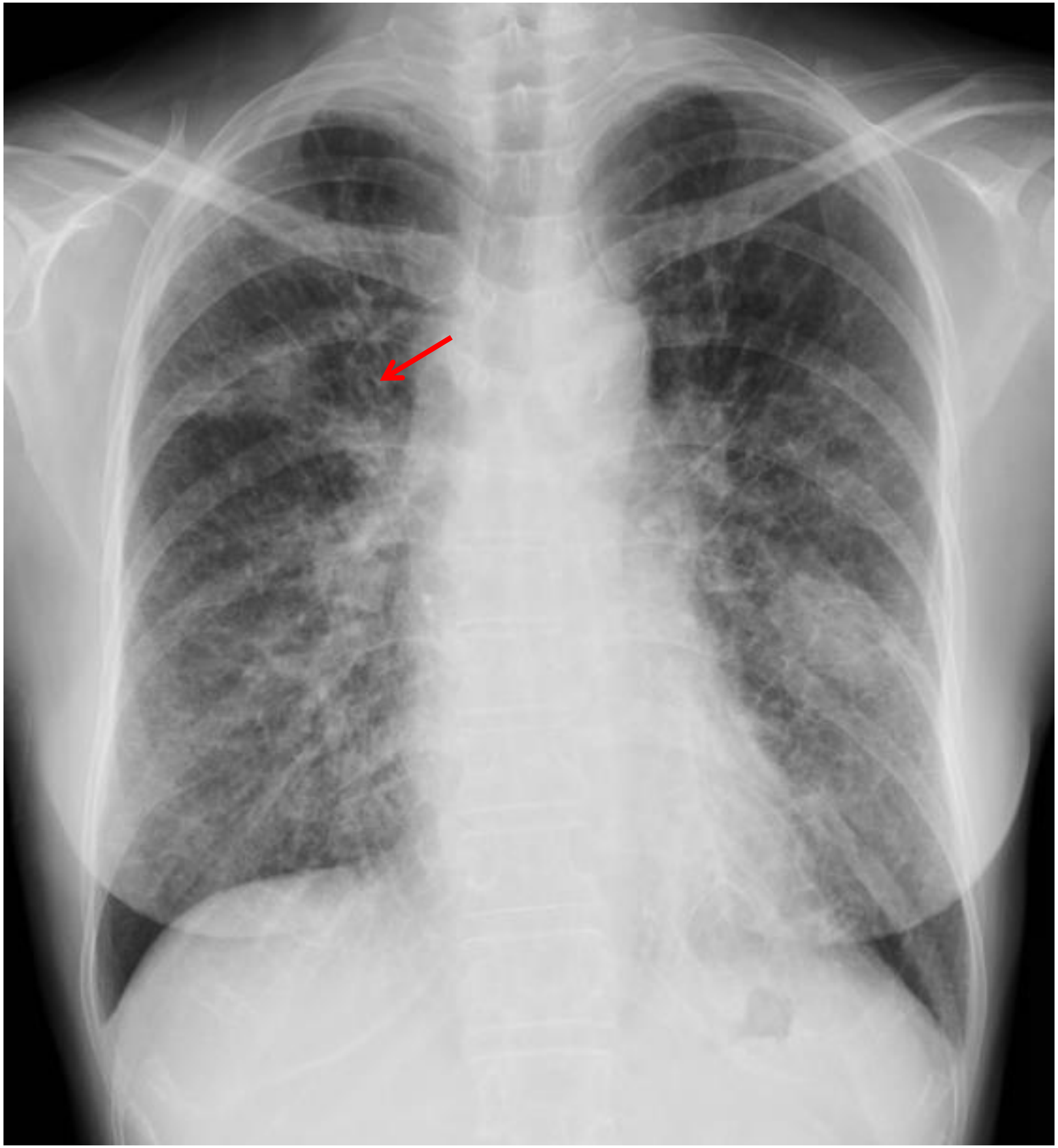

Fig S12b

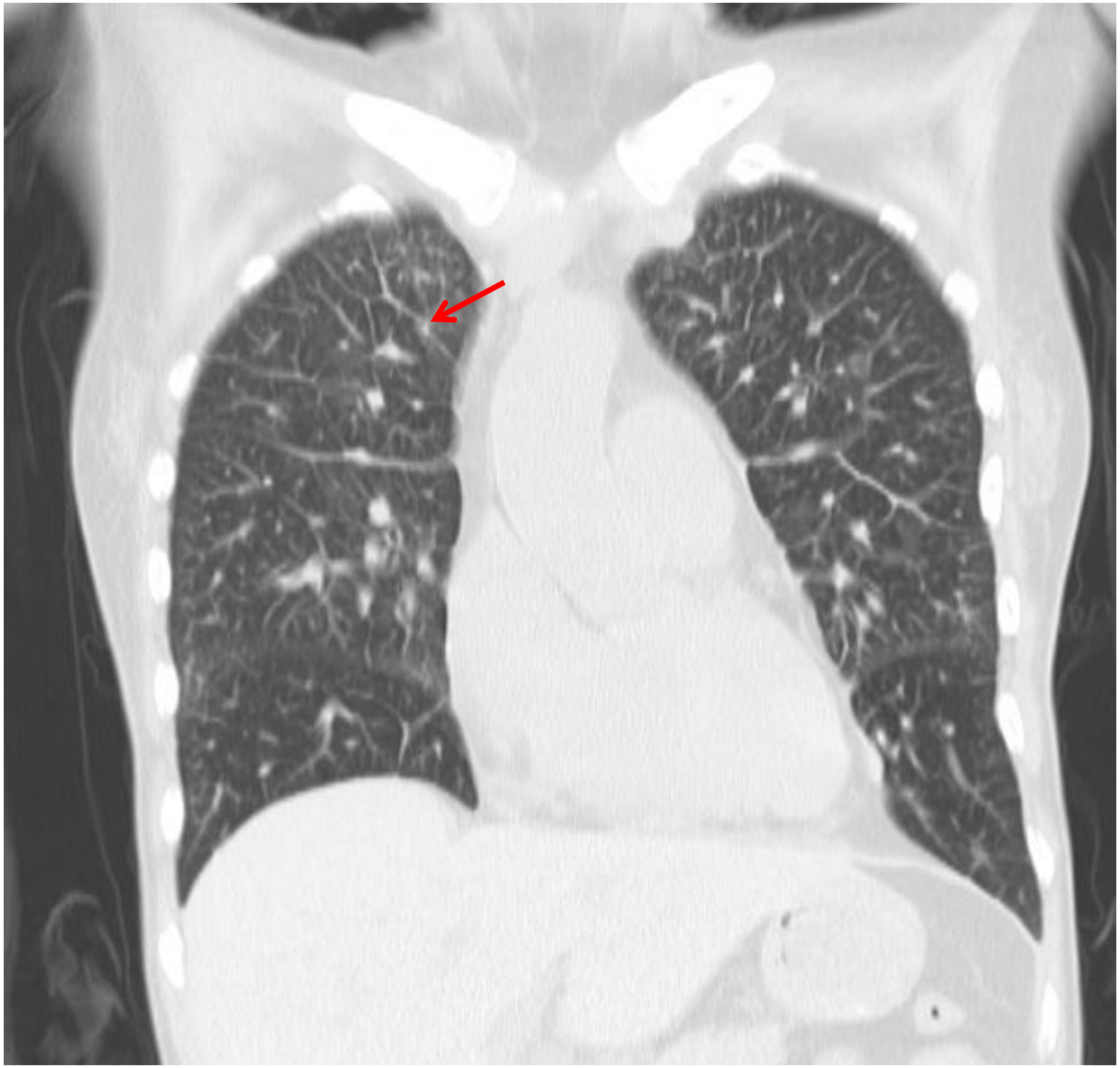

Fig S12c

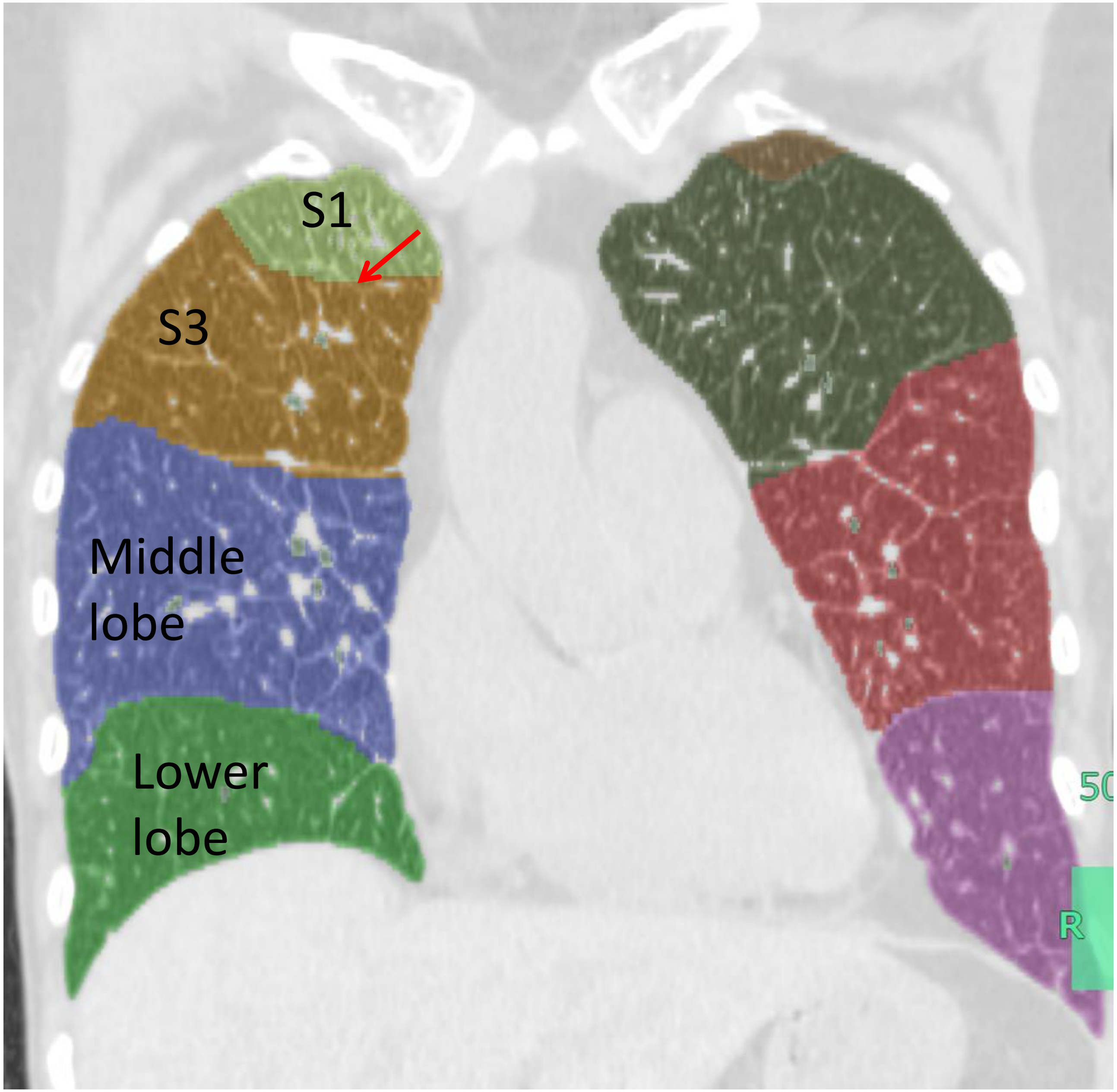

Fig S13a

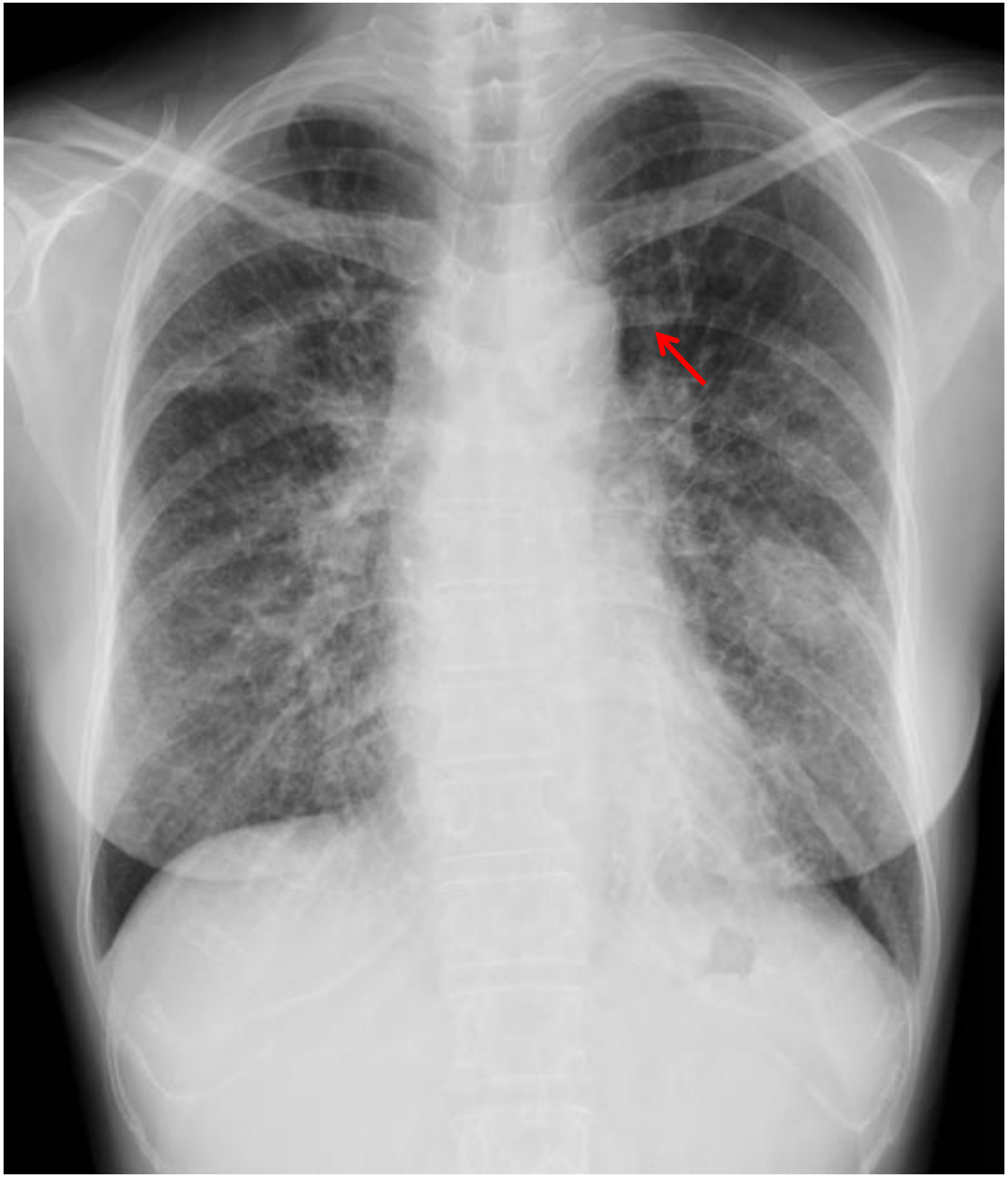

Fig S13b

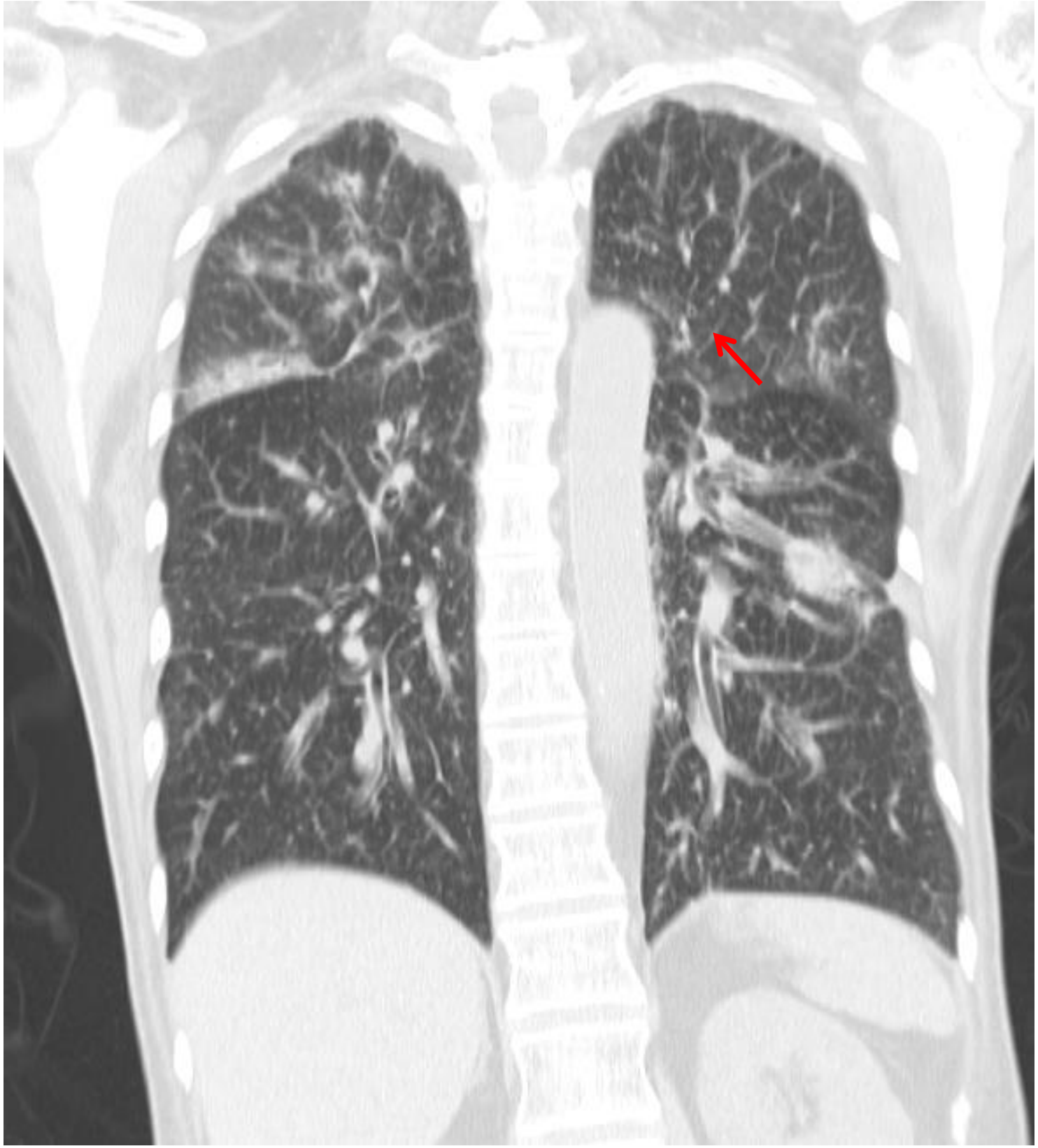

Fig S13c

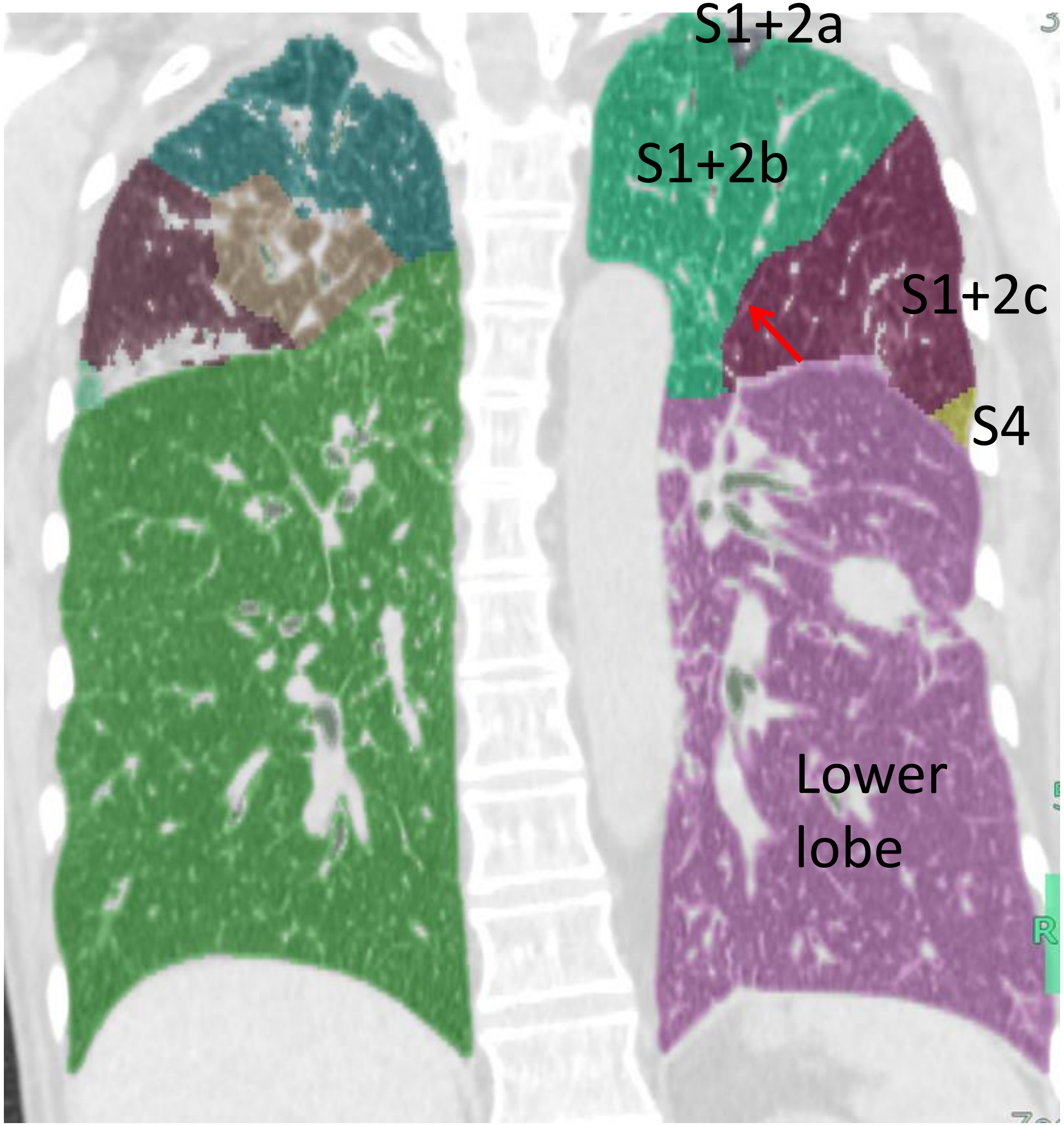

Fig S14a

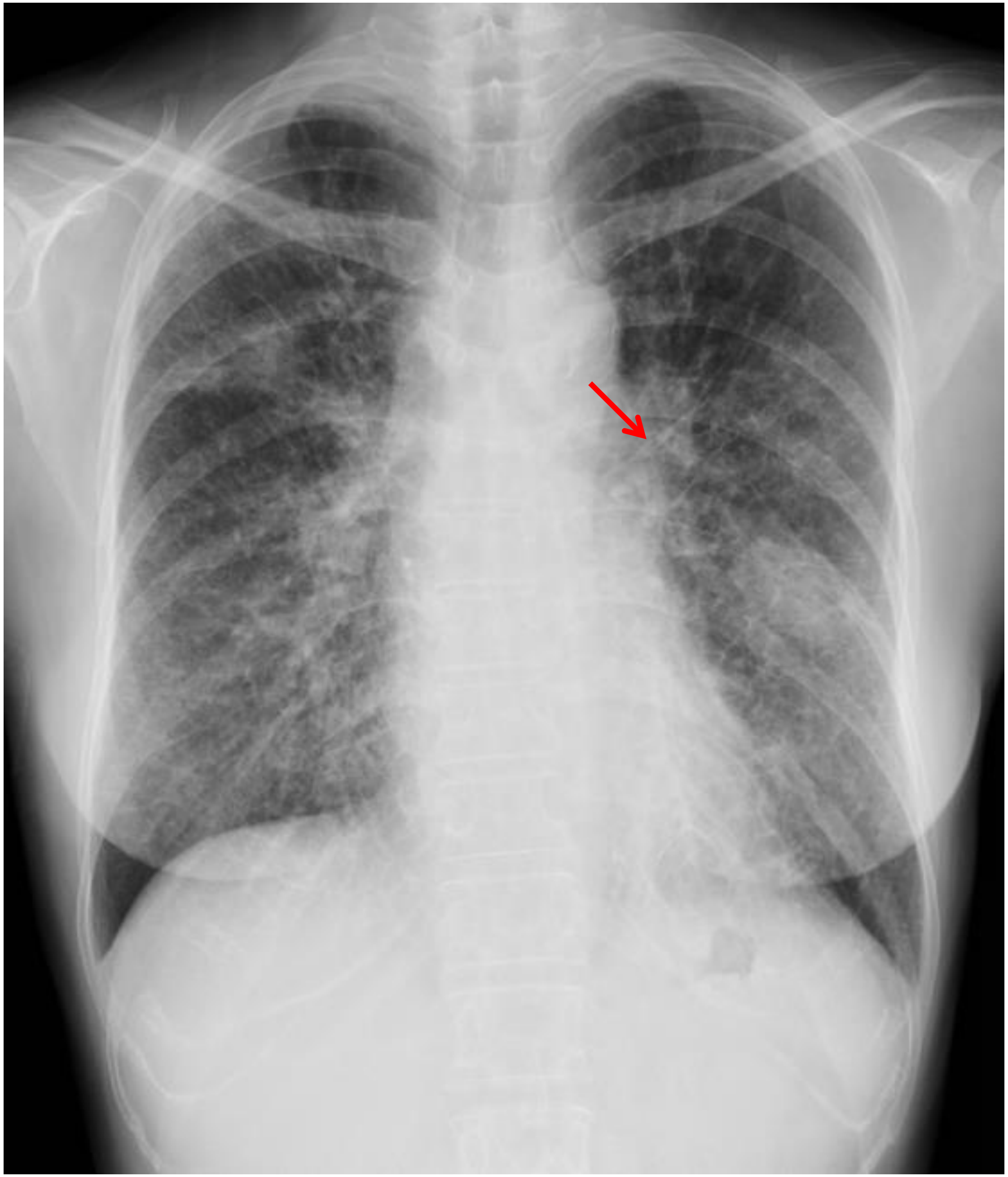

Fig S14b

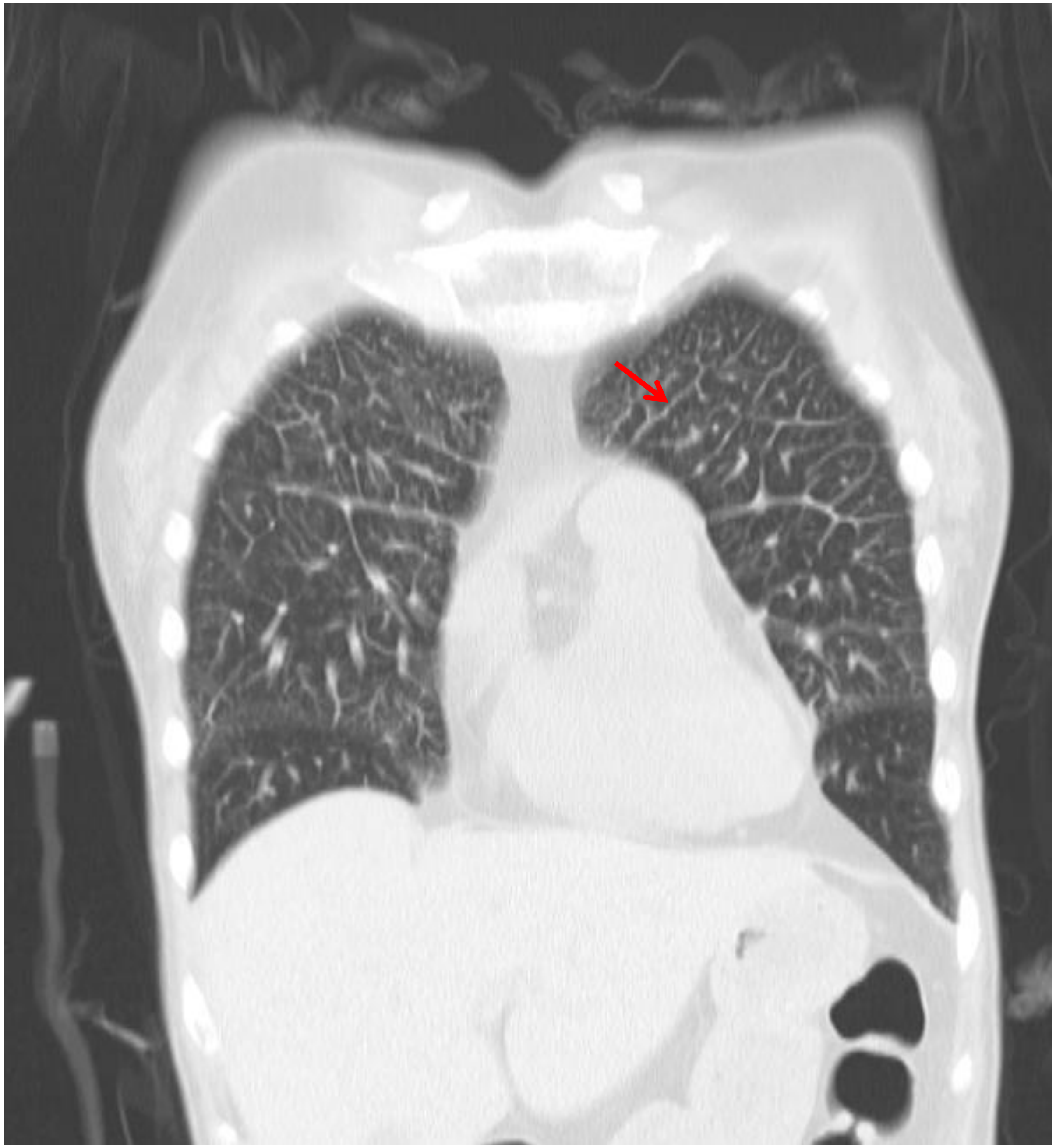

Fig S14c

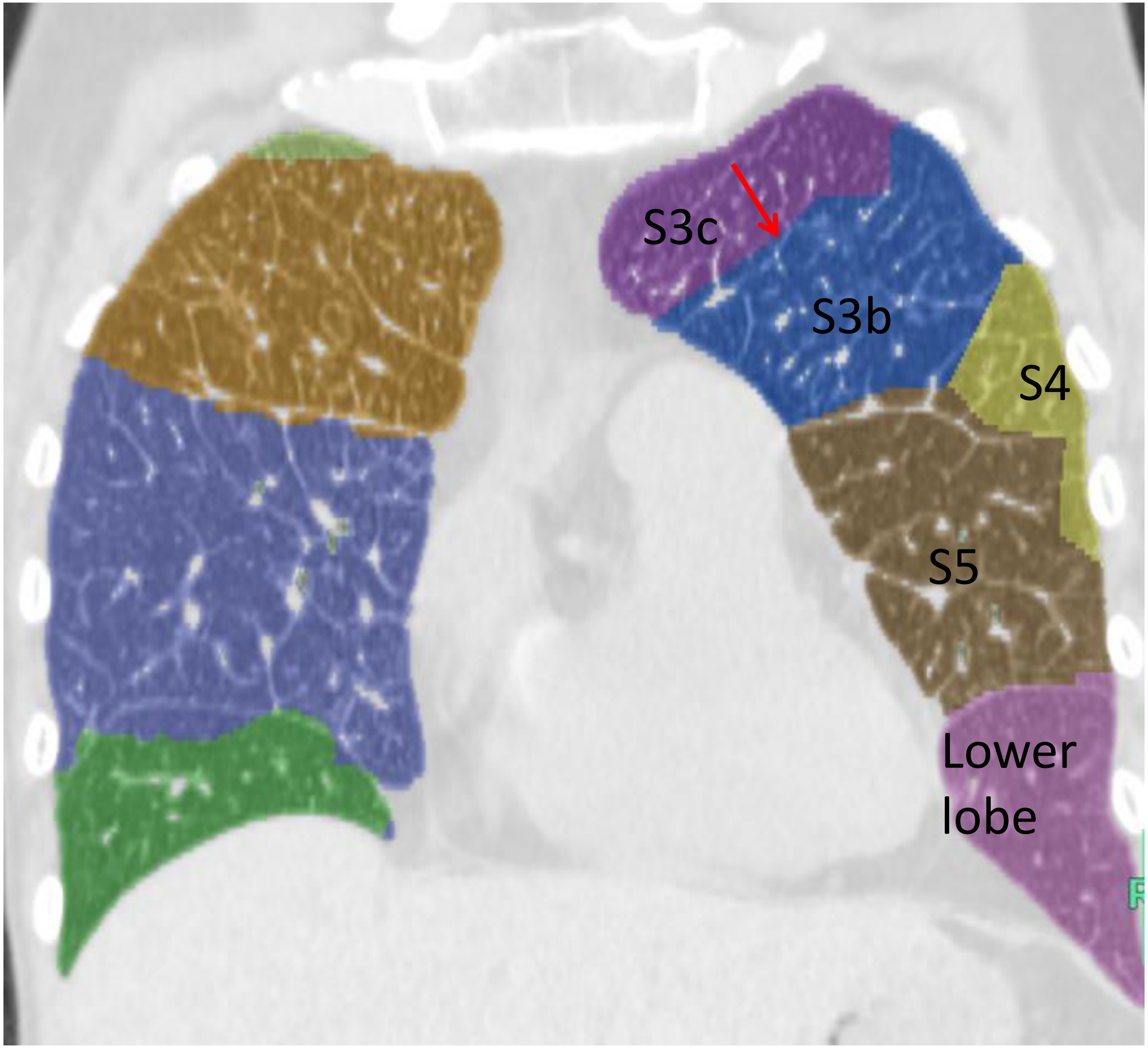

Fig S15a

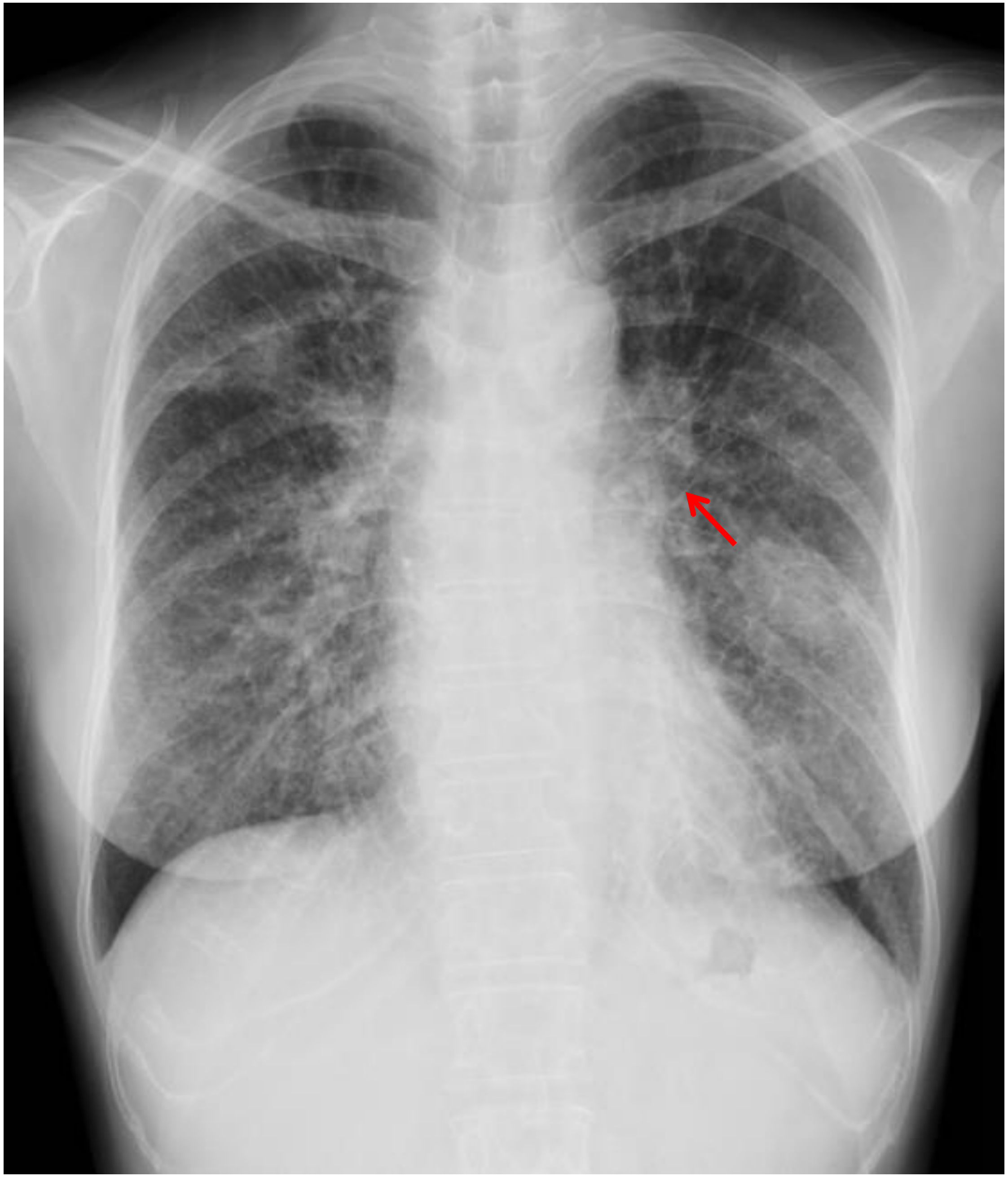

Fig S15b

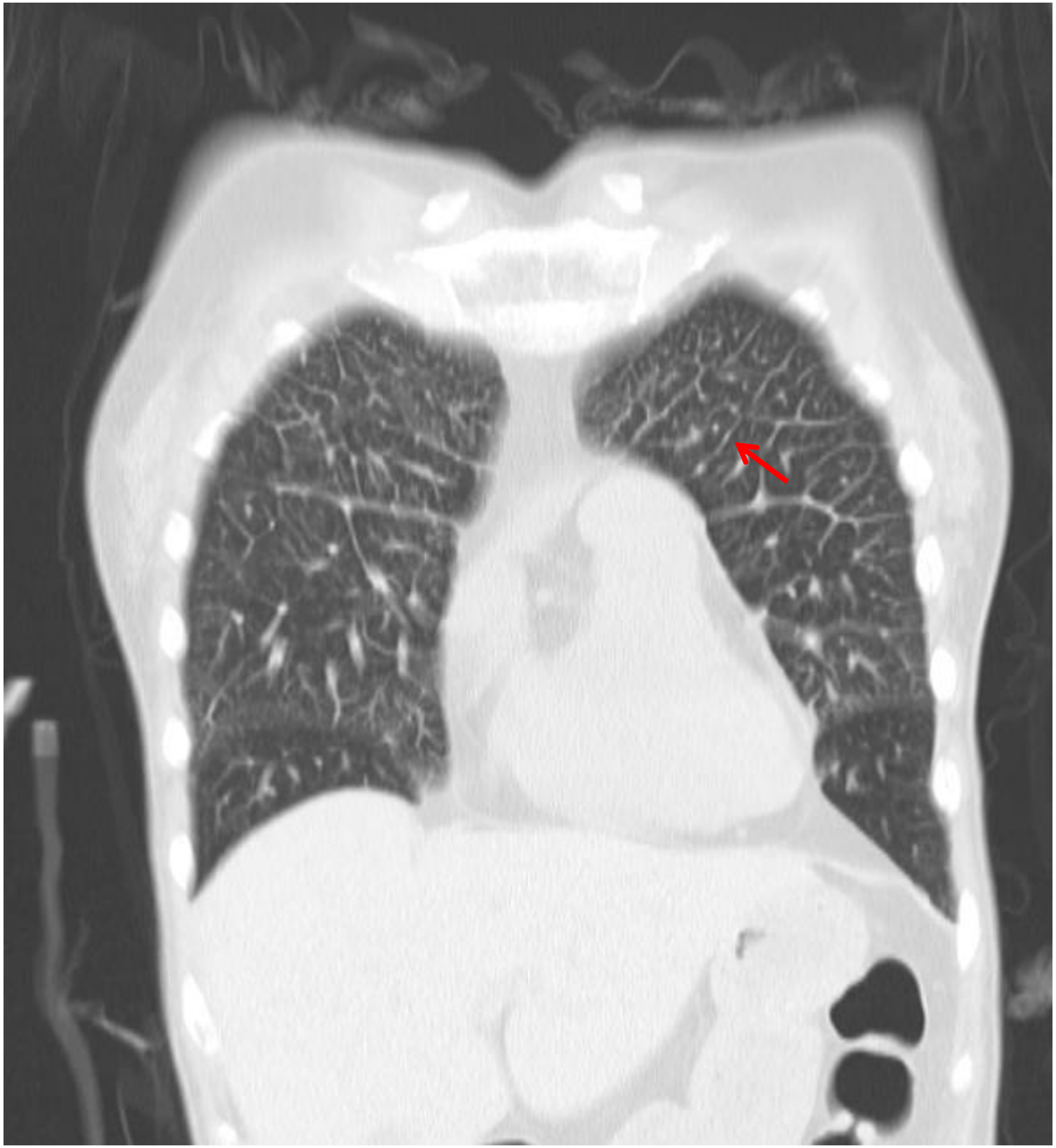

Fig S15c

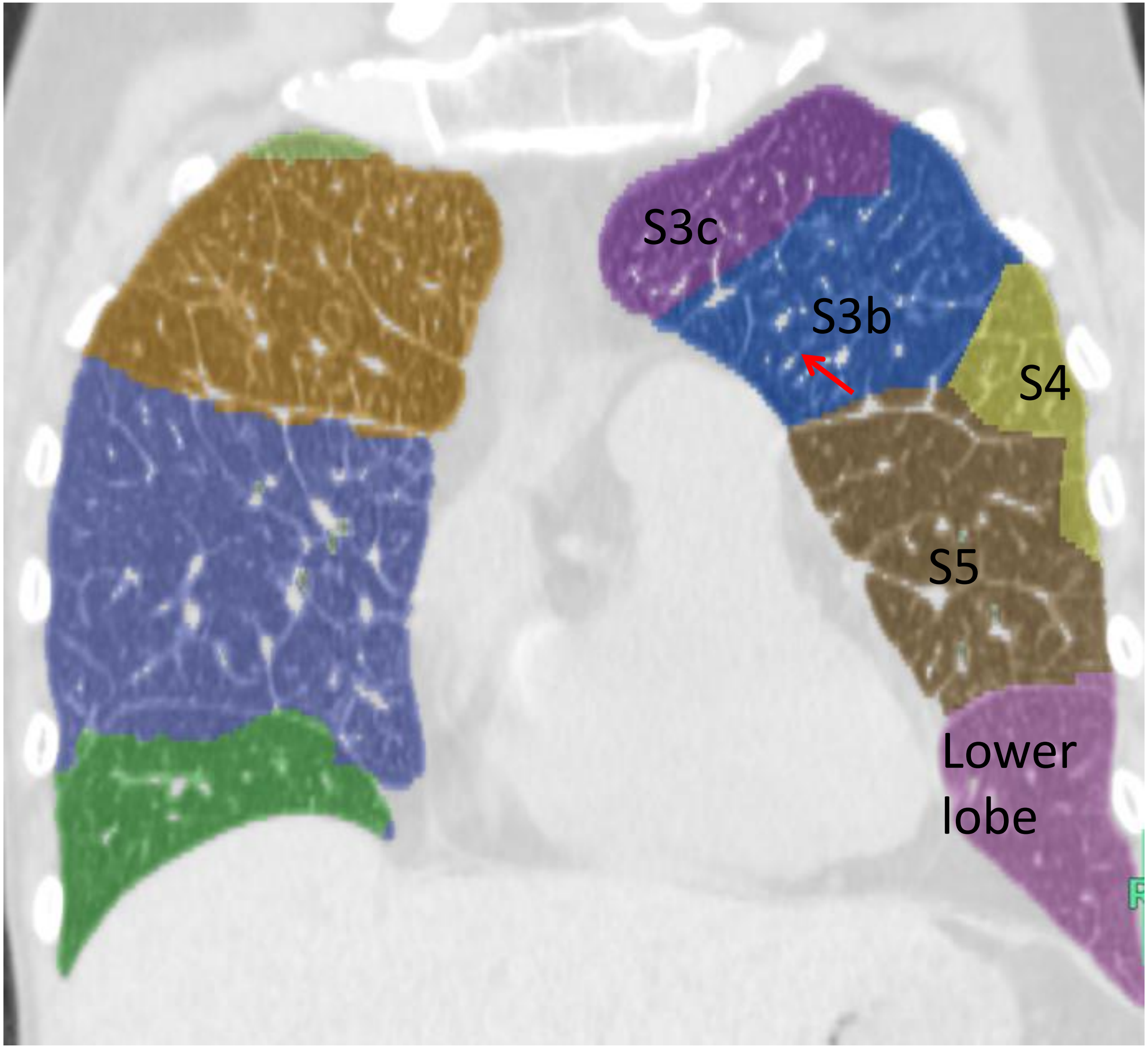

Fig S16a

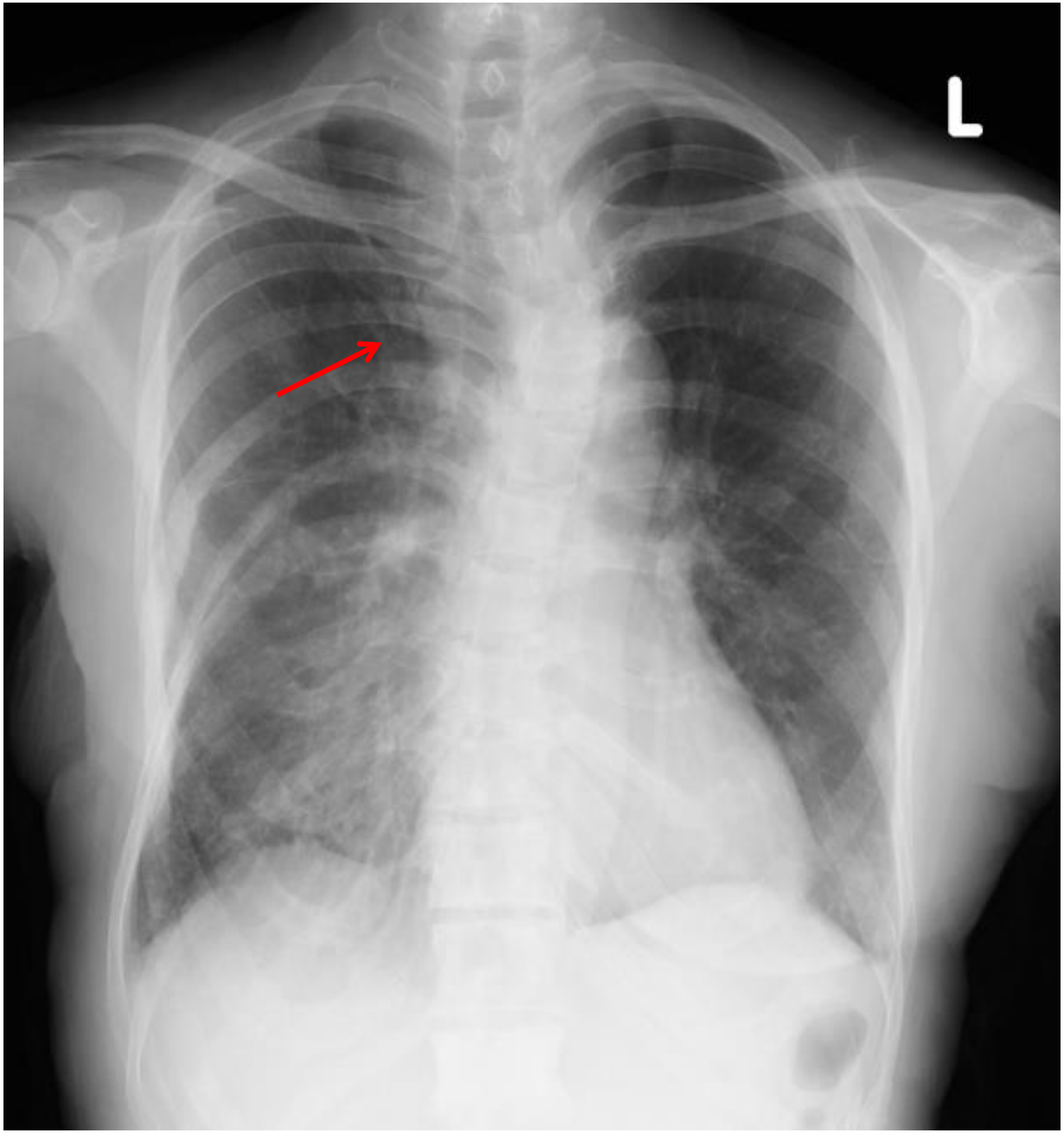

Fig S16b

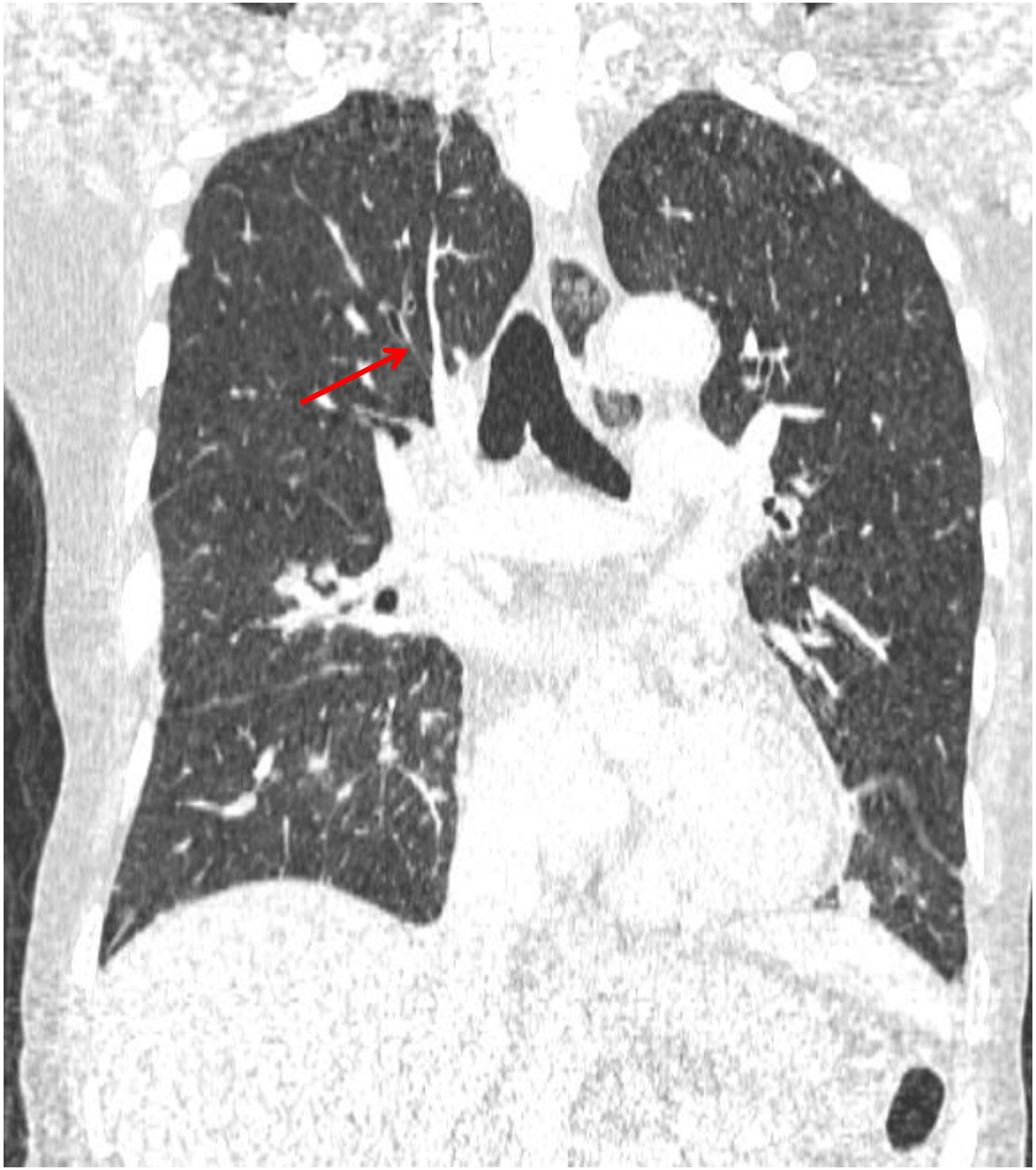

Fig S16c

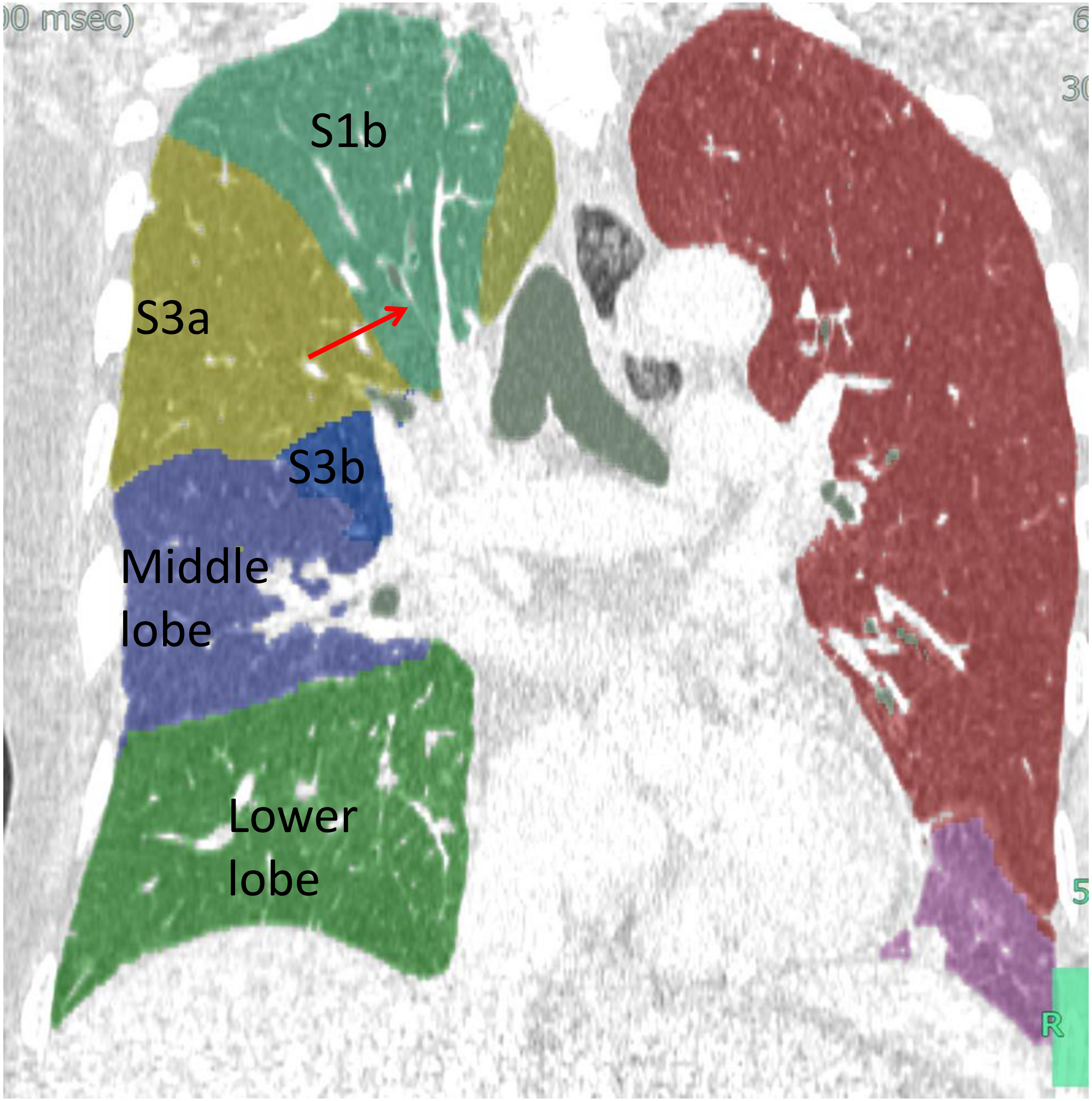

Fig S17a

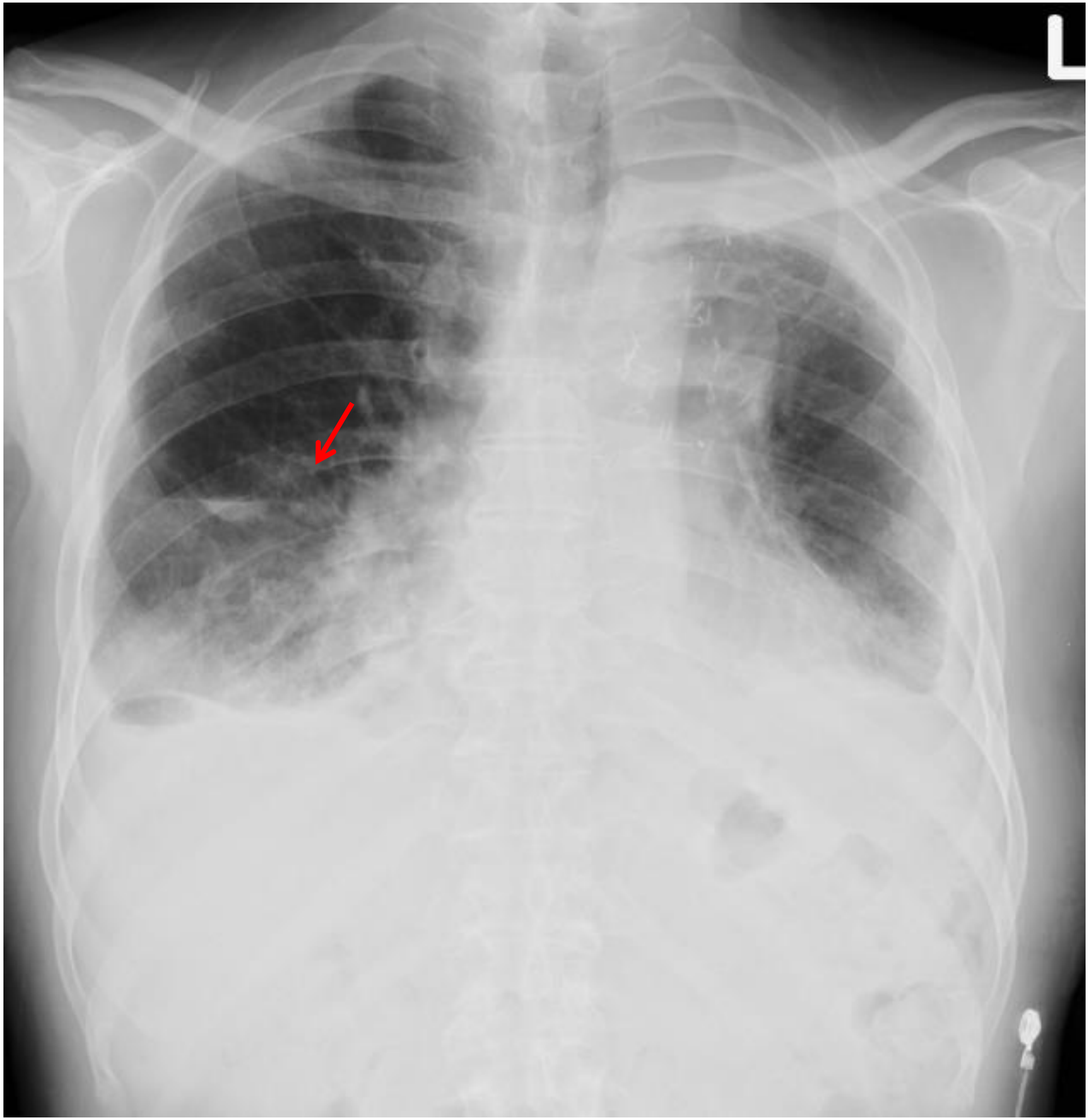

Fig S17b

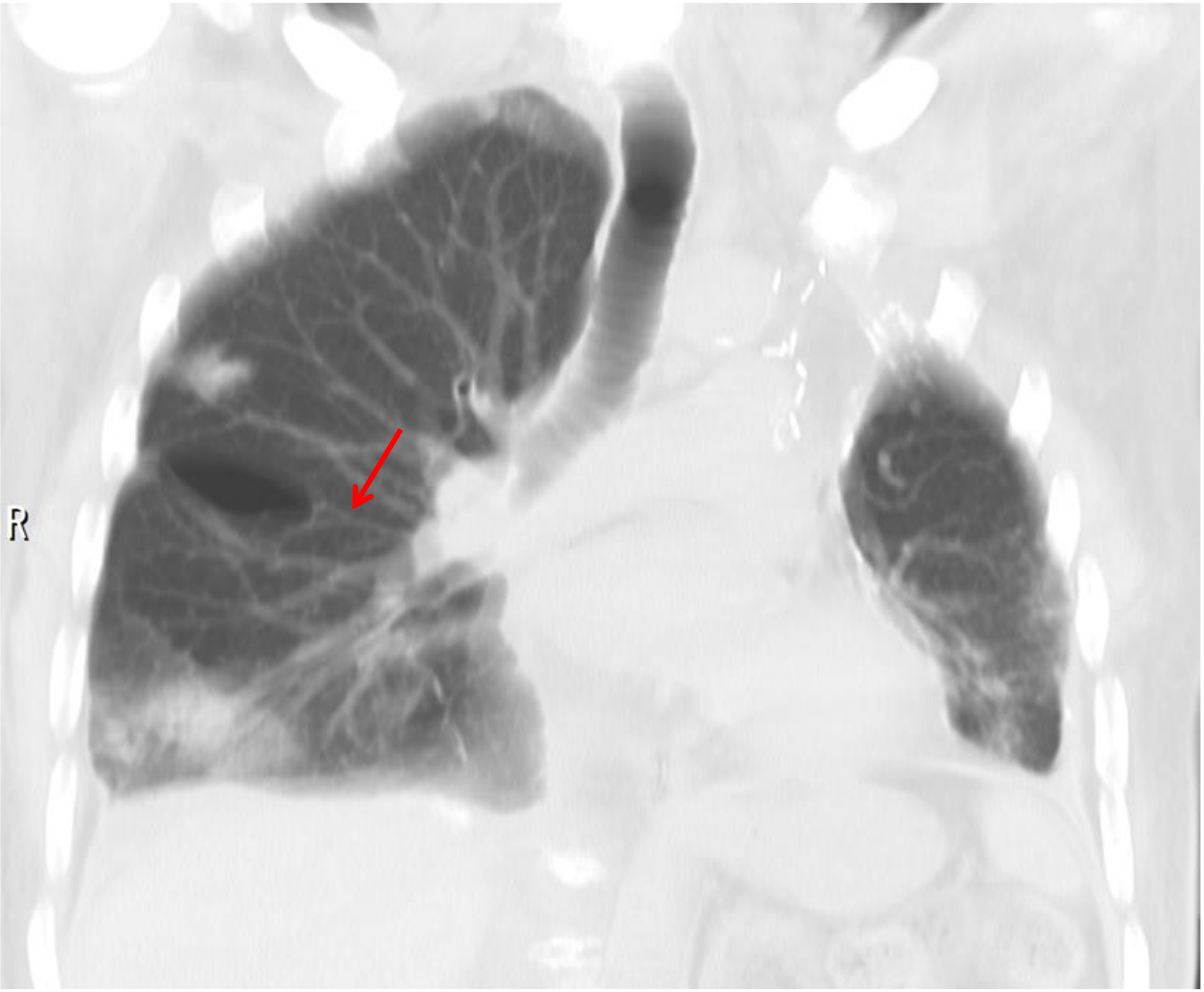

Fig S17c

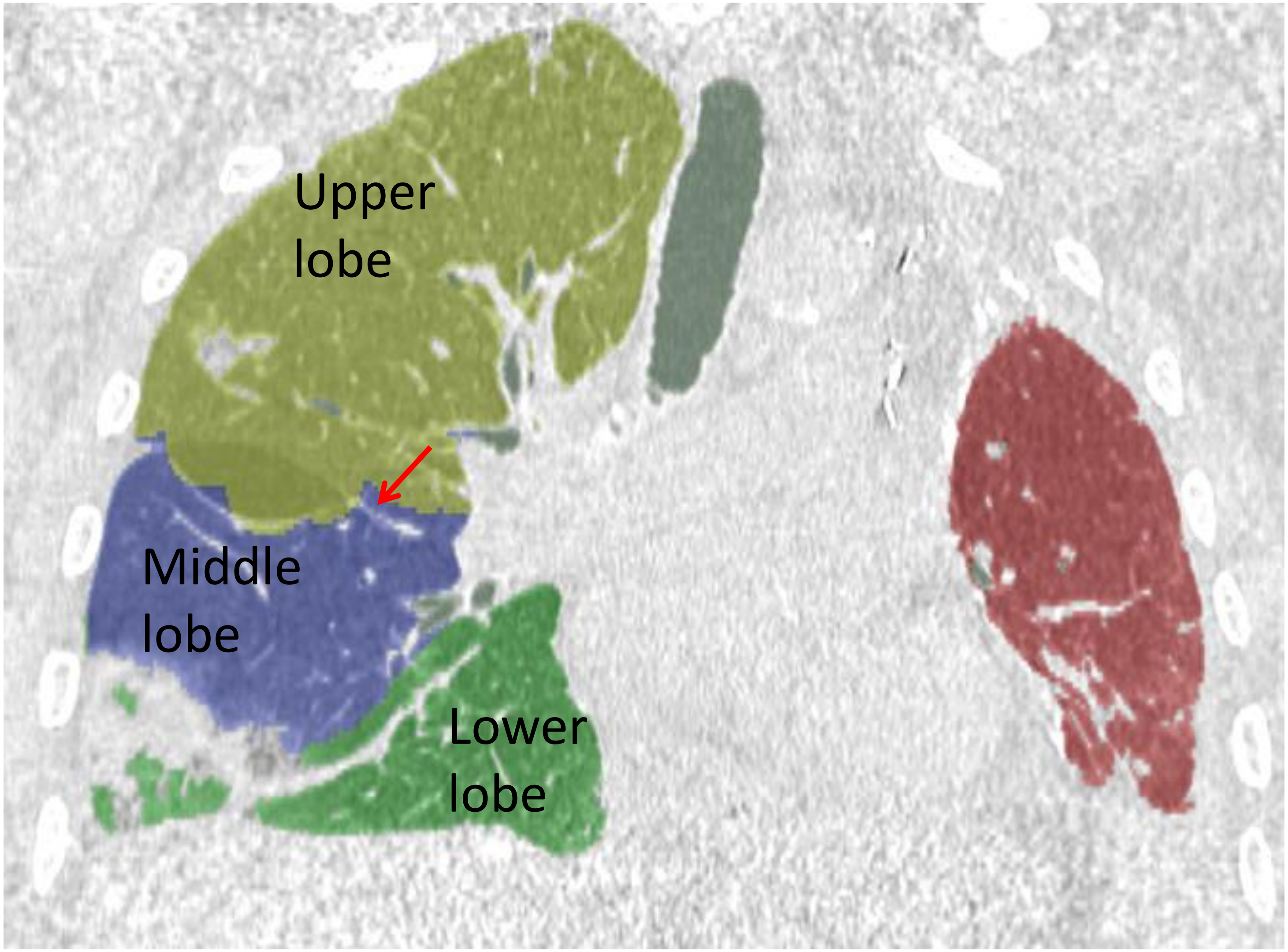

Fig S18a

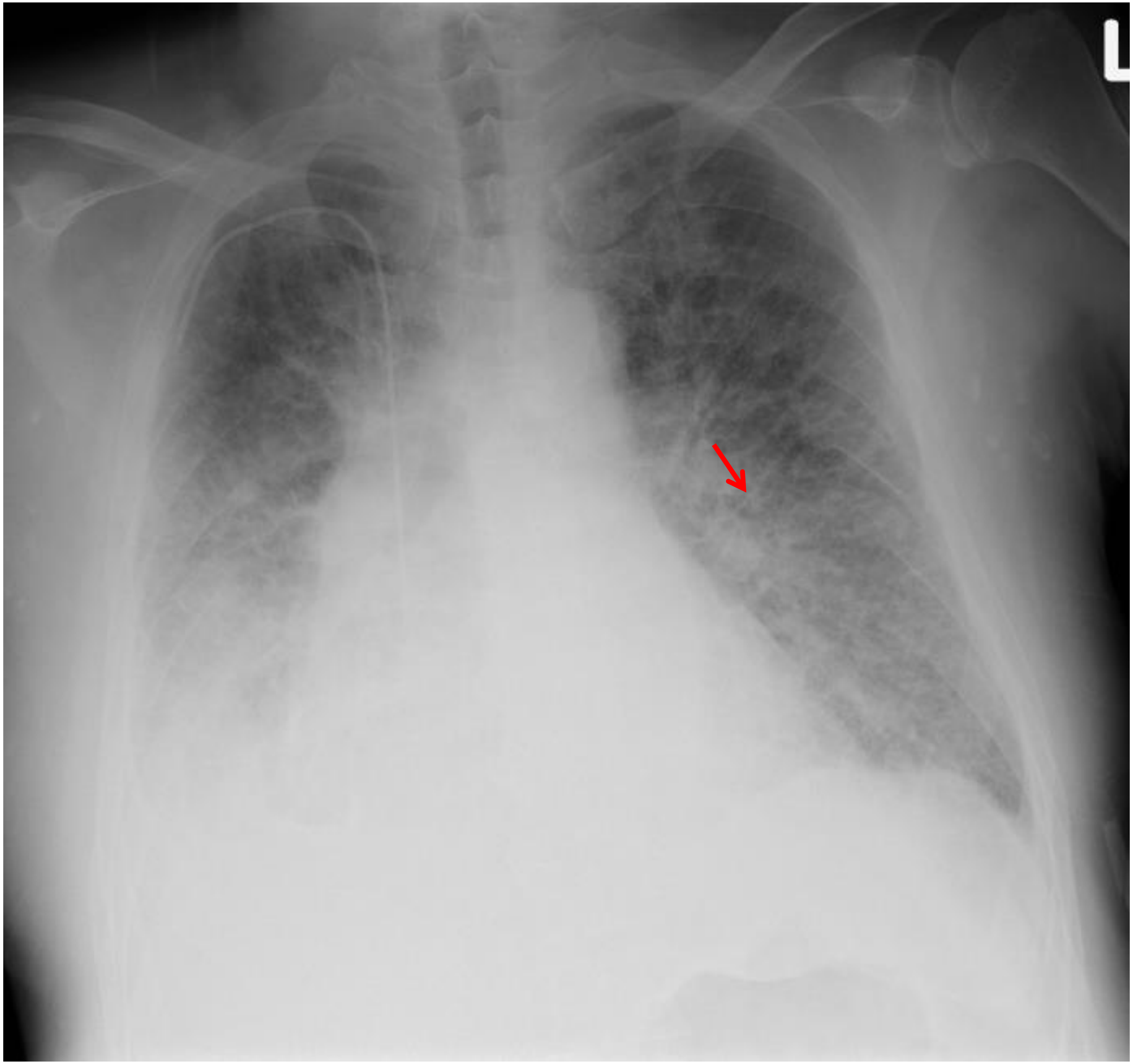

Fig S18b

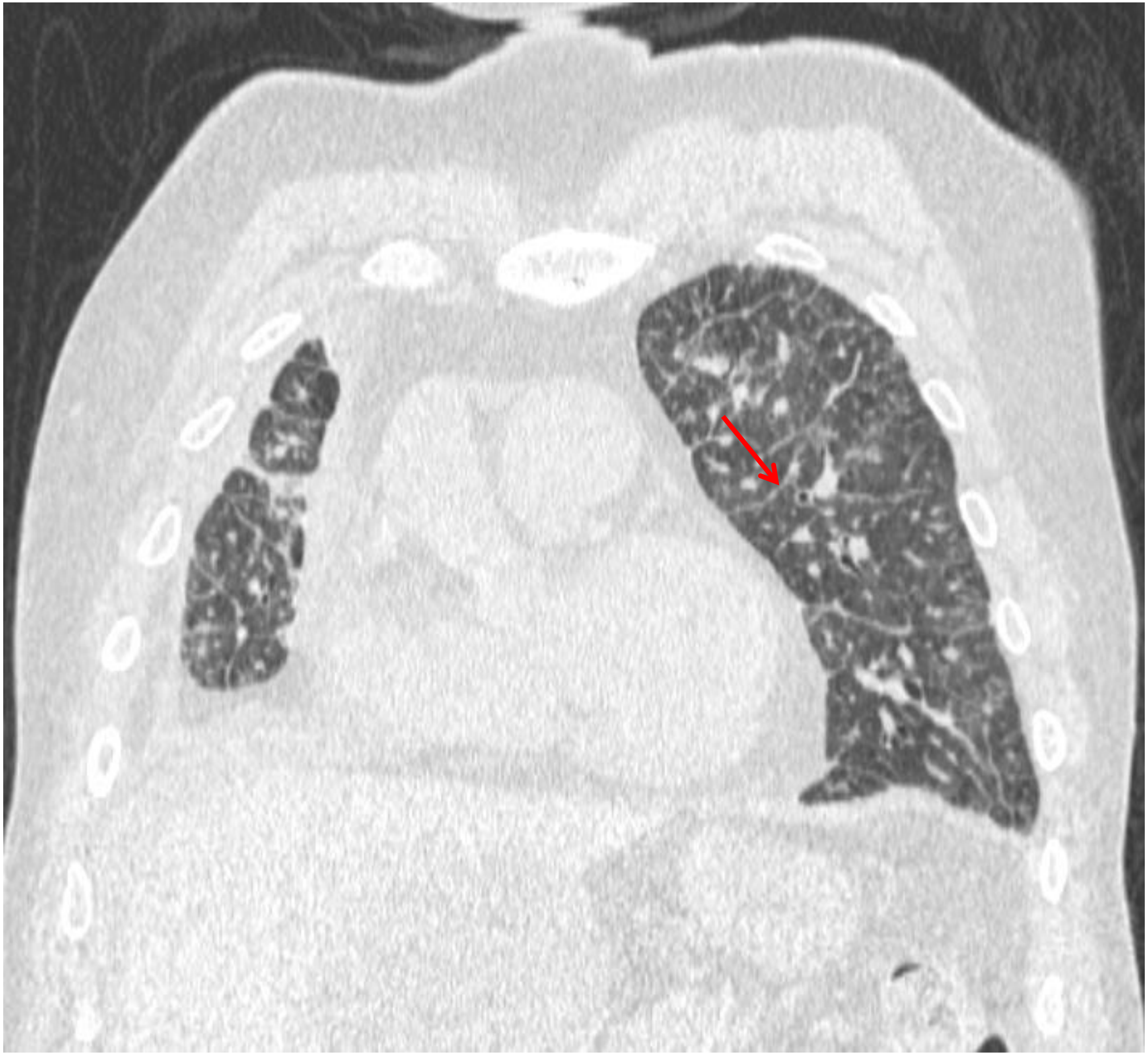

Fig S18c

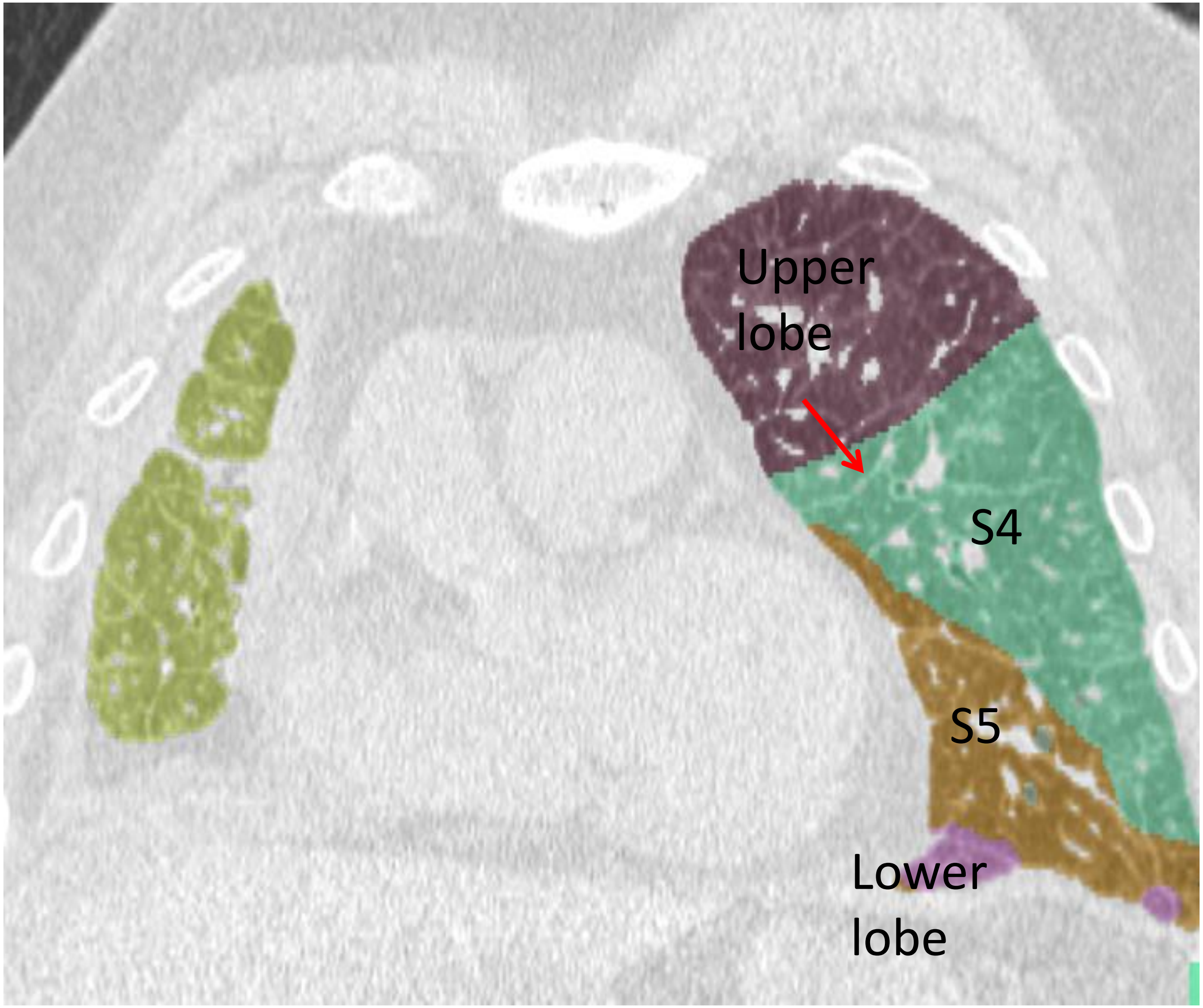

Fig S19a

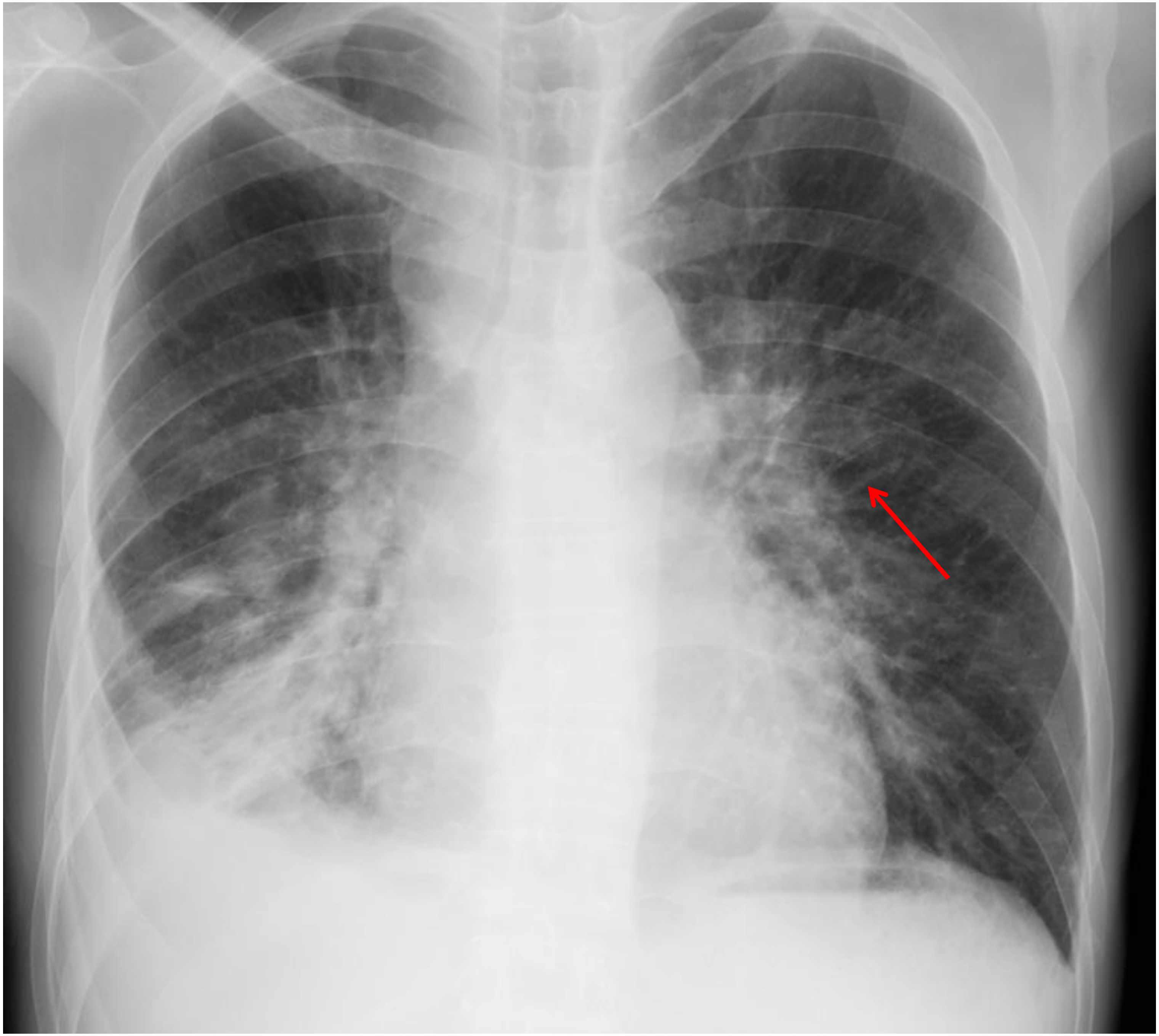

Fig S19b

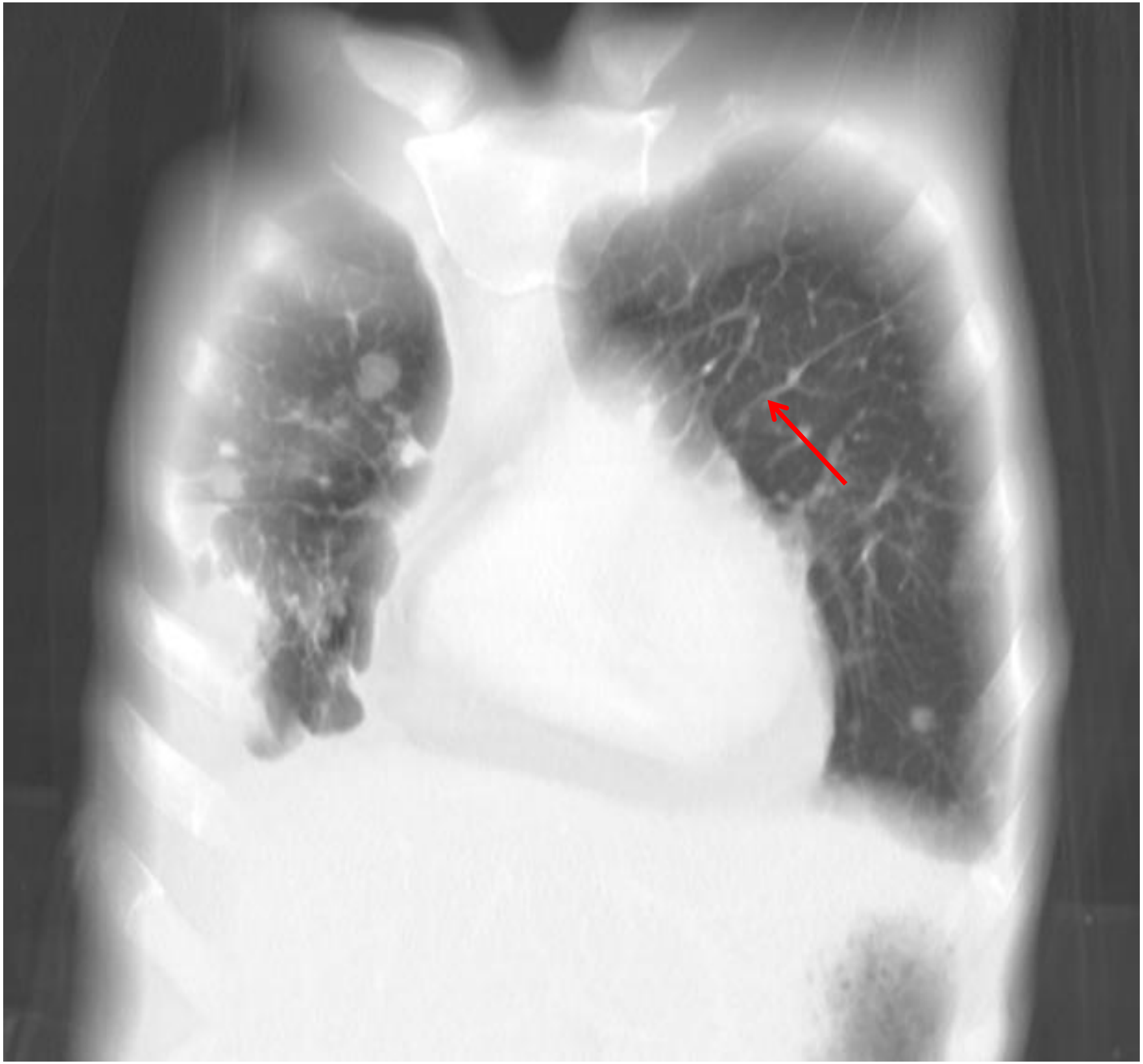

Fig S19c

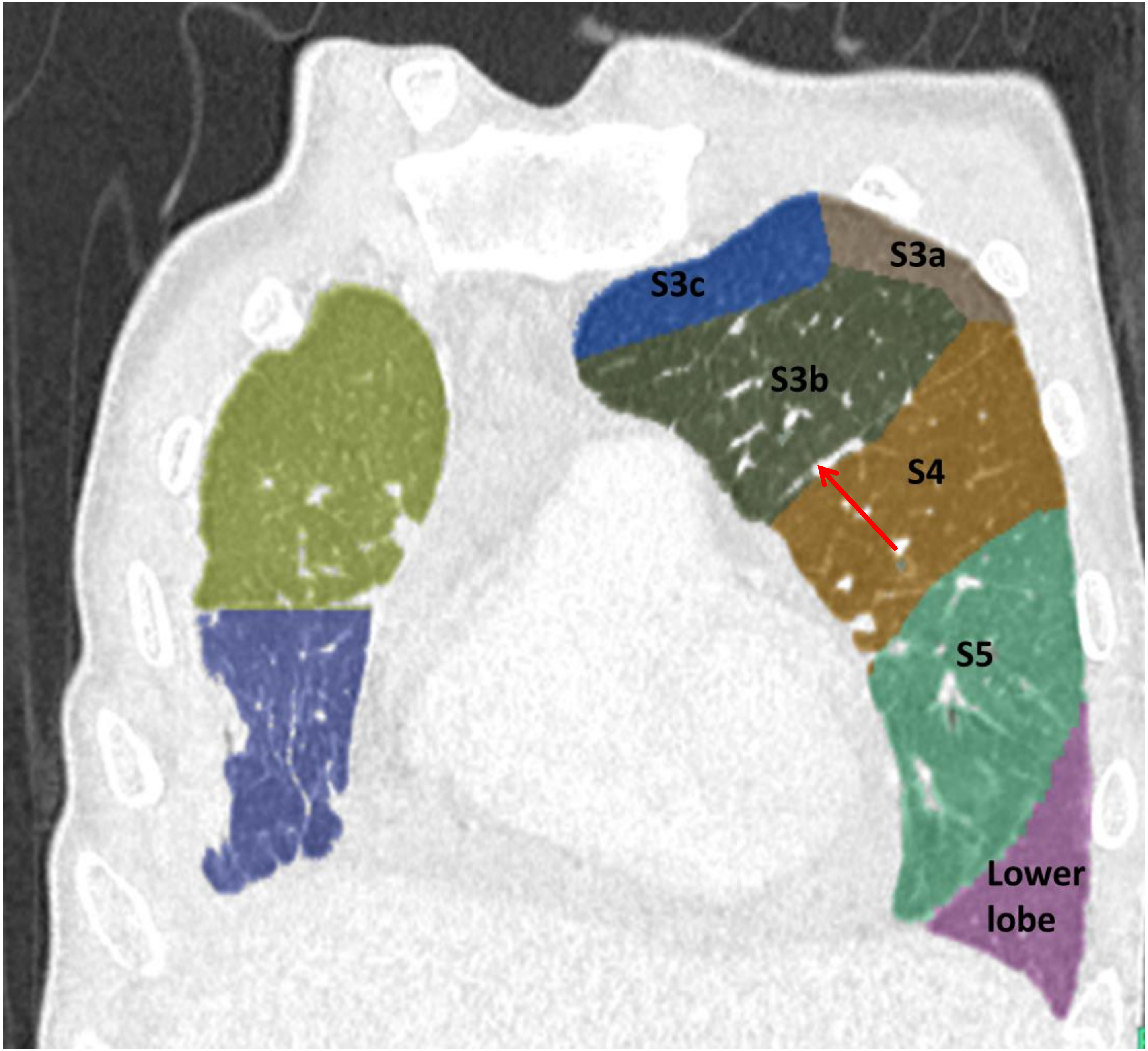

Fig S20a

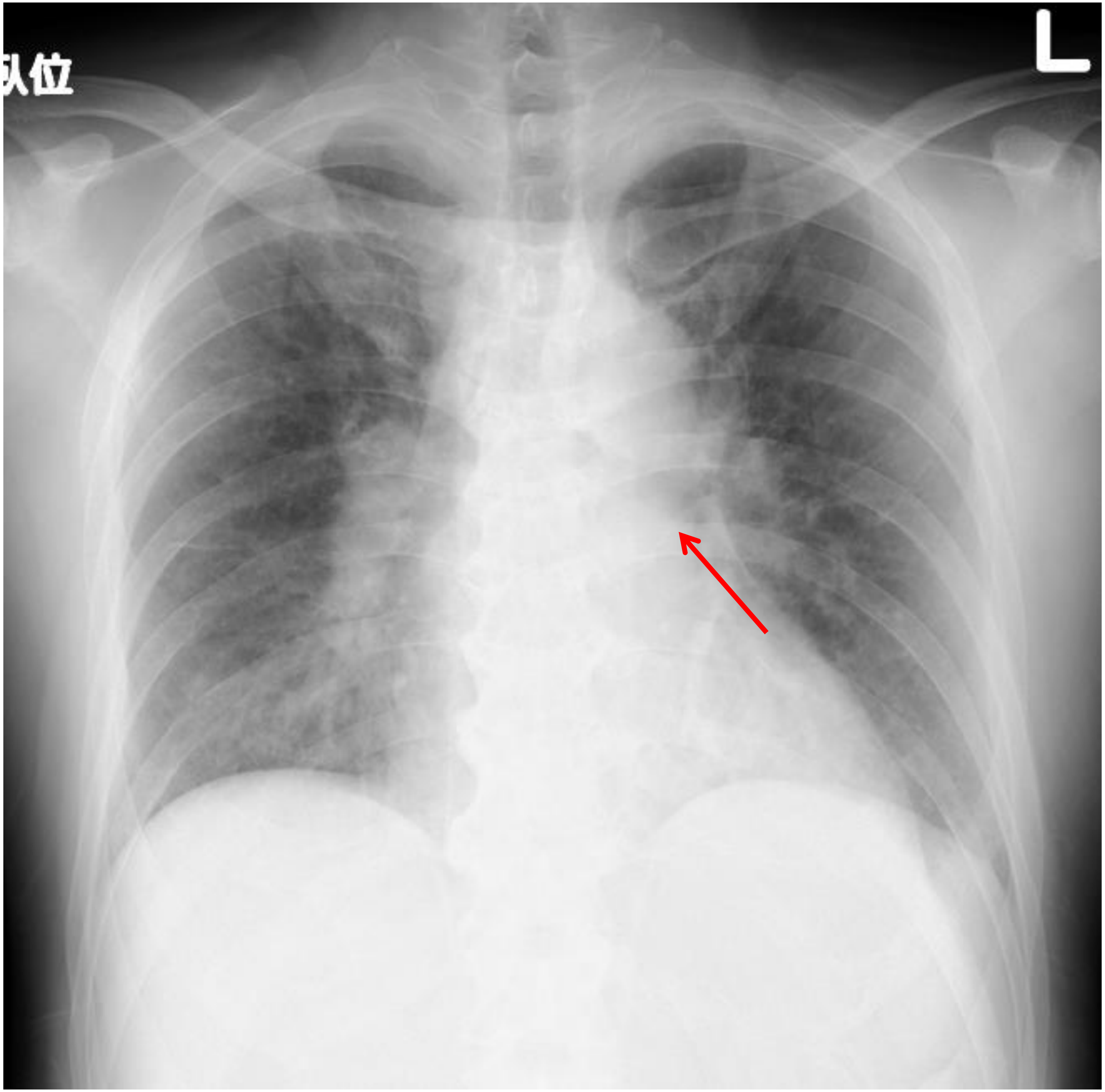

Fig S20b

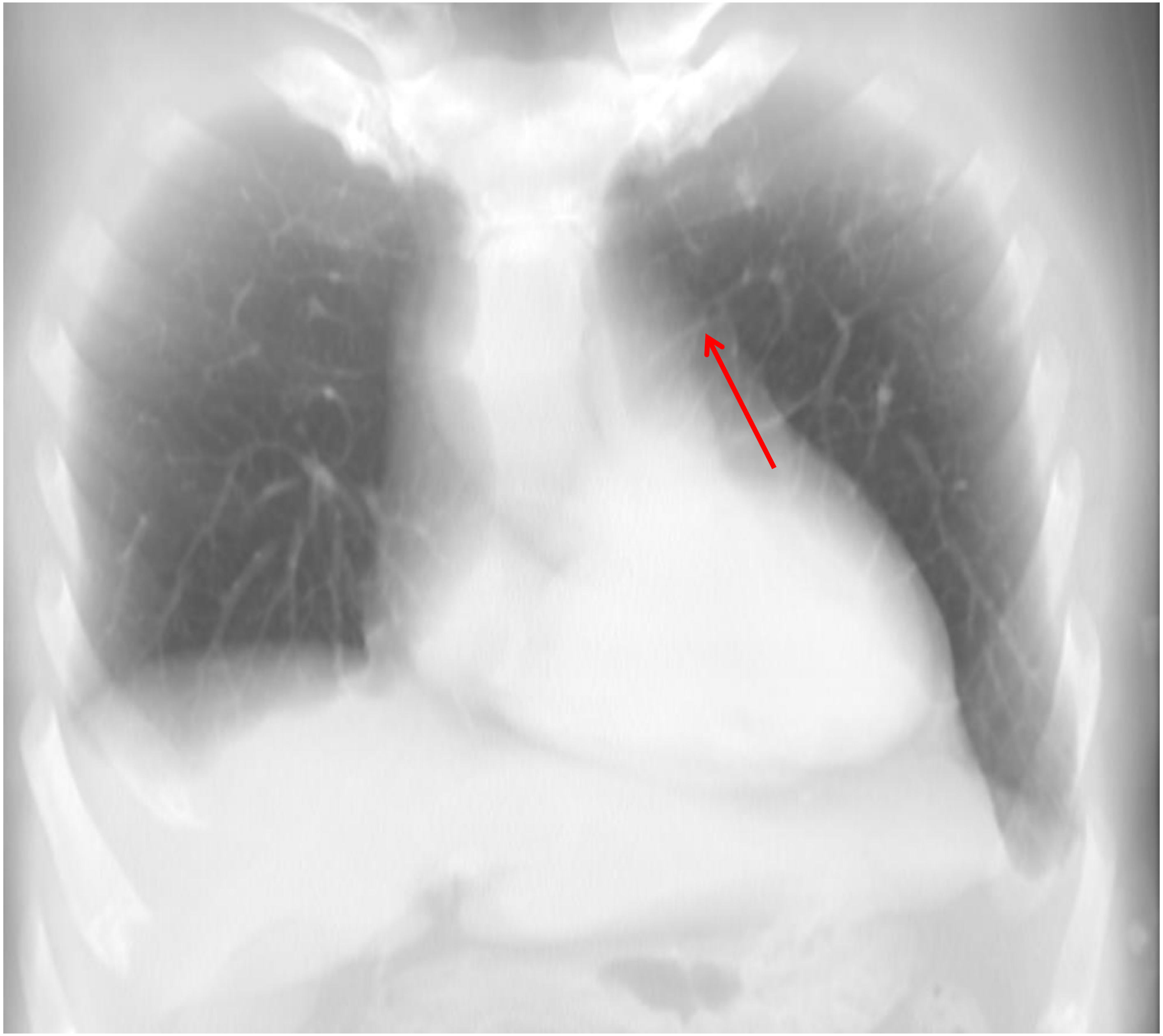

Fig S20c

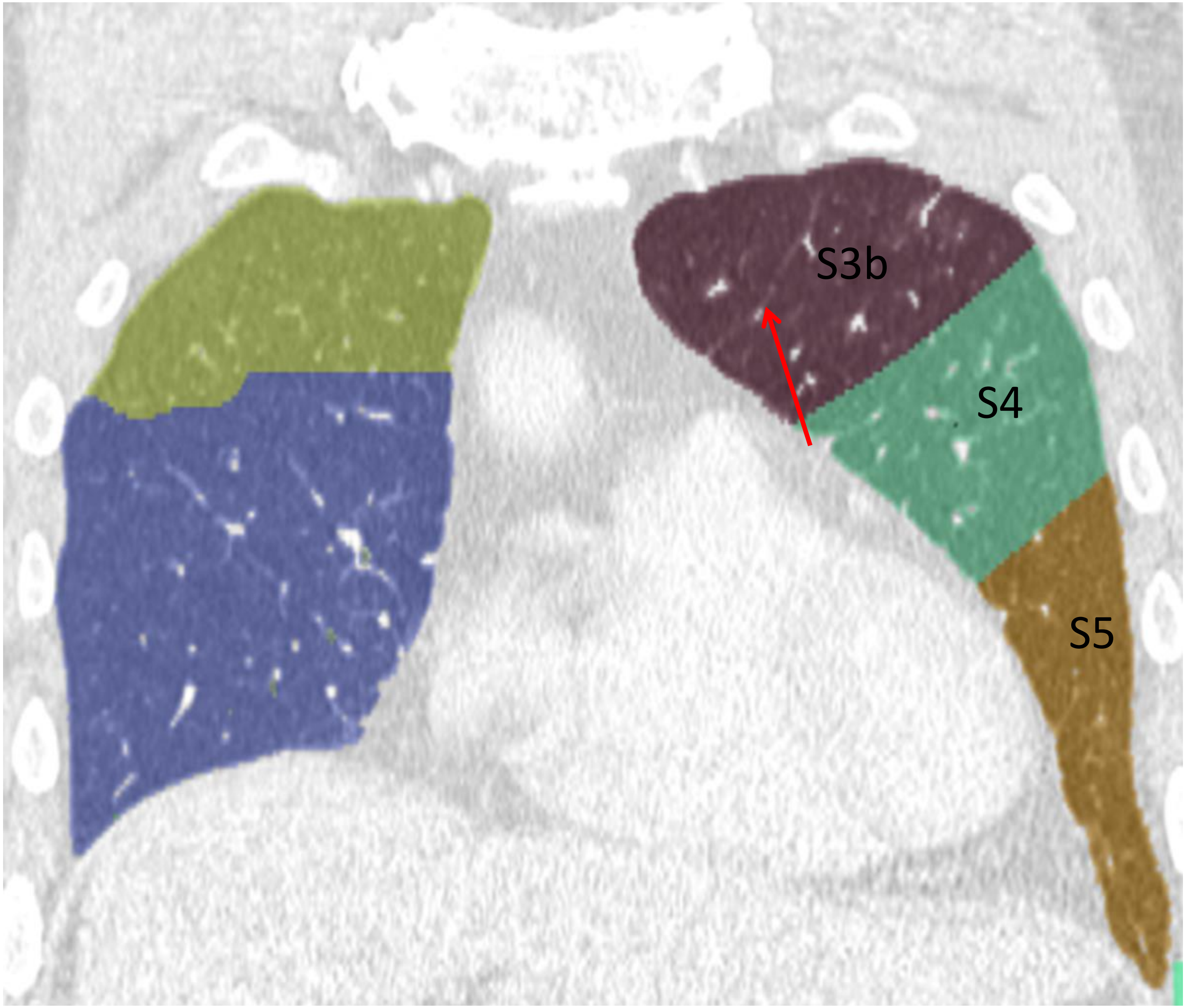

Fig S21a

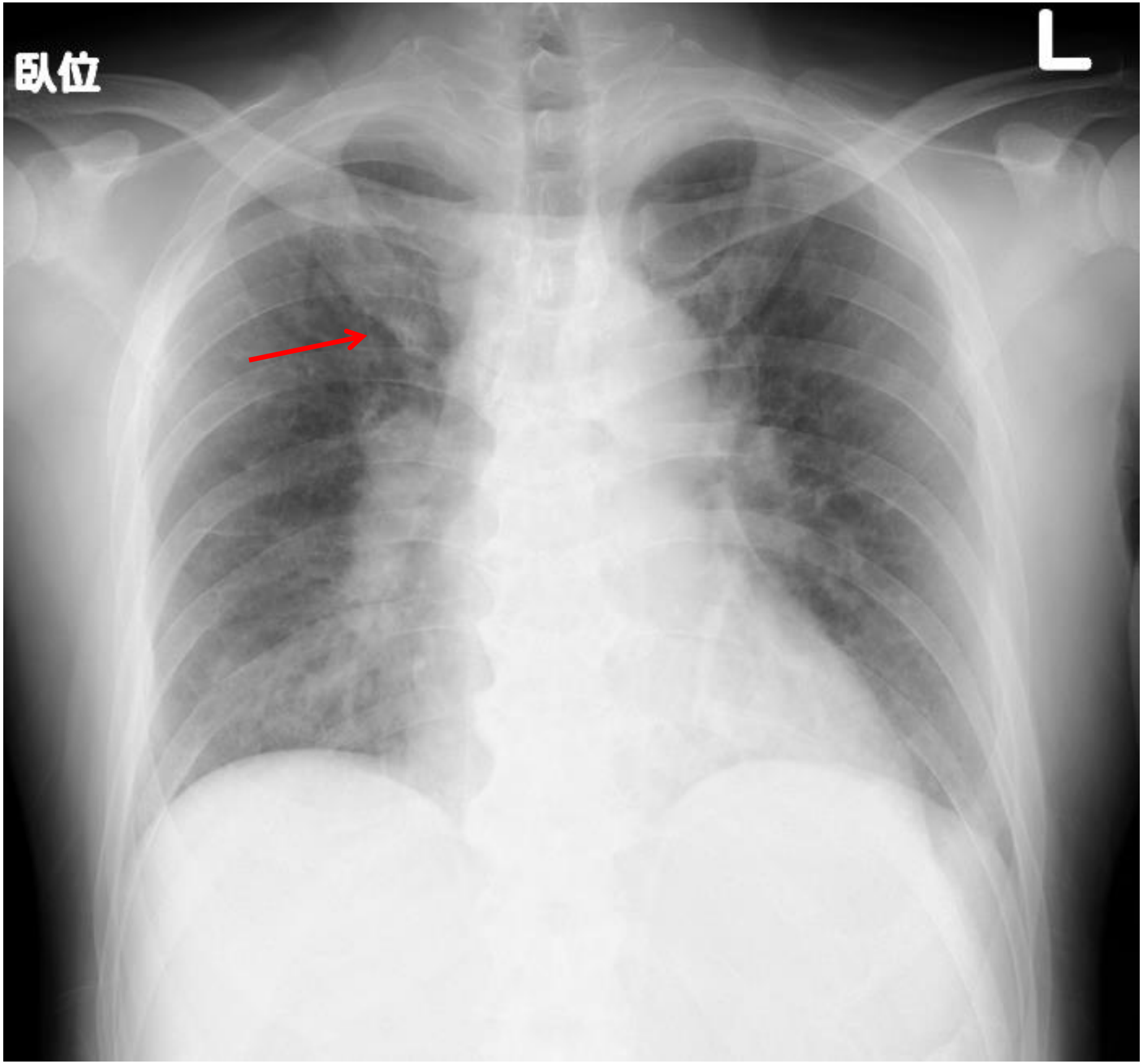

Fig S21b

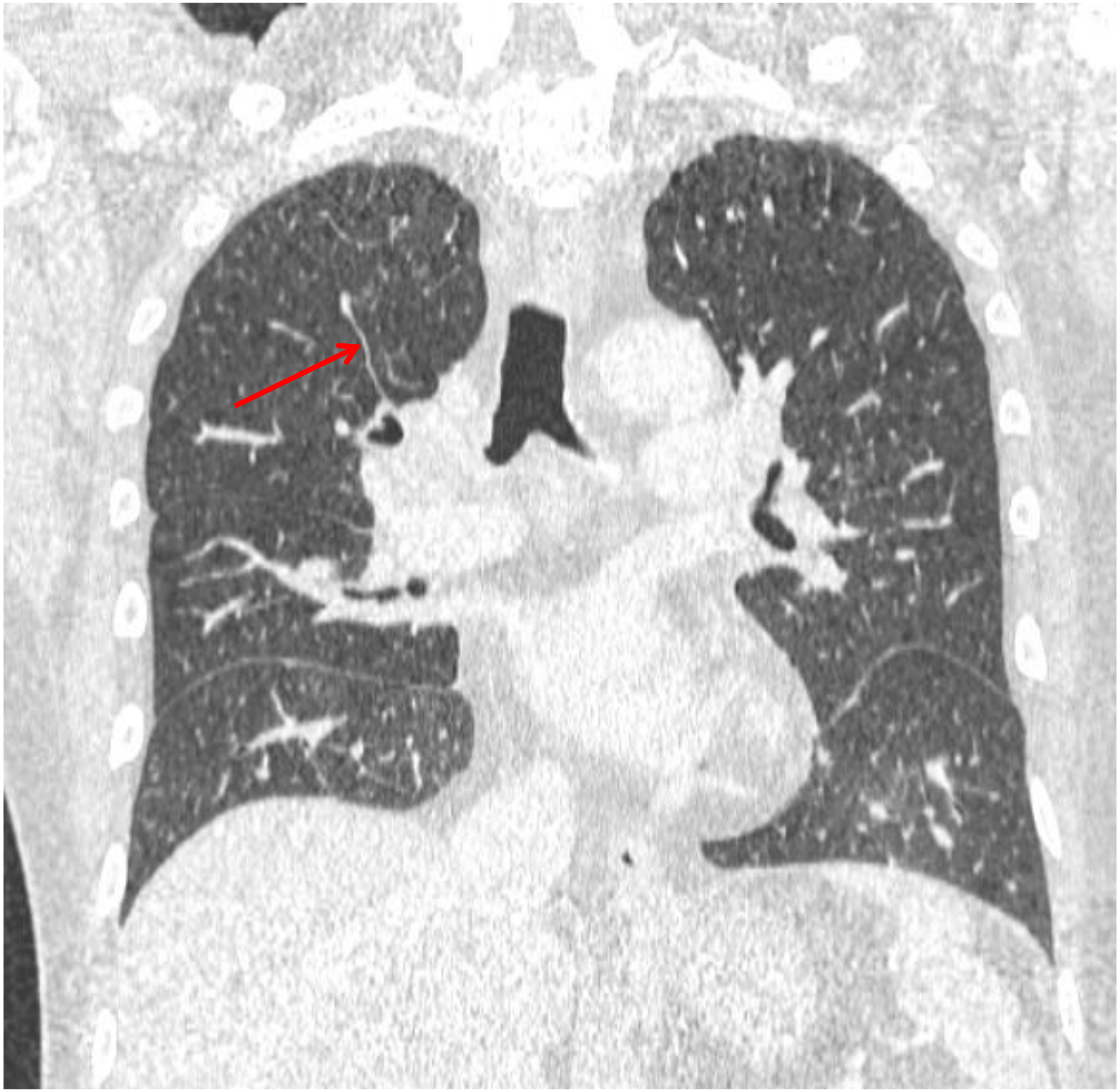

Fig S21c

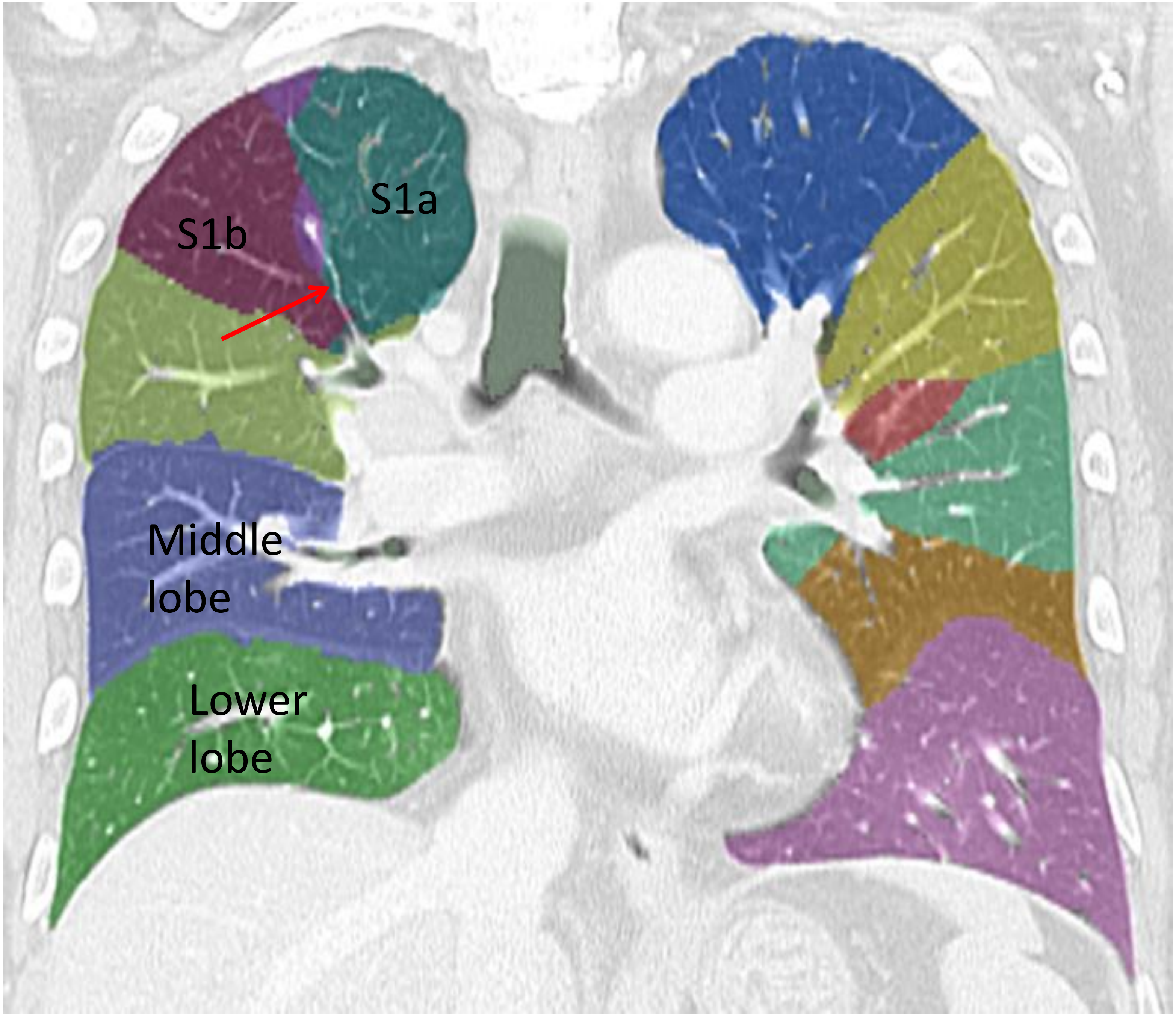

Fig S22a

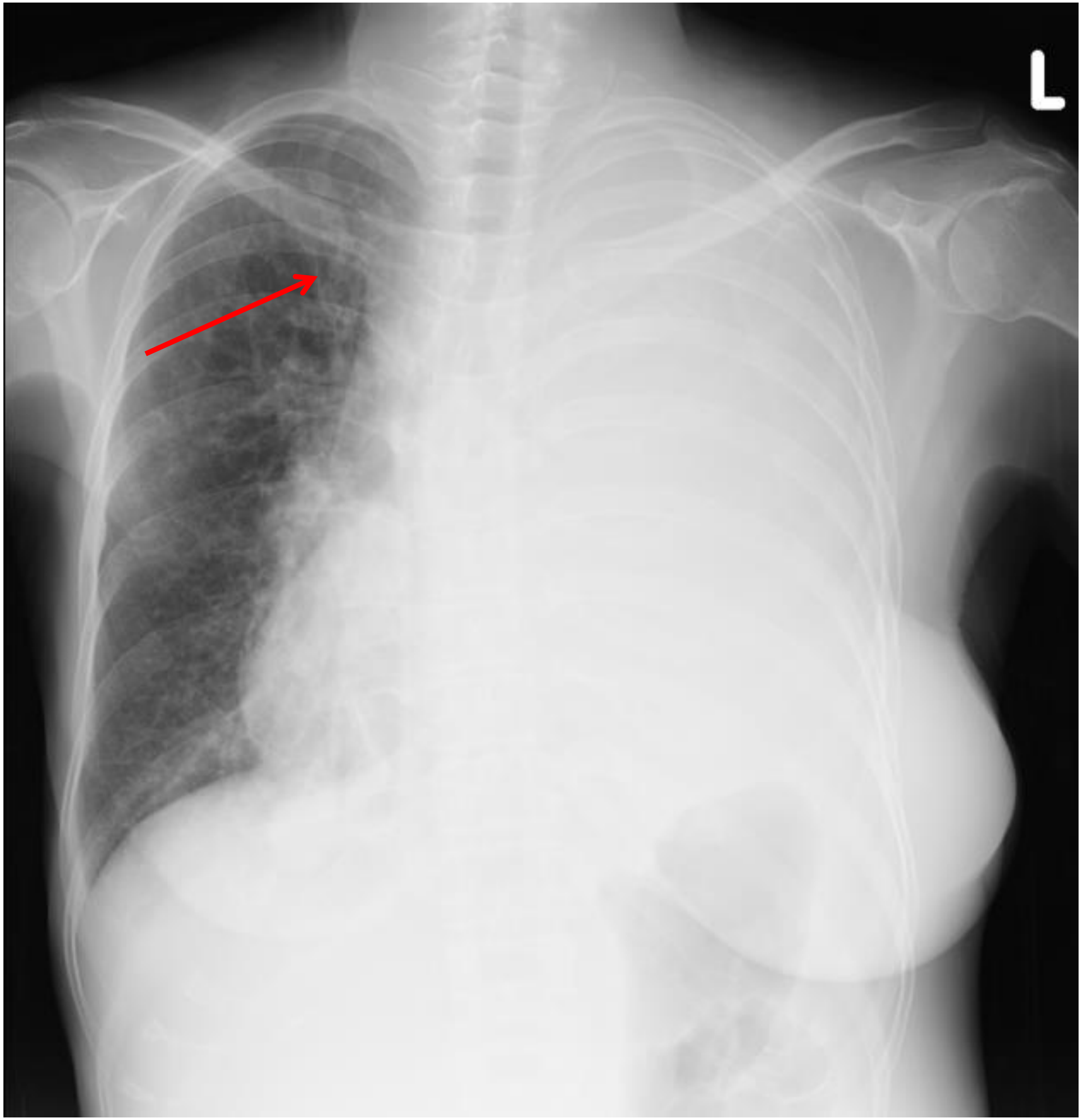

Fig S22b

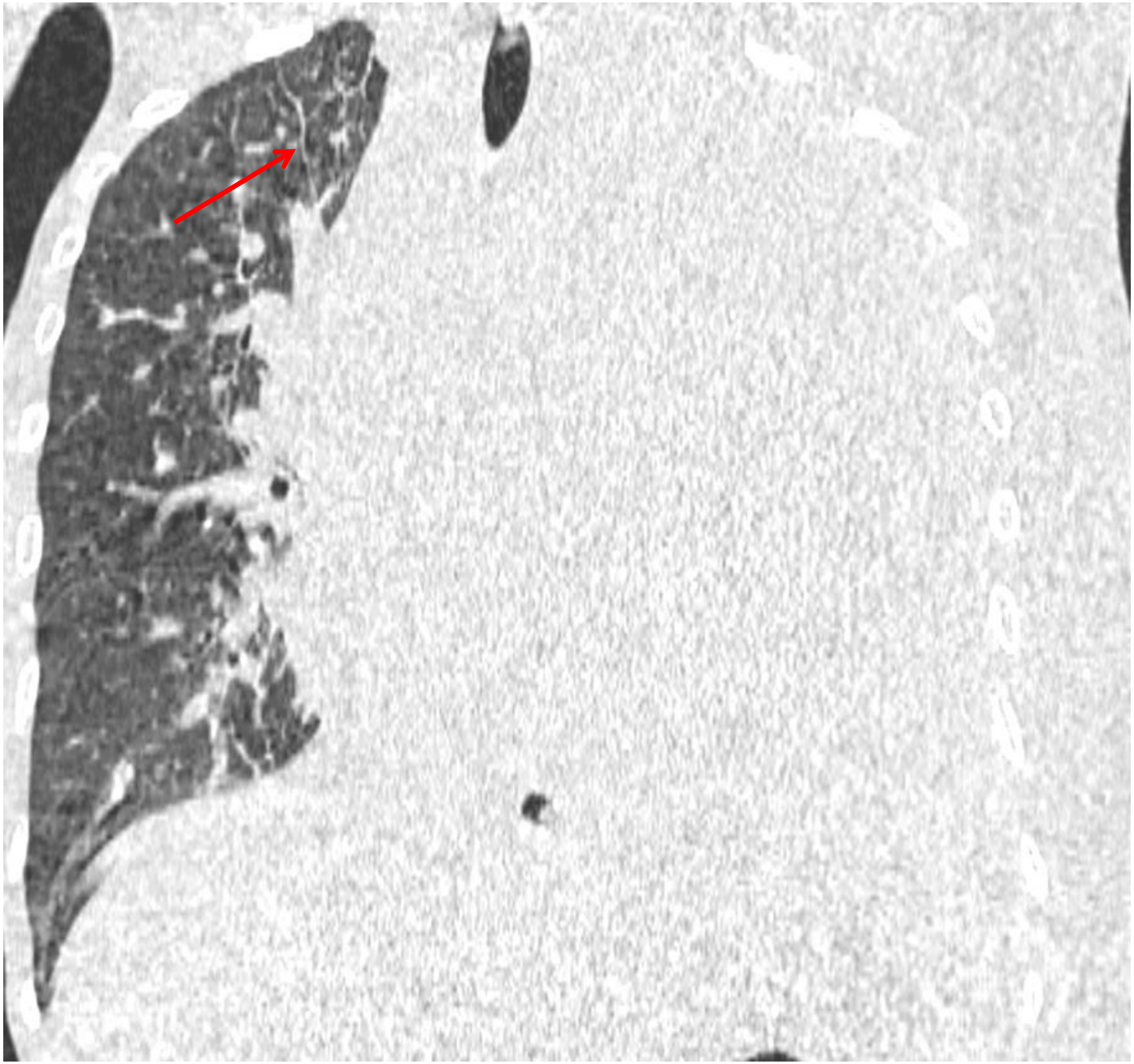

Fig S22c

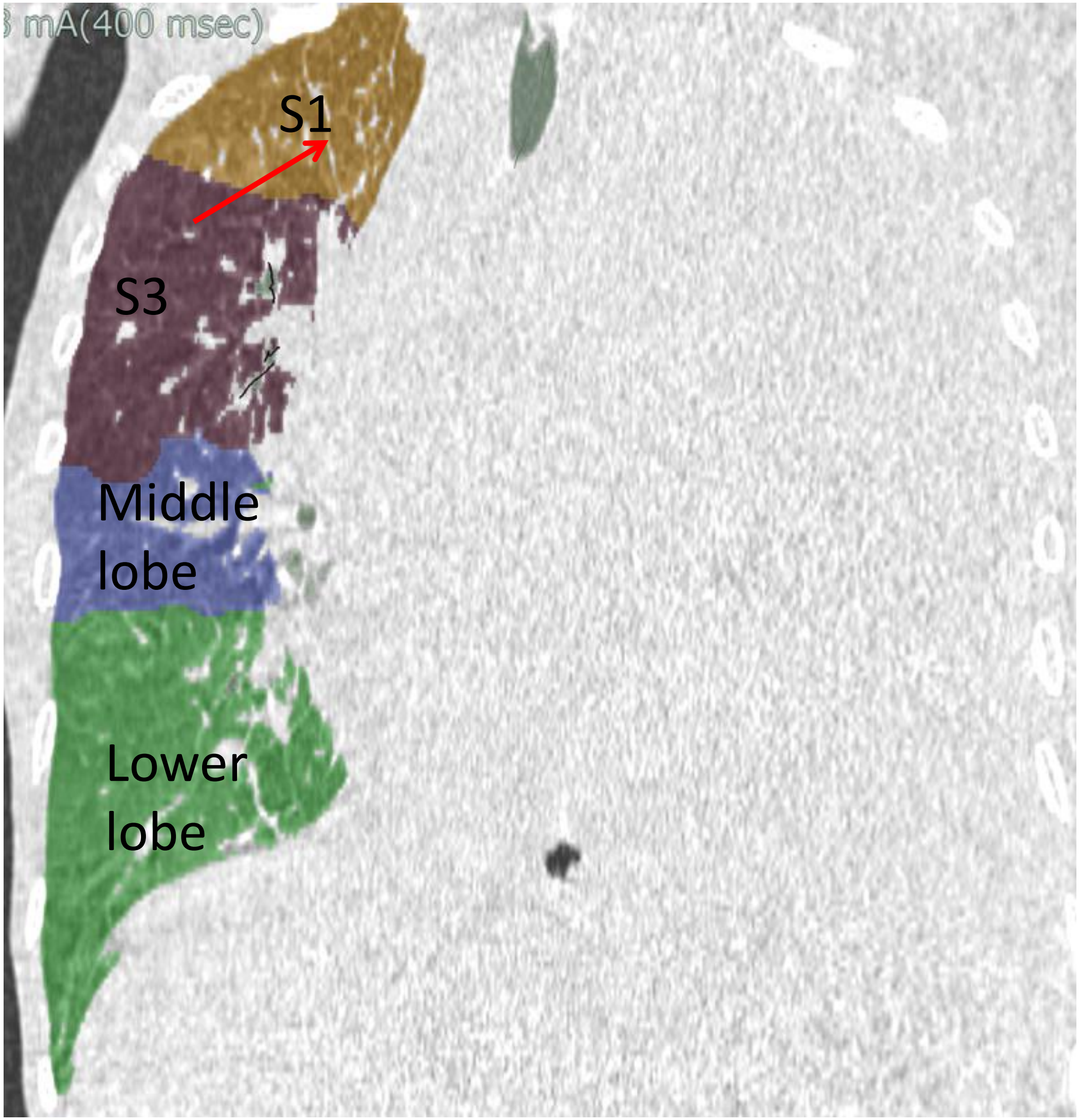

Fig S23a

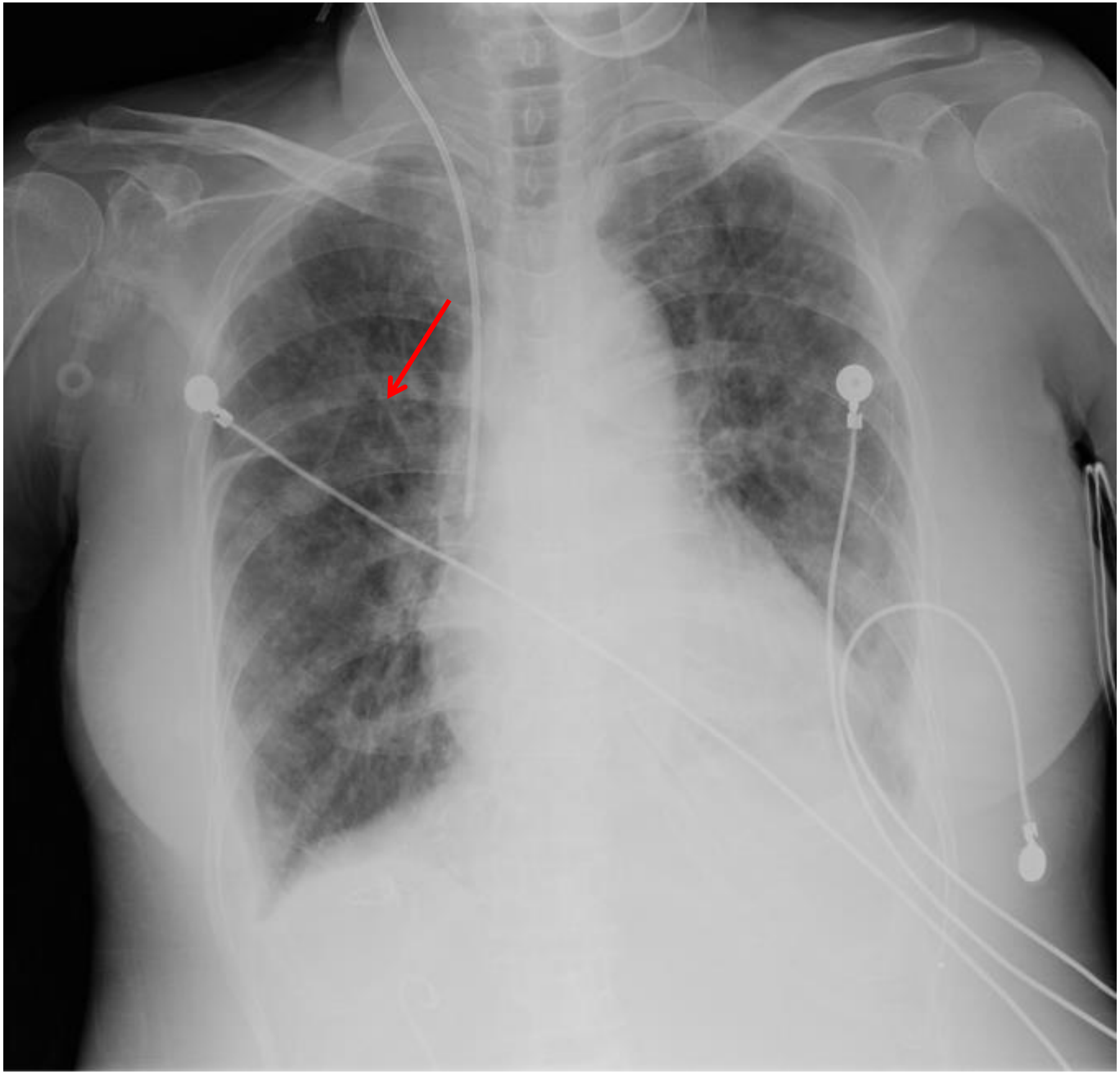

Fig S23b

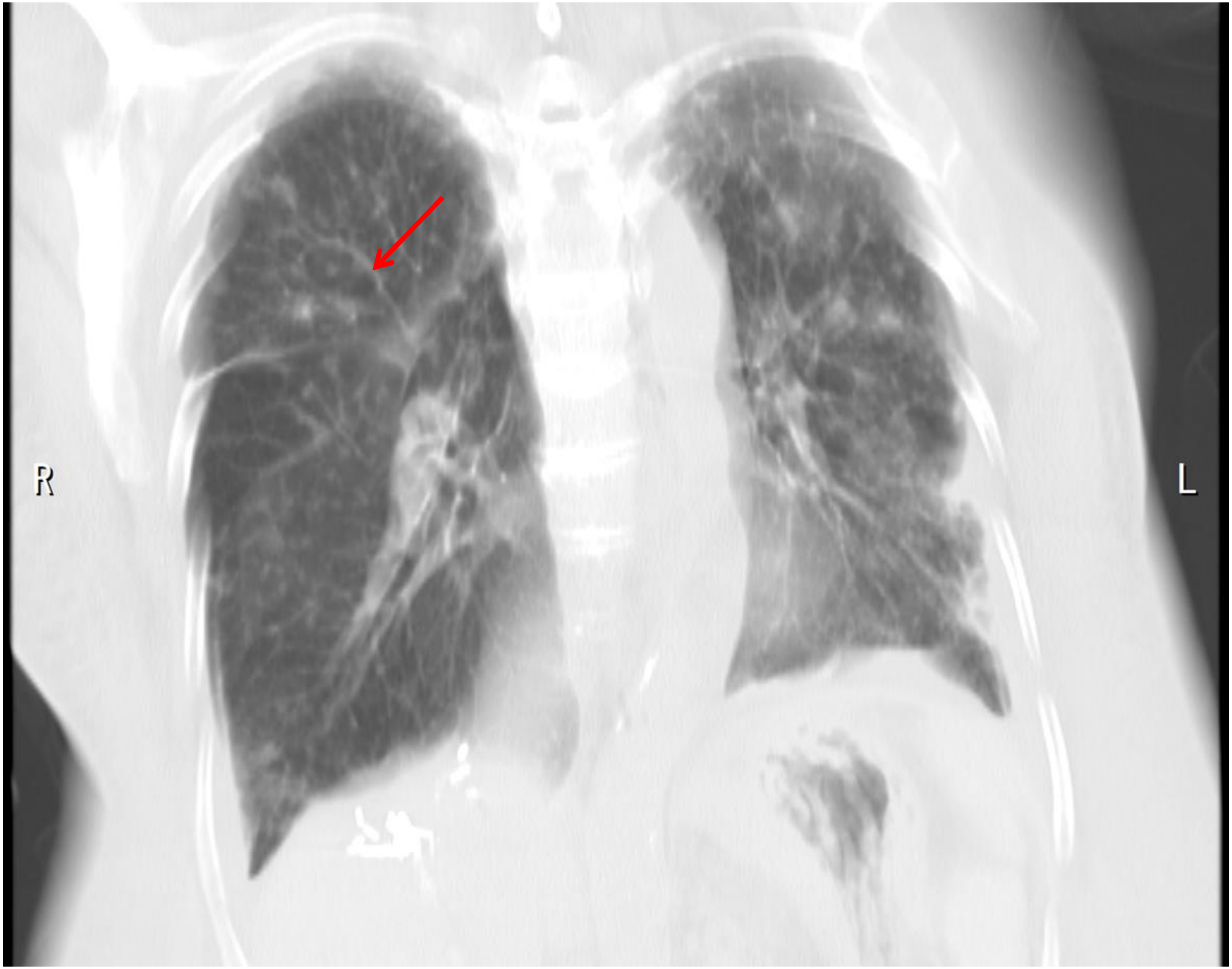

Fig S23c

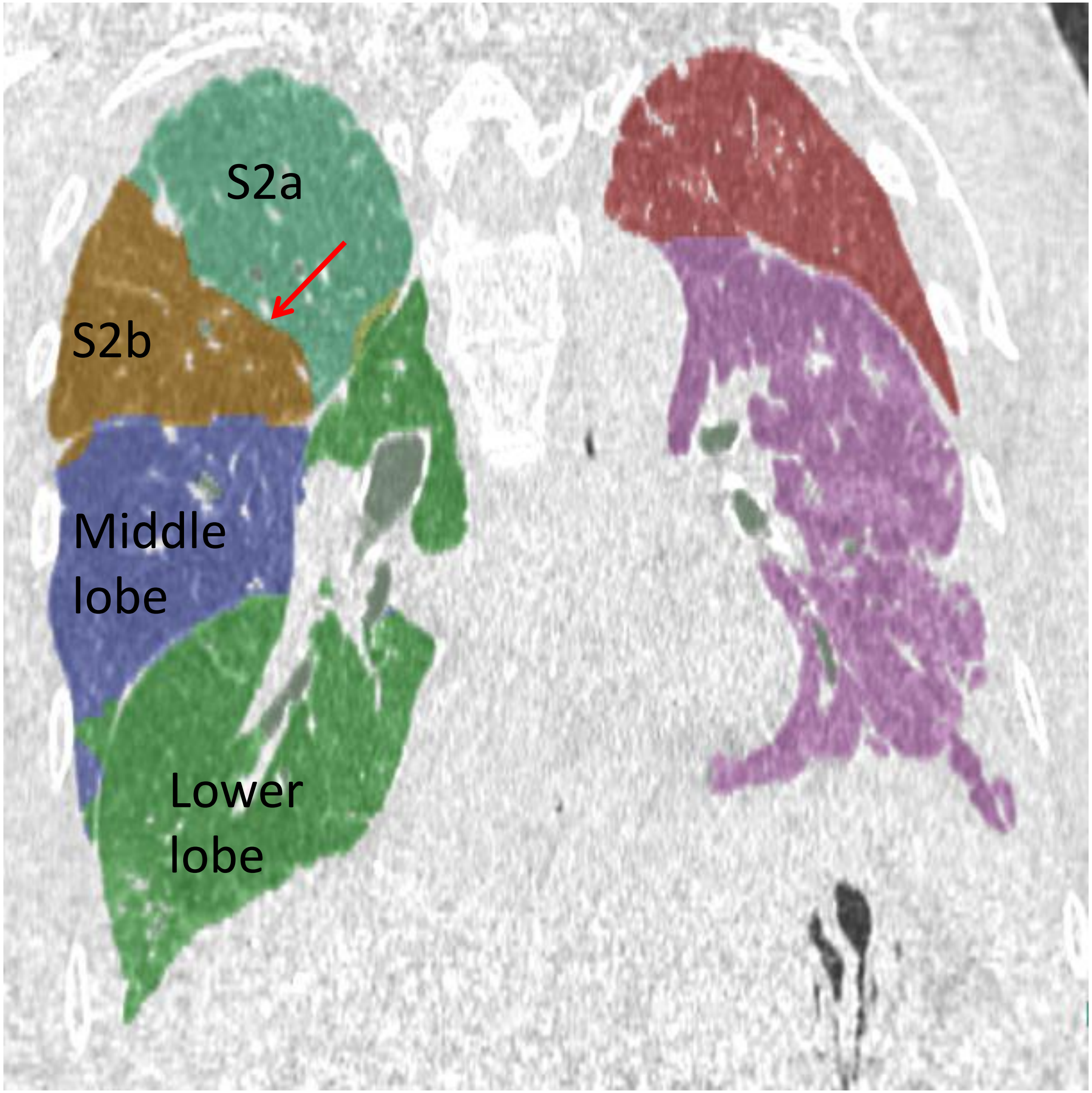

Fig S24a

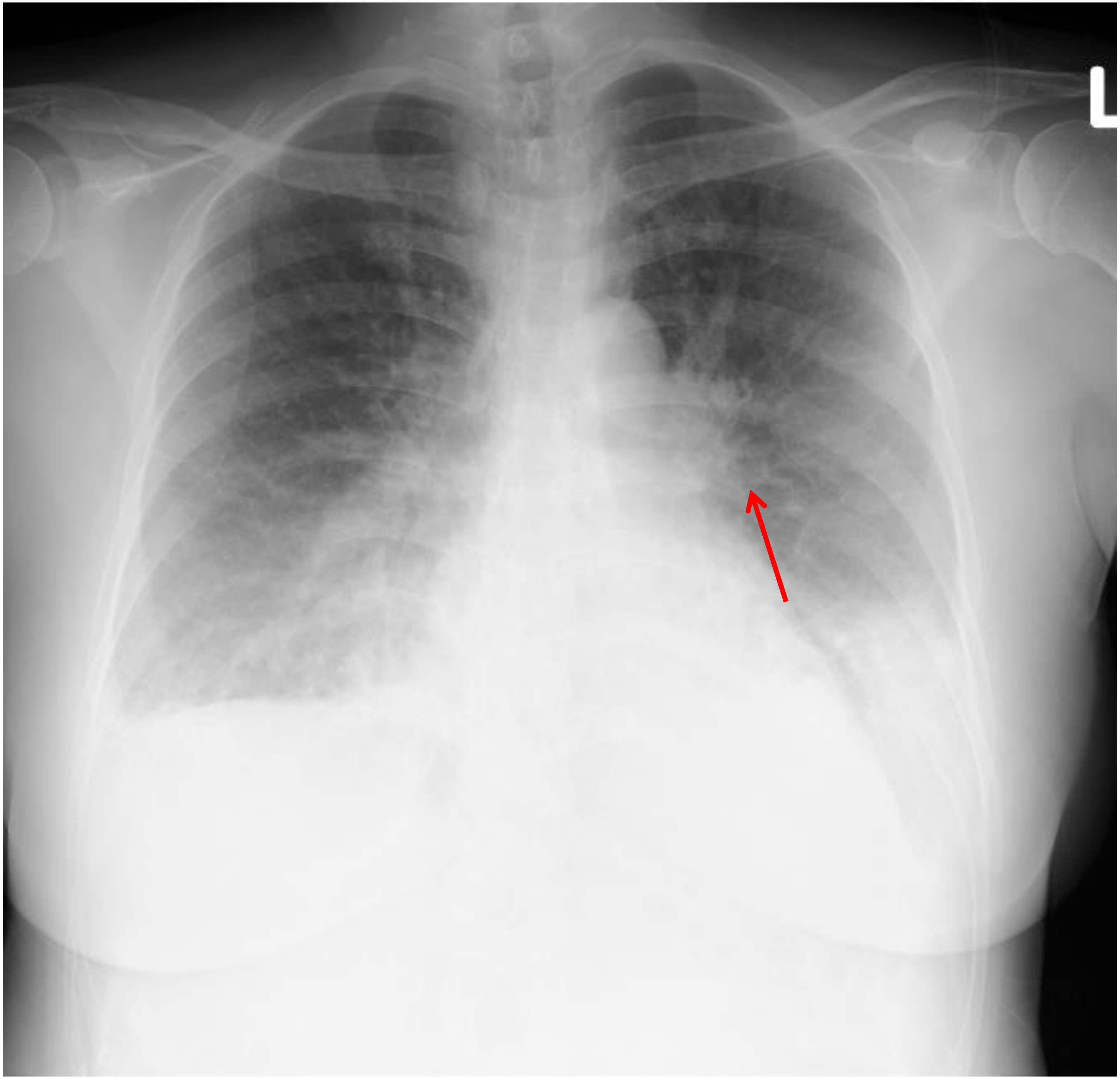

Fig S24b

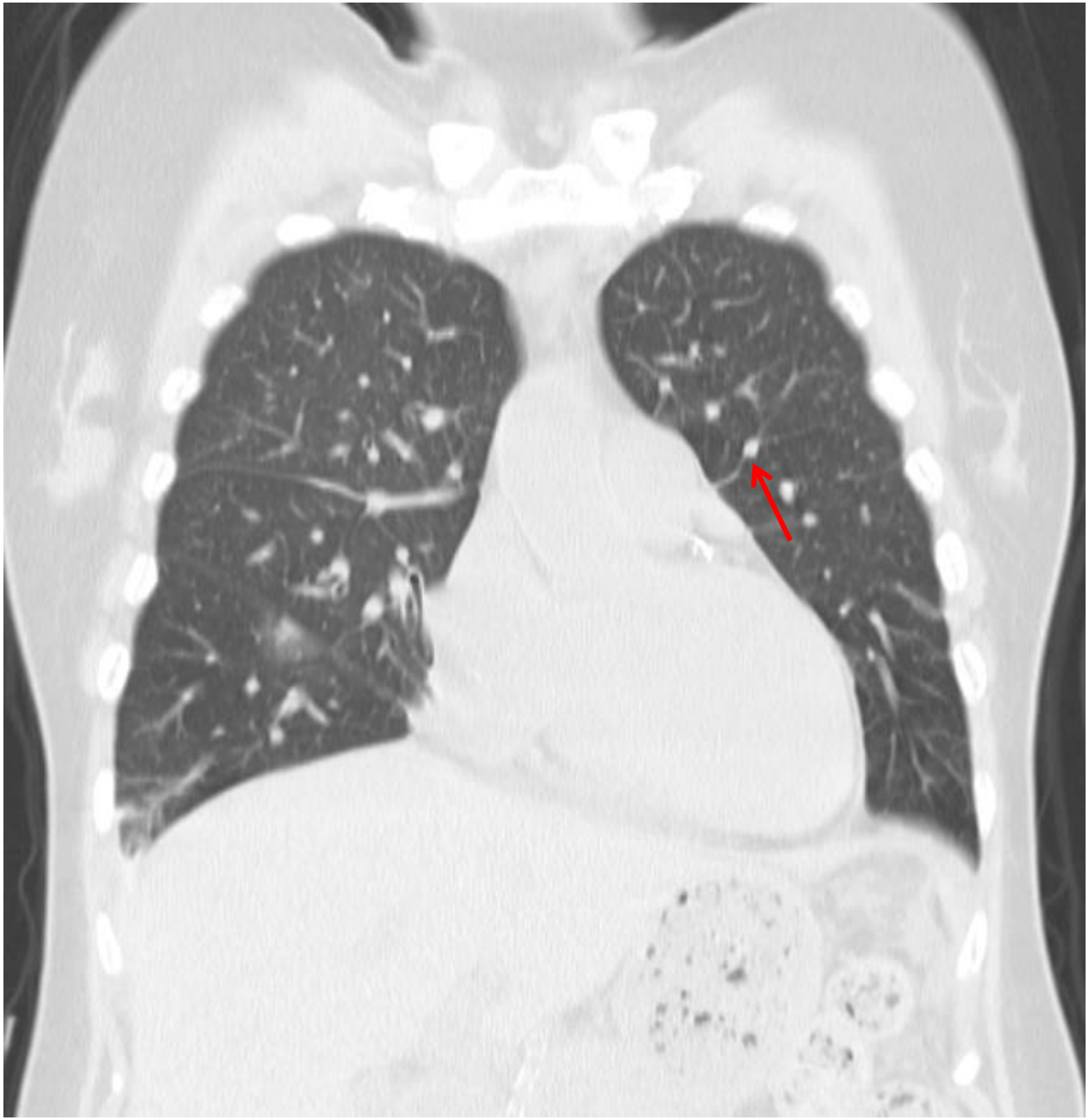

Fig S24c

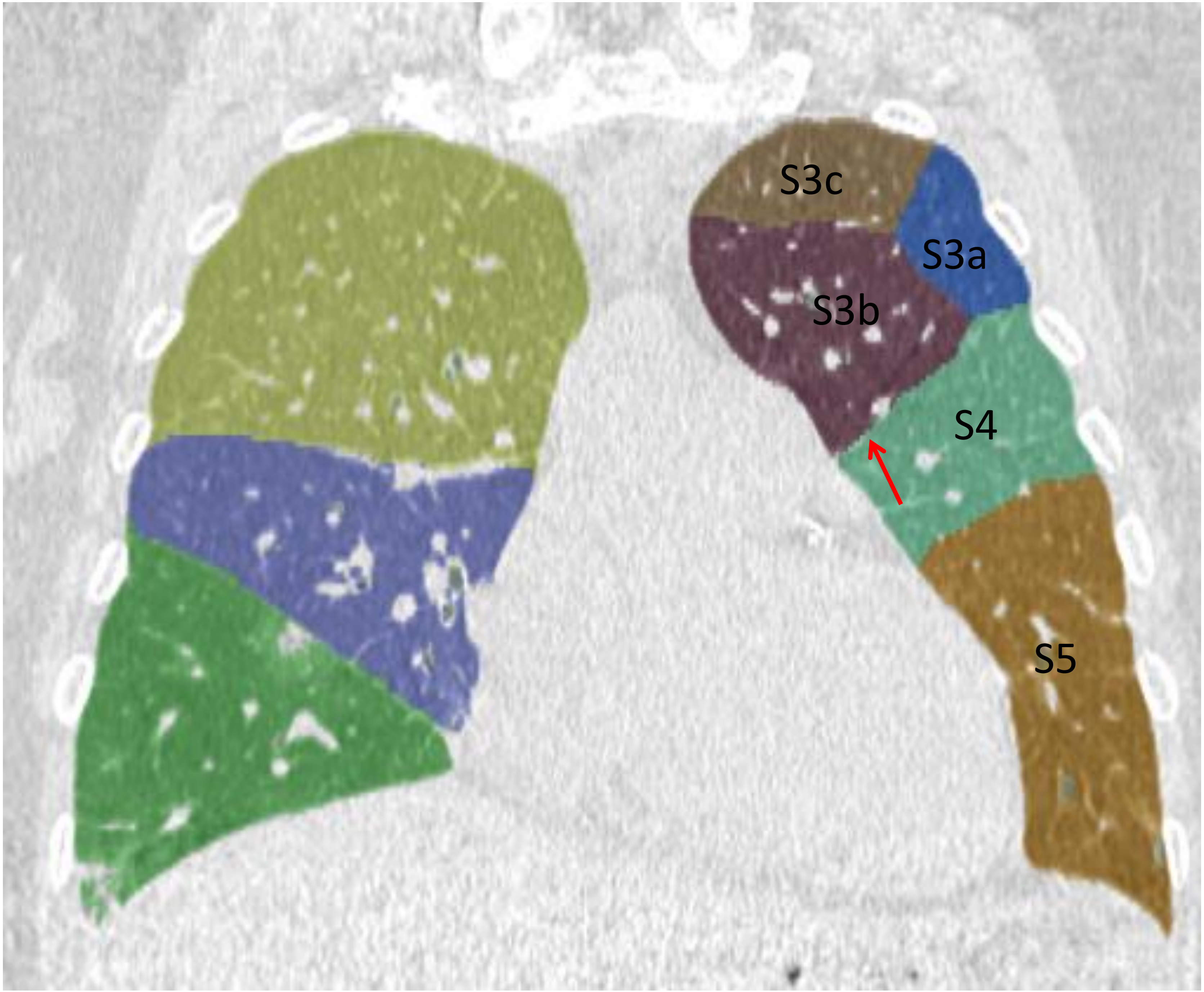

Fig S25a

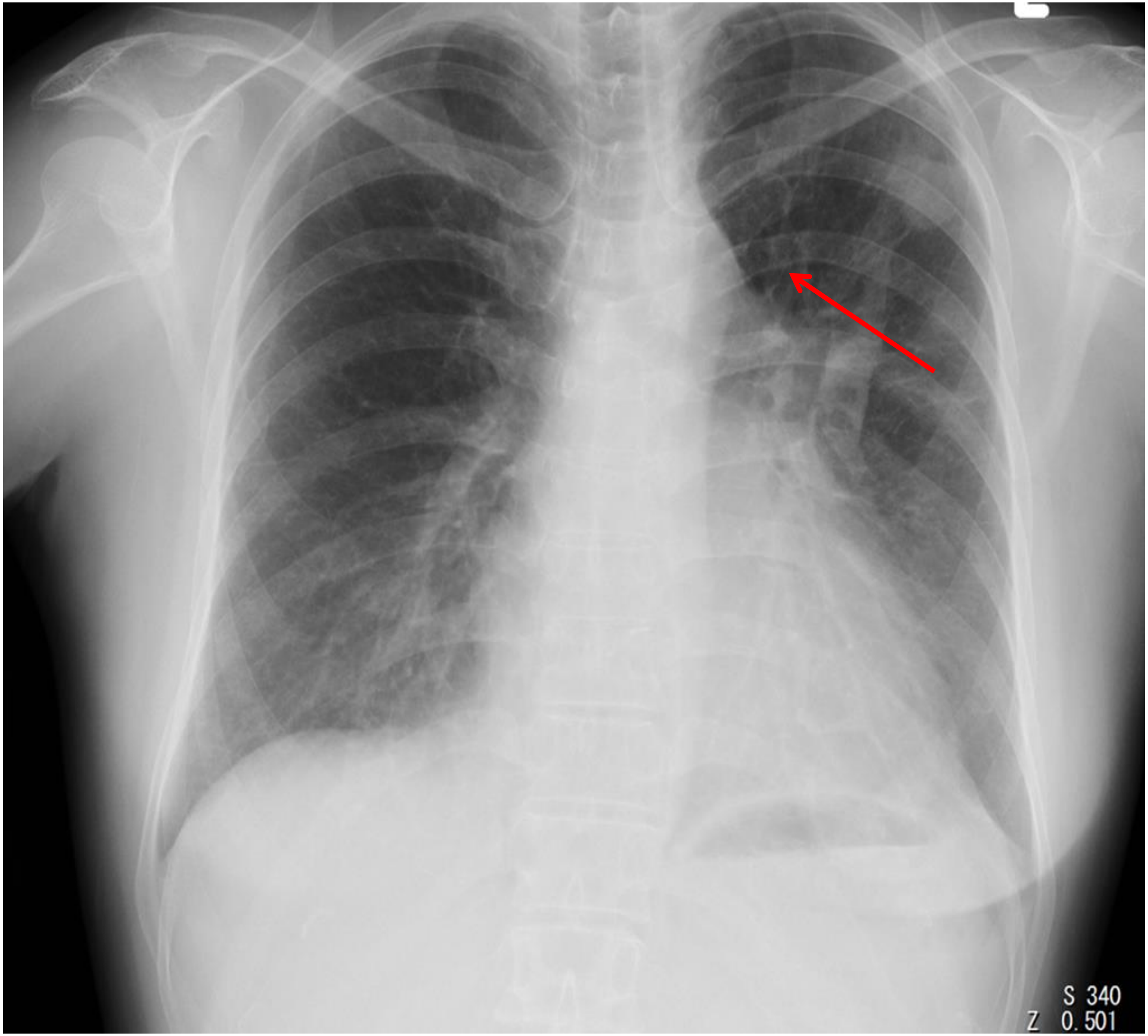

Fig S25b

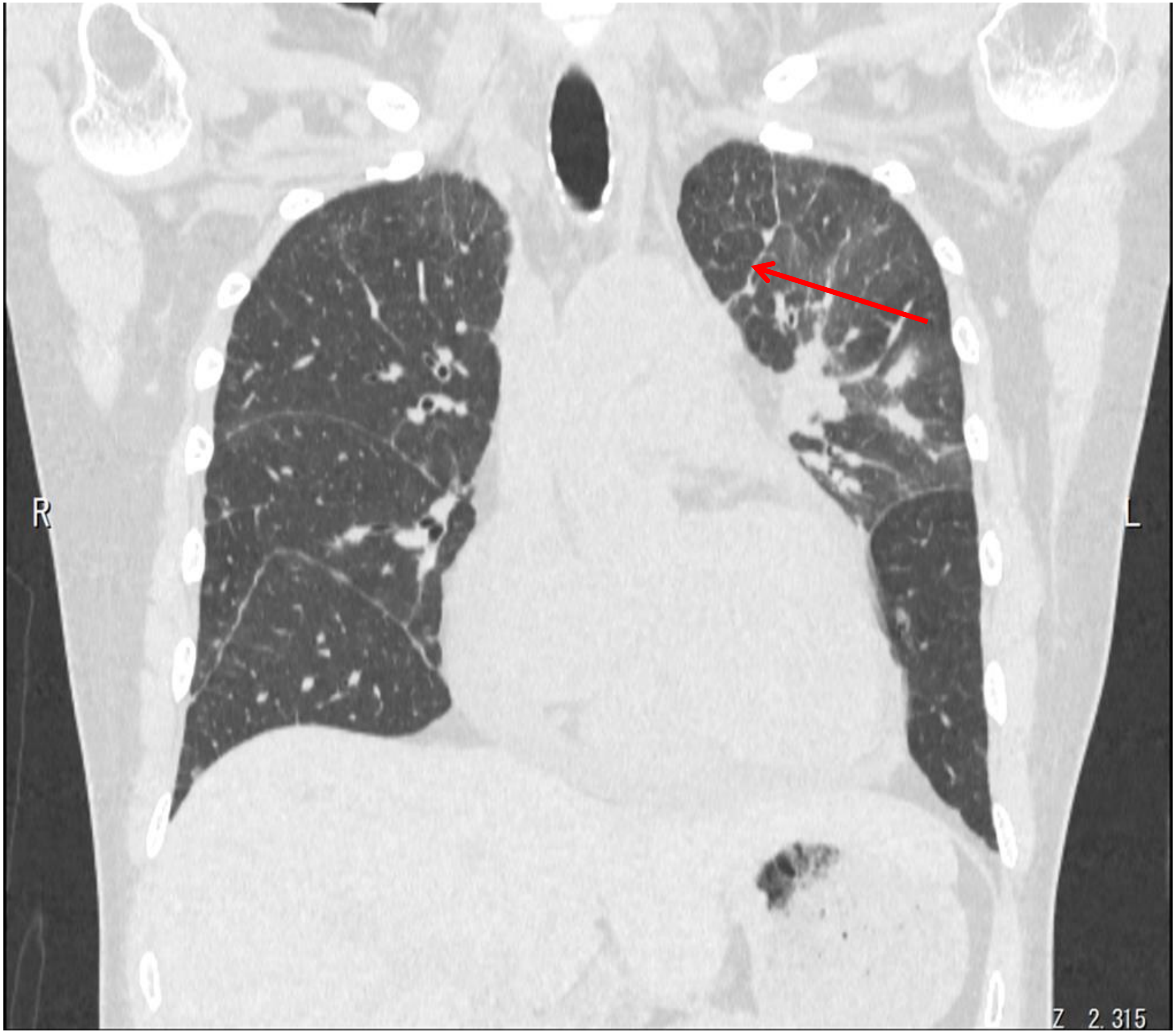

Fig S25c

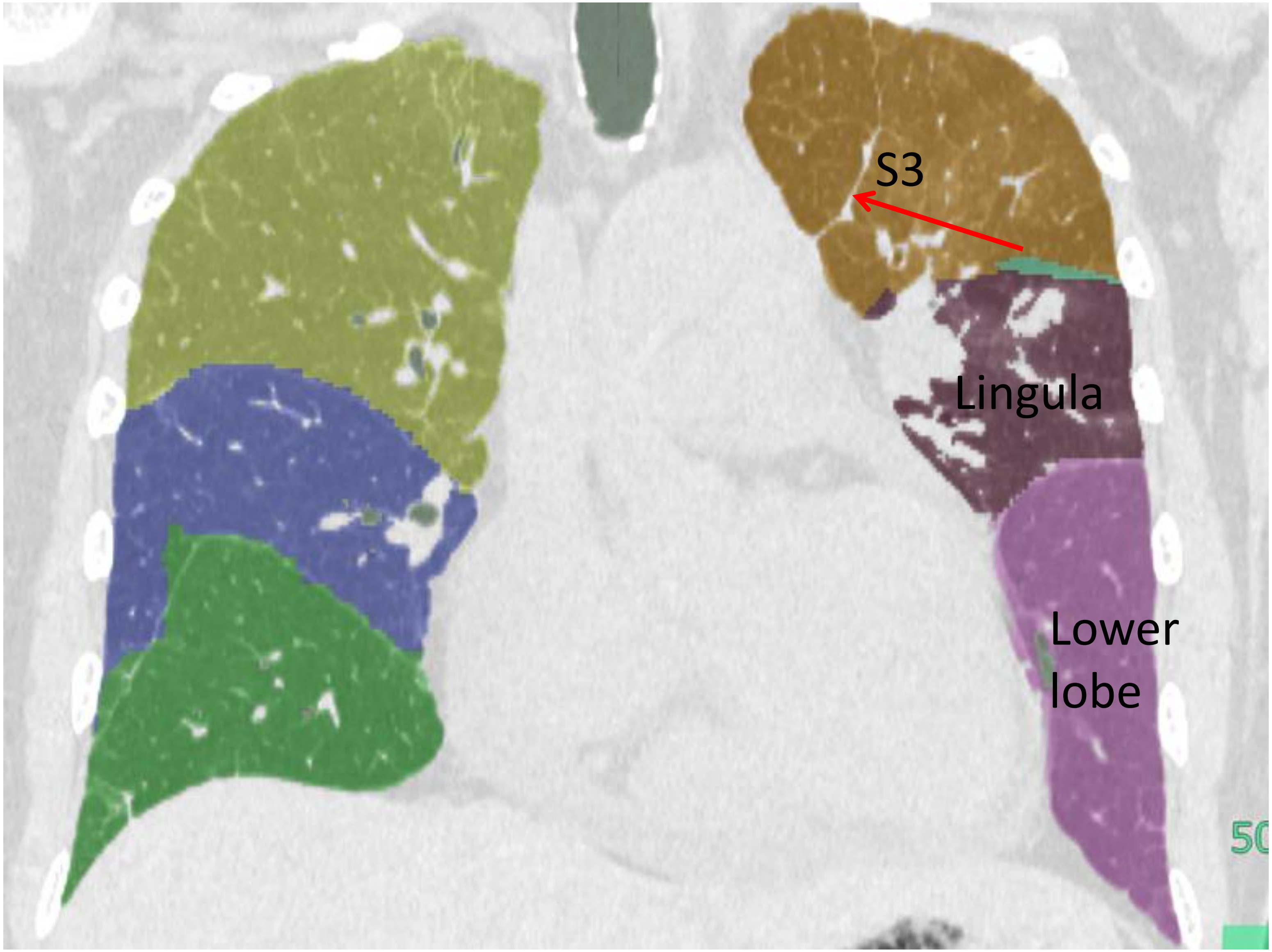

Supplement: Supplementary file 1 — Supplementary file1 (PDF 3747 KB) [file 11604_2021_1215_MOESM1_ESM.pdf]
